# Synthesis and In Silico Analysis of New Polyheterocyclic Molecules Derived from [1,4]-Benzoxazin-3-one and Their Inhibitory Effect against Pancreatic α-Amylase and Intestinal α-Glucosidase

**DOI:** 10.3390/molecules29133086

**Published:** 2024-06-28

**Authors:** Mohamed Ellouz, Aziz Ihammi, Abdellah Baraich, Ayoub Farihi, Darifa Addichi, Saliha Loughmari, Nada Kheira Sebbar, Mohamed Bouhrim, Ramzi A. Mothana, Omar M. Noman, Bruno Eto, Fatiha Chigr, Mohammed Chigr

**Affiliations:** 1 Laboratory of Molecular Chemistry, Materials and Catalysis (LCMMC), Faculty of Sciences and Technology, Sultan Moulay Slimane University, P.O. Box 523, Beni-Mellal 23000, Morocco; darifaadd@gmail.com (D.A.); salihaloughmari@gmail.com (S.L.); chigrm@gmail.com (M.C.); 2 Laboratory of Bioresources, Biotechnology, Ethnopharmacology and Health, Faculty of Sciences, Mohammed First University, Boulevard Mohamed VI, P.O. Box 717, Oujda 60000, Morocco; abdellah.baraich@ump.ac.ma; 3 Laboratory of Biology and Health, Faculty of Sciences, Ibn Tofail University, Kenitra 14000, Morocco; ayoub.farihi@uit.ac.ma; 4 Oriental Center for Water and Environmental Sciences and Technologies (COSTE), Mohammed Premier University, Oujda 60000, Morocco; 5 Laboratory of Organic and Physical Chemistry, Applied Bioorganic Chemistry Team, Faculty of Sciences, Ibnou Zohr University, Agadir 80000, Morocco; n.sebbar@uiz.ac.ma; 6 Biological Engineering Laboratory, Faculty of Sciences and Techniques, Sultan Moulay Slimane University, Beni Mellal 23000, Morocco; mohamed.bouhrim@gmail.com (M.B.); f.chigr@usms.ma (F.C.); 7 Laboratoires TBC, Laboratory of Pharmacology, Pharmacokinetics, and Clinical Pharmacy, Faculty of Pharmaceutical and Biological Sciences, P.O. Box 83, F-59000 Lille, France; bruno.eto@univ-lille.fr; 8 Department of Pharmacognosy, College of Pharmacy, King Saud University, P.O. Box 2457, Riyadh 11451, Saudi Arabia; rmothana@ksu.edu.sa (R.A.M.); onoman@ksu.edu.sa (O.M.N.)

**Keywords:** [1,4]-benzoxazin-3-one, 1,2,3-triazole, isoxazoline, click chemistry, 1,3-dipolar cycloaddition, in silico molecular docking, α-amylase, α-glucosidase

## Abstract

This study focuses on synthesizing a new series of isoxazolinyl-1,2,3-triazolyl-[1,4]-benzoxazin-3-one derivatives **5a**–**5o**. The synthesis method involves a double 1,3-dipolar cycloaddition reaction following a “click chemistry” approach, starting from the respective [1,4]-benzoxazin-3-ones. Additionally, the study aims to evaluate the antidiabetic potential of these newly synthesized compounds through in silico methods. This synthesis approach allows for the combination of three heterocyclic components: [1,4]-benzoxazin-3-one, 1,2,3-triazole, and isoxazoline, known for their diverse biological activities. The synthesis procedure involved a two-step process. Firstly, a 1,3-dipolar cycloaddition reaction was performed involving the propargylic moiety linked to the [1,4]-benzoxazin-3-one and the allylic azide. Secondly, a second cycloaddition reaction was conducted using the product from the first step, containing the allylic part and an oxime. The synthesized compounds were thoroughly characterized using spectroscopic methods, including ^1^H NMR, ^13^C NMR, DEPT-135, and IR. This molecular docking method revealed a promising antidiabetic potential of the synthesized compounds, particularly against two key diabetes-related enzymes: pancreatic α-amylase, with the two synthetic molecules **5a** and **5o** showing the highest affinity values of 9.2 and 9.1 kcal/mol, respectively, and intestinal α-glucosidase, with the two synthetic molecules **5n** and **5e** showing the highest affinity values of −9.9 and −9.6 kcal/mol, respectively. Indeed, the synthesized compounds have shown significant potential as antidiabetic agents, as indicated by molecular docking studies against the enzymes α-amylase and α-glucosidase. Additionally, ADME analyses have revealed that all the synthetic compounds examined in our study demonstrate high intestinal absorption, meet Lipinski’s criteria, and fall within the required range for oral bioavailability, indicating their potential suitability for oral drug development.

## 1. Introduction

**Figure 1:**
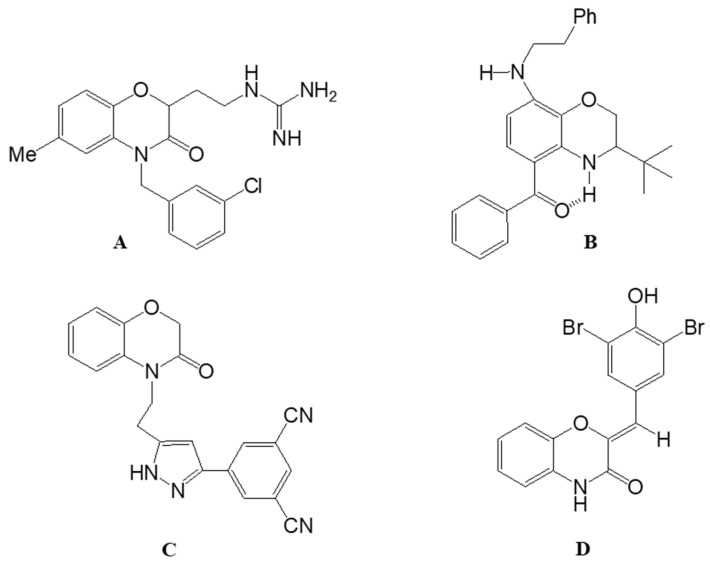
Examples of some bioactive molecules derived from [1,4]-benzoxazine-3-one.

The benzoxazine structure has been thoroughly investigated in both academic and industrial settings. These compounds demonstrate a wide array of biological activities, indicating that the benzoxazine core could be a valuable scaffold in pharmaceutical research and therapeutic applications, including antifungal [1], antidiabetic [2], antimicrobial [3,4], anticancer [5,6], anti-inflammatory [7], antioxidant [8], antiviral [9], and antiherpetic [10] properties. Additionally, a literature review has identified several 1,4-benzoxazine and [1,4]-benzoxazinone-based compounds (Figure 1) in the developmental stage as potential new medications. For example, antibacterial agent **A** acts as an inhibitor of bacterial histidine protein kinase [11], the derivative **B** of [1,4]-benzoxazine appears to be a new neuroprotective agent, effective in a brain injury model [12], the derivative of [1,4]-benzoxazine **C** demonstrated comparable activity against four human cancer cell lines: MCF-7 (breast), A549 (lung), HeLa (cervical), and PC3 (prostate), when compared to the standard etoposide [13], and the [1,4]-benzoxazine **D** derivative is a protective agent in tissue culture and in vivo models of neurodegeneration [14].

Heterocyclic compounds containing 1,2,3-triazole have garnered the attention of various researchers due to their facile synthesis through the 1,3-dipolar azide–alkyne cycloaddition reaction with a Cu(I) catalyst [15]. This aromatic five-membered heterocyclic moiety has been extensively explored in medicinal chemistry owing to its stability against metabolic degradation, its high dipole moment, and its resistance to different chemical environments, such as oxidative/reductive conditions and acid/base hydrolysis. The combination of 1,2,3-triazole with other molecules, such as chalcone, opens promising prospects for medicinal applications [16,17,18,19]. Organic compounds containing the 1,2,3-triazole core have been identified in several drugs, demonstrating a diversity of biological activities, including anti-HIV [20], antitumor and antibacterial [21], anti-inflammatory [22], antimalarial [23], antifungal [24,25], anticancer [26,27], antimicrobial [28,29], antioxidant [30,31], and antiviral [32]. The 1,4-disubstituted 1,2,3-triazole displays a bio-isosteric effect because its planarity and length are comparable to those of an amide bond.

Isoxazoline is an innovative heterocycle that is gaining increasing interest due to its diverse pharmacological activities, making it a primary focus for several research groups worldwide. Primarily recognized for its potential in synthesizing new antibacterial agents [33], it also possesses other highly exciting biological activities, such as antidiabetic [34,35], anticancer [36], antioxidant [37], antimicrobial [38], anti-inflammatory [39], antifungal [40], antiviral [41], and anti-Alzheimer properties [42]. The development of novel isoxazoline derivatives continues to be a major focus in medical research.

Diabetes mellitus is a persistent metabolic disorder marked by elevated levels of glucose in the bloodstream resulting from a dysfunction in the production or function of insulin [43]. Diabetes management is based on regulating blood glucose levels as closely as possible to normal physiological levels to prevent the development of chronic diabetic complications such as retinopathy, nephropathy, and neurological and cardiovascular diseases [44]. Among the strategies for treating diabetic patients is the administration of medications endowed with inhibitory effects on the enzymatic activity of the α-amylase and α-glucosidase enzymes [45,46].

Due to the significant pharmaceutical and biological activities observed with isoxazoline, 1,2,3-triazole, and [1,4]-benzoxazin-3-one, various approaches have been developed to access these molecular structures: [1,4]-benzoxazin-3-one [47,48], 1,2,3-triazole [49], and isoxazoline [50]. On our part, we carried out the first step of the 1,3-dipolar cycloaddition under catalytic conditions to selectively synthesize the 1,2,3-triazole 1,4-disubstituted compound with a good yield, following the method described in the literature [51,52]. Subsequently, we improved the yield of the second step of the cycloaddition [53]. We devised a novel method for synthesizing these [1,4]-benzoxazin-3-one derivatives, achieving satisfactory yields. This endeavor aims to enhance various biological activities such as antidiabetic, anticancer, antioxidant, antiviral, anti-inflammatory, antimicrobial, antifungal, and more. Each of the three components of these newly synthesized molecules, namely [1,4]-benzoxazin-3-one, 1,2,3-triazole, and isoxazoline, exhibit these activities individually. Consequently, the contribution of each of these compounds through its biological activity could eventually lead to very interesting biological activities. After synthesizing these active molecules, we tested their effect on the enzymatic activity of the two enzymes involved in carbohydrate digestion, namely α-amylase and α-glycosidase, in silico.

## 2. Results

### 2.1. Chemical Synthesis of Isoxazolinyl-1,2,3-triazolyl-[1,4]-benzoxazin-3-one Derivatives ***5a**–**5o***

To generate novel heterocyclic systems, we present the synthesis of the isoxazolinyl-1,2,3-triazolyl-[1,4]-benzoxazin-3-one derivatives **5a**–**5o**. This was achieved through a double 1,3-dipolar cycloaddition reaction employing a “click chemistry” strategy, starting from the respective [1,4]-benzoxazin-3-ones [51,52]. To synthesize the 1,2,3-triazole motif 3, the 1,3-dipolar cycloaddition was carried out with good yield using the product 4-(prop-2-yn-1-yl)-2H-[1,4]-benzoxazin-3-one **1**, which contains the propargylic moiety as the dipolarophile, and the allylic azide **2** synthesized through nucleophilic substitution of allyl bromide with sodium azide as the 1,3-dipole. This method is widely employed for obtaining allylic azides [54]. The 1,3-dipolar cycloaddition reaction was conducted at room temperature, under catalytic conditions, in the presence of (${\mathrm{CuSO}}_{4}\cdot 5H_{2}O$ and sodium ascorbate) as the catalyst. For the last step of this work, we were mainly interested in the reactivity of nitriloxides concerning exocyclic carbon-carbon double bonds by carrying out a second 1,3-dipolar cycloaddition, this time between the 4-[(1 -allyl-1H-1,2,3-triazol-4-yl)methyl]-2H-[1,4]-benzoxazine-3-one **3** prepared in the previous step, containing the allylic part and various oximes **4** (4-methylphenyloxime; 2-nitrophenyloxime; 3-nitrophenyloxime; 4-nitrophenyloxime; 4-chlorophenyloxime; benzyloxime; furan-2-yloxime; styryloxime; pyridin-2-yloxime; 1-methyl-1H-pyrrol-2-yloxime; 4-bromophenyloxime; 4-dimethylaminophenyloxime; 4-methoxyphenyloxime; 3-methoxyphenyloxime; 4-fluorophenyloxime) prepared by condensation of different aldehydes and hydroxylamine, following the process described in the literature [53]. However, the dehydrohalogenation of the various oximes by 24° chlorometric bleach generates oximes **4**, which react with the dipolarophile **3** in a two-phase medium (water/chloroform) at a temperature that varies between −5 and 0 °C for 4 h, to lead, respectively, to the cycloadducts **5a**–**5o** with good yield.

All the synthesized compounds were characterized utilizing proton nuclear magnetic resonance (^1^H NMR) and carbon-13 nuclear magnetic resonance (^13^C NMR) spectroscopy (see the experimental section) (Table 1).

**Figure 2:**
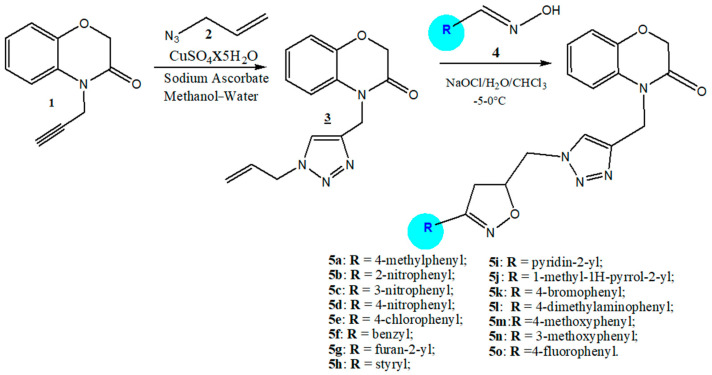
Synthesis of the novel isoxazolinyl-1,2,3-triazolyl-[1,4]-benzoxazin-3-one derivatives **5a**–**5o**.

We see that these 1,3-dipole cycloaddition reactions are totally regioselective because the direction of attack of the dipole is unique (Figure 2). This reaction led to the synthesis of isoxazoline, which constitutes the third heterocyclic component and also exhibits very interesting biological activities.

### 2.2. Computational Analysis Using Molecular Docking

Molecular docking, an advanced computational technique, is frequently utilized to offer valuable insights into the molecular mechanisms of pharmacologically active substances. In this study, molecular docking was employed to unveil the potential mechanism of action associated with the fifteen synthetic molecules’ pancreatic α-amylase and intestinal α-glucosidase activities.

#### 2.2.1. In Silico, Inhibitory Activity of Synthetic Molecules on α-Amylase Activity

The provided data, which comprise binding affinity values, imply that the molecule under study potentially exhibits either a heightened or diminished affinity toward the specified target in comparison with a native ligand, namely acarbose, if a decrease in binding energy correlates with an increase in compound affinity (Table 2). The active sites of pancreatic α-amylase predominantly feature amino acid residues such as Glu A:233 and Asp A:197, A:300, alongside pivotal residues like Arg A:195 and A:337; Trp A:58, A:284, A:203, and A:59; His A:101, A:201, and A:299; Phe A:298, A:265, and A:295; Asn A:298; Gly A:306; Ala A:307; and Tyr A:62 [55,56,57].

**Figure 3:**
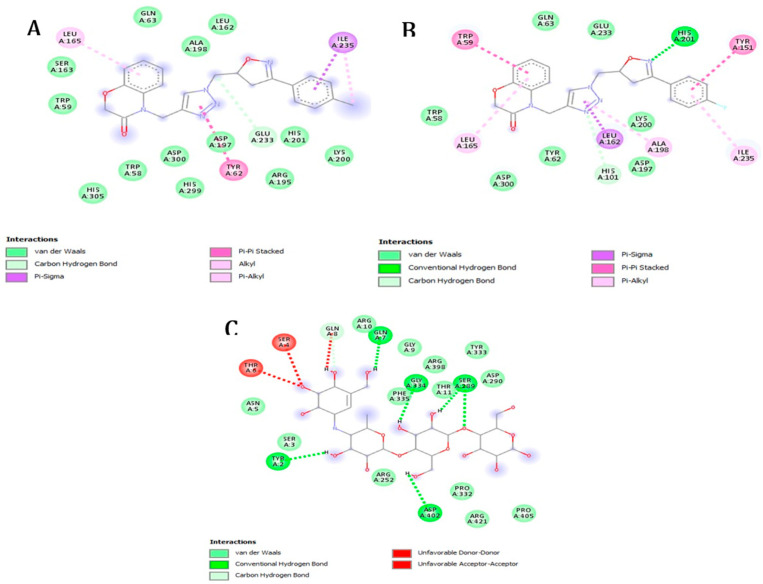
Two-dimensional schemes of the interactions with the amino acid residues of the two potent synthetic molecules, (**A**) **5a**, (**B**) **5o**, and the native ligand, Acarbose (**C**).

Within this context, it is observed that all the examined molecules, except **5n**, exhibit lower free binding energy values compared with the native ligand, suggesting potent inhibitory potential. Molecules **5a** and **5o** demonstrate the lowest free binding energy values, standing at 9.2 and 9.1 kcal/mol, respectively. Notably, both **5a** and **5o** establish electrostatic bonds with amino acid residues surrounding the protein’s active site, primarily in the forms of Pi-sigma, Pi-Pi stacked, and Pi-alkyl interactions (Figure 3). Nevertheless, it is noteworthy that **5o** additionally forms a conventional hydrogen bond with the amino acid residue His A:202. The findings of the computational analysis indicate that the observed antihyperglycemic effects and inhibition of pancreatic α-amylase can be ascribed to these molecules.

#### 2.2.2. In Silico, Inhibitory Activity of Synthetic Molecules on α-Glucosidase Activity

The data presented, in the form of binding affinity values, suggest that the molecule under investigation may exhibit either a heightened or diminished affinity for the specified target in comparison with the native ligand (acarbose), assuming a decrease in binding energy correlates with an increase in the compound’s affinity (Table 3). The active sites of α-glucosidase are primarily surrounded by the amino acid residues Trp A:376, Asp A:404, Leu A:405, Ile A:441, Trp A:481, Asp A:518, Met A:519, Arg A:600, Trp A:613, Asp A:616, Phe A:649, and His A:674 [58].

**Figure 4:**
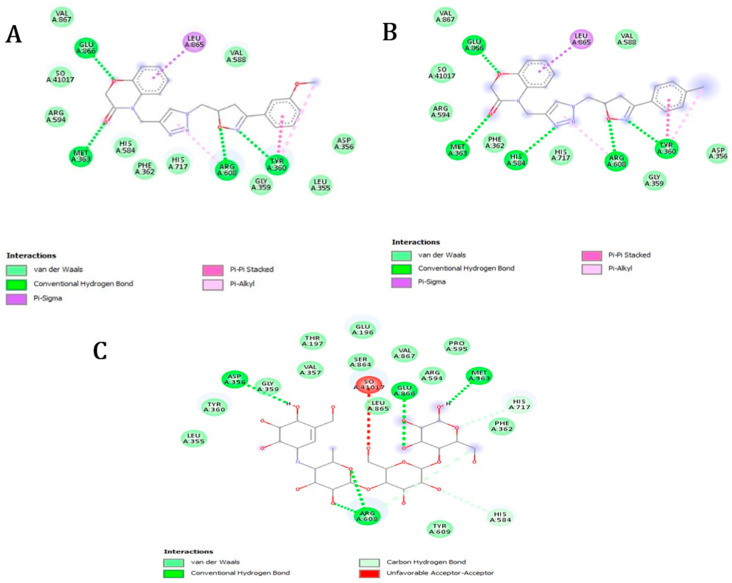
Two-dimensional schemes of the interactions with the amino acid residues of the two potent synthetic molecules, (**A**) **5n**, (**B**) **5e**, and the native ligand, Acarbose (**C**).

Our observations within this framework indicate that all examined molecules exhibit a significant free binding energy compared with the native ligand, ranging from −8.8 to −9.6 kcal/mol (Table 2). Specifically, compounds **5e** and **5n** display the lowest values of free binding energy, at −9.9 and −9.6 kcal/mol, respectively. It is noteworthy that these molecules establish hydrogen bonds (interactions between a hydrogen atom bonded to an electronegative atom and a neighboring electronegative atom) and electrostatic bonds (interactions between oppositely charged entities) with the amino acid residues surrounding the protein’s active site, primarily in the forms of conventional hydrogen bonds, Pi-sigma interactions (bonds between a pi electron and a sigma atom), Pi-Pi stacked interactions (interactions between pi systems), and Pi-alkyl interactions (interactions between a pi system and alkyl groups). Specifically, compound **5n** forms four conventional hydrogen bonds with the amino acid residues Tyr A:360, Met A:363, Arg A:608, and Glu A:866 (Figure 4A), while compound **5e** forms five hydrogen bonds with Tyr A:360, Met A:363, His A:584, Arg A:608, and Glu A:866 (Figure 4B) from the active site of α-glucosidase.

The computational findings suggest that these molecules may contribute to the observed antihyperglycemic effects and the inhibition of pancreatic α-glucosidase, underscoring the significance of hydrogen bonding interactions in modulating enzymatic activity.

### 2.3. ADME Analysis

In silico ADME studies are essential to advancing pharmaceutical development by offering a cost-effective method for predicting how a drug will act within the body [59]. Utilizing computer models for early pharmacokinetic assessment, these studies enable the swift selection of drug candidates and streamline development processes. They help minimize the risk of adverse side effects, decrease the likelihood of drug development failures, and increase the chances of clinical success, making them an invaluable tool in modern drug discovery and development [60]. In order to comply with Lipinski’s rule of five and Veber’s rule, compounds deemed suitable for oral drug development should typically not exceed one violation of the following criteria: (1) no more than 10 hydrogen bond acceptors (nitrogen or oxygen atoms), (2) an octanol-water partition coefficient log P (MLogP) < 5, (3) a molecular mass < 500 daltons, and (4) no more than 5 hydrogen bond donors [61]. In our study, we observed that all the compounds examined met Lipinski’s criteria, indicating their potential suitability for oral drug development.

**Figure 5:**
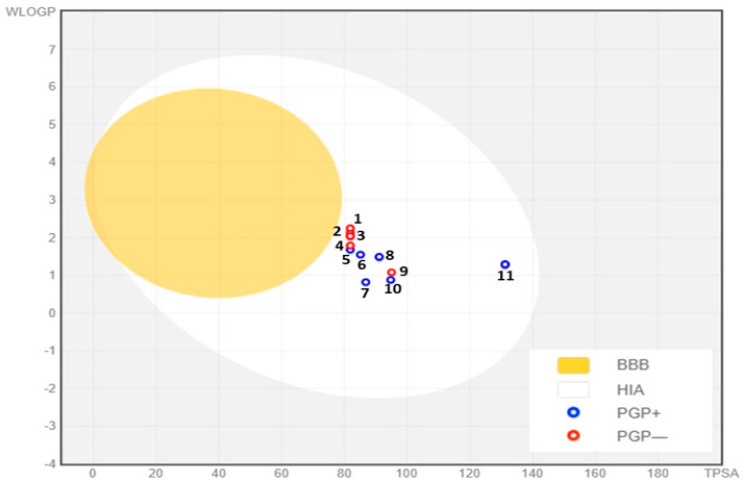
BOILED-Egg Model of the GI absorption and BBB permeability of synthetic molecules (1) **5k**, (2) **5e**, (3) **5o**, (4) **5a**, (5) **5f**, (6) **5l**, (7) **5j**, (8) **5n**, (9) **5g**, (10) **5i**, (11) **5d**. PGP-: non-substrate of P-glycoprotein, PGP+: P-glycoprotein substrate.

**Figure 6:**
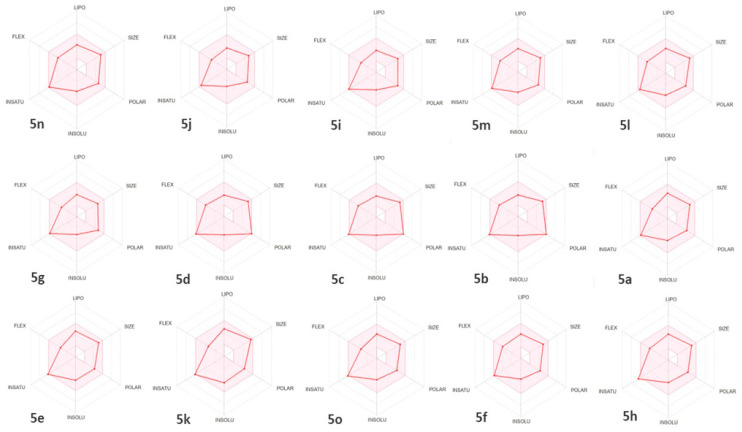
Bioavailability radar of synthetic molecules. The pink region corresponds to the ideal range for each characteristic in terms of oral bioavailability (Lipophilicity, solubility, molecular weight, saturation and flexibility).

The blood–brain barrier (BBB) serves as a crucial barrier between the systemic circulation and the central nervous system, protecting the brain through both biochemical processes, like enzyme reactions, and physical mechanisms such as active expulsion systems [62]. Our investigation revealed that synthetic molecules are unable to cross this barrier, because they have a TPSA > 79 Å^2^, as shown in Table 4 and illustrated in the yellow portion of Figure 5. No molecules disperse in this yellow area. Additionally, most of the compounds analyzed were determined to be P-glycoprotein non-substrates (PGP-), except for **5f**, **5l**, **5j**, **5n**, **5i**, and **5d**, which were determined to be PGP+ (Figure 4). A molecule that is strongly absorbed by the intestine offers significant advantages in terms of bioavailability, efficacy, convenience, and tolerance, making it a promising candidate for the development of oral medications [63]. Notably, all the synthetic compounds examined in our study demonstrate high intestinal absorption. Cytochrome P450 is a crucial enzyme for detoxification, primarily located in the liver [64]. Our analysis identified that the majority of the compounds are neither inhibitors nor substrates of CYP450 enzymes, particularly CYP1A2, except **5i**, **5m**, **5g**, **5a**, **5e**, and **5k**, which were identified as CYP1A2 inhibitors (Table 3). This finding implies a lower likelihood of medication metabolism disruption, thereby strengthening the safety profile of the synthetic compounds. Figure 6 displays the bioavailability profiles of these drugs. The pink zone in these radar graphs corresponds to the oral bioavailability space. A chemical’s characteristics must entirely fall within this specified region to qualify as drug-like [65]. In the present study, all the synthetic compounds meet the required range for oral bioavailability, suggesting their potential as drug candidates.

## 3. Materials and Methods

### 3.1. General

Merck-60 silica gel (230–400 mesh E) was employed for column chromatography. Melting points for compounds **3** and **5a**–**5o** were measured using a Kofler bench (FST, Beni Mellal, Morocco). Reaction progress was tracked with thin-layer chromatography (TLC) on aluminum plates coated with silica gel 60 F254 (E. Merck). Nuclear magnetic resonance (NMR) spectra were obtained on a Varian Unity Plus spectrometer (CNRST, Rabat, Morocco)at 500 MHz for ^1^H NMR and at 125.76 MHz for ^13^C NMR. Chemical shifts were given in parts per million (ppm), with coupling constants (J) noted in Hertz (Hz). The signals were characterized as s (singlet), d (doublet), t (triplet), and m (multiplet), and tetramethylsilane $\mathrm{Si}{\left({\mathrm{CH}}_{3}\right)}_{4}$ was used as the reference.

### 3.2. Procedure for the Preparation of Compound ***3*** by “Click Chemistry” (CuAAC)

A solution of 1 mmol of compound **1** and 2 mmol of 3-azidoprop-1-ene **2** in 8 mL of methanol was prepared, to which 1 mmol of sodium ascorbate and 1 mmol of CuSO_4_$\cdot 5H_{2}O$ dissolved in 7 mL of distilled water were added. The reaction mixture was stirred at room temperature for 3 h and monitored using TLC. Following filtration and concentration under decreased pressure, the resultant substance was then submitted to column chromatography on silica gel, utilizing a mixture of ethyl acetate and hexane at a ratio of 3 to 7 as the eluting solvent. Compound **3** was obtained with a good yield of 81%.

4-[(1-Allyl-1H-1,2,3-triazol-4-yl)methyl]-2H-[1,4]-benzoxazin-3-one (**3**): Brown oil, yield (81%); ^1^H NMR (500 MHz, DMSO-d_6_): δ 7.95 (s, 1H), 6.95–7.29 (m, 4H), 5.92–6.01 (m, 1H), 5.20, 5.08 (dd, 2H, *J* = 10, 17 Hz), 5.11 (s, 2H), 4.94 (d, 2H, *J* = 6 Hz), 4.66 (s, 2H). ^13^C NMR (125 MHz, DMSO-d_6_): δ 164.62, 145.35, 128.95, 128.90, 124.21, 124.12, 133.16, 123.20, 119.31, 117.06, 116.31, 67.67, 52.23, 36.7 (See Figures S1–S3 (Supplementary Materials: spectrum ^1^H, ^13^C NMR, and DEPT-135 for **3**)).

### 3.3. General Procedure for the Synthesis of Compounds ***5a**–**5o***

To a solution of 4-[(1-allyl-1H-1,2,3-triazol-4-yl)methyl]-2H-[1,4]-benzoxazin-3-one **3** (1 mmol) in 8 mL of chloroform, 3 equivalents of oximes: (4-methylphenyloxime; 2-nitrophenyloxime; 3-nitrophenyloxime; 4-nitrophenyloxime; 4-chlorophenyloxime; benzyloxime; furan-2-yloxime; styryloxime; pyridin-2-yloxime; 1-methyl-1H-pyrrol-2-yloxime; 4-bromophenyloxime; 4-dimethylaminophenyloxime; 4-methoxyphenyloxime; 3-methoxyphenyloxime; 4-fluorophenyloxime) were added with vigorous stirring. The mixture was brought to a temperature between −5 and 0 °C, and then 15 mL of NaOCl sodium hypochlorite (bleach 24°) was added dropwise. The progress of the reaction was checked using thin-layer chromatography (TLC). After 9 h of stirring, the organic layer was separated and dried with ${\mathrm{Na}}_{2}{\mathrm{SO}}_{4}$. Subsequently, the solvent was evaporated using reduced pressure. The obtained residue was purified using column chromatography on silica gel utilizing a gradient of hexane and ethyl acetate.

4-[(1-[(3-(4-Methylphenyl)-4,5-dihydroisoxazolin-5-yl)methyl]-1H-1,2,3-triazol-4-yl)methyl]-2H-[1,4]-benzoxazin-3-one (**5a**): Colorless solid, yield (82%); m.p. 191–193 °C; IR (KBr, v_max_/cm^−1^): (C=O lactame str.). ^1^H NMR (500 MHz, DMSO-d_6_): δ 8.02 (s, 1H), 6.96–7.45 (m, 8H), 5.10 (s, 2H), 5.02–5.07 (m, 1H), 4.65 (s, 2H), 4.52 (dd, 2H, *J* = 6.5, 14.5 Hz), 3.50, 3.20 (dd, 2H, *J* = 6.5, 17 Hz), 2.30 (s, 3H). ^13^C NMR (125 MHz, DMSO-d_6_): δ 164.66, 157.23, 145.37, 142.88, 140.54, 128.92, 126.68, 129.86, 127.10, 125.03, 124.18, 123.15, 117.09, 116.27, 79.22, 67.50, 52.84, 37.99, 36.97, 21.61. Elemental analysis calculated (%) for C_22_H_21_N_5_O_3_·1/10 H_2_O: C 65.21, H 5.27, N 17.28, O 12.24; found C 65.50, H 5.25, N 17.36, O 11.90 (See Figures S4–S7 (Supplementary Materials: spectrum ^1^H, ^13^C NMR, DEPT-135, and IR for **5a**)).

4-[(1-[(3-(2-Nitrophenyl)-4,5-dihydroisoxazolin-5-yl)methyl]-1H-1,2,3-triazol-4-yl)methyl]-2H-[1,4]-benzoxazin-3-one (**5b**): Colorless solid, yield (81%); m.p. 152–154 °C; ^1^H NMR (500 MHz, DMSO-d_6_): δ 8.04 (s, 1H), 6.96–7.99 (m, 8H), 5.15–5.20 (m, 1H), 5.12 (s, 2H), 4.66 (s, 2H), 4.56 (dd, 2H, *J* = 6.5, 14.5 Hz), 3.52, 3.16 (dd, 2H, *J* = 6.5, 17.5 Hz). ^13^C NMR (125 MHz, DMSO-d_6_): δ 164.64, 155.27, 148.41, 145.35, 142.94, 128.96, 123.45, 133.73, 131.77, 131.11, 125.08, 124.85, 124.17, 123.16, 117.04, 116.31, 79.63, 67.61, 52.72, 38.82, 36.76. Elemental analysis calculated (%) for C_21_H_18_N_6_O_5_·1/10H_2_O·3/20C_6_H_14_: C 58.56, H 4.56, N 18.71, O 18.17; found C 58.06, H 4.18, N 19.35, O 18.42 (See Figures S8–S10 (Supplementary Materials: spectrum ^1^H, ^13^C NMR, and DEPT-135 for **5b**)).

4-[(1-[(3-(3-Nitrophenyl)-4,5-dihydroisoxazolin-5-yl)methyl]-1H-1,2,3-triazol-4-yl)methyl]-2H-[1,4]-benzoxazin-3-one (**5c**): Pale yellow solid, yield (83%); m.p. 196–198 °C; ^1^H NMR (500 MHz, DMSO-d_6_): δ 8.03 (s, 1H), 6.92–8.28 (m, 8H), 5.16–5.20 (m, 1H), 5.09 (s, 2H), 4.64 (s, 2H), 4.58 (dd, 2H, *J* = 6.5, 14.5 Hz), 3.63, 3.29 (dd, 2H, *J* = 6.5, 17 Hz). ^13^C NMR (125 MHz, DMSO-d_6_): δ 164.61, 156.20, 148.45, 145.54, 142.93, 128.86, 123.06, 133.35, 130.97, 125.20, 125.07, 124.17, 123.16, 121.42, 116.93, 116.25, 79.96, 67.59, 52.80, 37.59, 36.74. Elemental analysis calculated (%) for C_21_H_18_N_6_O_5_·1/10H_2_O·1/5C_6_H_14_: C 58.80, H 4.67, N 18.53, O 18.00; found C 58.06, H 4.18, N 19.35, O 18.42 (See Figures S11–S13 (Supplementary Materials: spectrum ^1^H, ^13^C NMR, and DEPT-135 for **5c**)).

4-[(1-[(3-(4-Nitrophenyl)-4,5-dihydroisoxazolin-5-yl)methyl]-1H-1,2,3-triazol-4-yl)methyl]-2H-[1,4]-benzoxazin-3-one (**5d**): Colorless solid, yield (82%); m.p. 262–264 °C; IR (KBr, v_max_/cm^−1^): 1678 (C=O lactame str.). ^1^H NMR (500 MHz, DMSO-d_6_): δ 8.02 (s, 1H), 6.96–8.20 (m, 8H), 5.17–5.22 (m, 1H), 5.08 (s, 2H), 4.64 (s, 2H), 4.60 (dd, 2H, *J* = 6.5, 14 Hz), 3.57, 3.29 (dd, 2H, *J* = 6.5, 17 Hz). ^13^C NMR (125 MHz, DMSO-d_6_): δ 164.64, 156.41, 145.19, 142.83, 135.26, 128.89, 128.3, 129.40, 128.83, 125.07, 124.04, 123.07, 116.94, 116.14, 79.46, 67.50, 52.71, 37.81, 36.66. Elemental analysis calculated (%) for C_21_H_18_N_6_O_5_·1/10CH_3_CO_2_C_2_H_5_: C 57.99, H 4.28, N 18.96, O 18.77; found C 58.06, H 4.18, N 19.35, O 18.42 (See Figures S14–S17 (Supplementary Materials: spectrum ^1^H, ^13^C NMR, DEPT-135, and IR for **6d**)).

4-[(1-[(3-(4-Chlorophenyl)-4,5-dihydroisoxazolin-5-yl)methyl]-1H-1,2,3-triazol-4-yl)methyl]-2H-[1,4]-benzoxazin-3-one (**5e**): White powder, yield (83%); m.p. 172–174 °C; ^1^H NMR (500 MHz, DMSO-d_6_): δ 8.01 (s, 1H), 6.93–7.57 (m, 8H), 5.10 (s, 2H), 5.10–5.14 (m, 1H), 4.64 (s, 2H), 4.54 (dd, 2H, *J* = 6.5, 14.5 Hz), 3.52, 3.20 (dd, 2H, *J* = 6.5, 17 Hz). ^13^C NMR (125 MHz, DMSO-d_6_): δ 164.66, 156.50, 145.39, 142.89, 135.34, 128.94, 128.34, 129.40, 128.83, 125.09, 124.22, 123.15, 117.03, 116.26, 79.46, 67.60, 52.78, 37.81, 36.70. Elemental analysis calculated (%) for C_21_H_18_ClN_5_O_3_·1/10H_2_O: C 59.26, H 4.31, N 16.45, O 11.65; found C 59.51, H 4.28, N 16.52, O 11.32 (See Figures S18–S20 (Supplementary Materials: spectrum ^1^H, ^13^C NMR, and DEPT-135 for **5e**)).

4-[(1-[(3-Benzyl-4,5-dihydroisoxazolin-5-yl)methyl]-1H-1,2,3-triazol-4-yl)methyl]-2H-[1,4]-benzoxazin-3-one (**5f**): Colorless solid, yield (78%); m.p. 142–144 °C; ^1^H NMR (500 MHz, DMSO-d_6_): δ 7.94 (s, 1H), 6.95–7.29 (m, 9H), 5.11 (s, 2H), 4.82–4.87 (m, 1H), 4.66 (s, 2H), 4.38 (dd, 2H, *J* = 6.5, 14 Hz), 3.52 (s, 2H), 3.35, 2.95 (dd, 2H, *J* = 6.5, 17.5 Hz). ^13^C NMR (125 MHz, DMSO-d_6_): δ 164.68, 158.64, 145.39, 142.94, 136.36, 128.84, 129.89, 129.16, 127.35, 124.95, 124.25, 123.17, 117.03, 116.32, 78.12, 60.90, 67.62, 52.72, 38.96, 36.80. Elemental analysis calculated (%) for C_22_H_21_N_5_O_3_·1/10H_2_O·1/20C_6_H_14_: C 65.40, H 5.39, N 17.10, O 12.11; found C 65.50, H 5.25, N 17.36, O 11.90 (See Figures S21–S23 (Supplementary Materials: spectrum ^1^H, ^13^C NMR, and DEPT-135 for **5f**)).

4-[(1-[(3-(Furan-2-yl)-4,5-dihydroisoxazolin-5-yl)methyl]-1H-1,2,3-triazol-4-yl)methyl]-2H-[1,4]-benzoxazin-3-one (**5g**): Brown oil, yield (80%); IR (KBr, v_max_/cm^−1^): 1682 (C=O lactame str.). ^1^H NMR (500 MHz, DMSO-d_6_): δ 8.00 (s, 1H), 6.59–7.26 (m, 7H), 5.11 (s, 2H), 5.00–5.06 (m, 1H), 4.66 (s, 2H), 4.53 (dd, 2H, *J* = 6.5, 14 Hz), 3.45, 3.13 (dd, 2H, *J* = 6.5, 17.5 Hz). ^13^C NMR (125 MHz, DMSO-d_6_): δ 164.65, 149.17, 145.37, 144.39, 142.90, 128.92, 145.95, 125.01, 124.12, 123.16, 117.03, 116.30, 113.89, 112.52, 78.87, 67.82, 52.73, 38.14, 36.71. Elemental analysis calculated (%) for C_22_H_21_N_5_O_3_·1/12H_2_O: C 59.92, H 4.54, N 18.39, O 17.15; found C 60.15, H 4.52, N 18.46, O 16.87 (See Figures S24–S27 (Supplementary Materials: spectrum ^1^H, ^13^C NMR, DEPT-135, and IR for **5g**)).

4-[(1-[(3-Styryl-4,5-dihydroisoxazolin-5-yl)methyl]-1H-1,2,3-triazol-4-yl)methyl]-2H-[1,4]-benzoxazin-3-one (**5h**): Pale yellow solid, yield (82%); m.p. 174–176 °C; ^1^H NMR (500 MHz, DMSO-d_6_): δ 8.01 (s, 1H), 6.92–7.57 (m, 9H), 6.89, 6.98 (d, 2H, *J* = 16.5 Hz), 5.11 (s, 2H), 5.00–5.05 (m, 1H), 4.66 (s, 2H), 4.52 (dd, 2H, *J* = 6.5, 14.5 Hz), 3.34, 3.05 (dd, 2H, *J* = 6.5, 17.5 Hz). ^13^C NMR (125 MHz, DMSO-d_6_): δ 164.68, 158.38, 145.40, 142.90, 136.13, 128.62, 137.69, 129.35, 128.88, 127.68, 124.99, 124.16, 123.14, 117.58, 116.93, 116.26, 79.12, 67.61, 52.87, 39.08, 36.80. Elemental analysis calculated (%) for C_23_H_21_N_5_O_3_·1/11H_2_O: C 66.23, H 5.12, N 16.79, O 11.86; found C 66.49, H 5.09, N 16.86, O 11.55 (See Figures S28–S30 (Supplementary Materials: spectrum ^1^H, ^13^C NMR, and DEPT-135 for **5h**)).

4-[(1-[(3-(Pyridin-2-yl)-4,5-dihydroisoxazolin-5-yl)methyl]-1H-1,2,3-triazol-4-yl)methyl]-2H-[1,4]-benzoxazin-3-one (**5i**): Yellow oil, yield (79%); IR (KBr, v_max_/cm^−1^): 1672 (C=O lactame str.). ^1^H NMR (500 MHz, DMSO-d_6_): δ 8.01 (s, 1H), 6.93–7.81 (m, 8H), 5.15 (s, 2H), 5.08–5.13 (m, 1H), 4.64 (s, 2H), 4.57 (dd, 2H, *J* = 6.5, 14.5 Hz), 3.54, 3.20 (dd, 2H, *J* = 6.5, 17 Hz). ^13^C NMR (125 MHz, DMSO-d_6_): δ 164.65, 149.17, 145.31, 144.37, 142.71, 128.75, 145.75, 125.01, 123.88, 122.69, 117.03, 116.21, 114.54, 113.77, 112.38, 79.02, 67.62, 52.60, 38.36, 36.55. Elemental analysis calculated (%) for C_20_H_18_N_6_O_3_·1/10H_2_O·1/20C_6_H_14_: C 61.49, H 4.80, N 21.20, O 12.51; found C 61.53, H 4.65, N 21.53, O 12.29 (See Figures S31–S34 (Supplementary Materials: spectrum ^1^H, ^13^C NMR, DEPT-135, and IR for **5i**)).

4-[(1-[(3-(1-Methyl-1H-pyrrol-2-yl)-4,5-dihydroisoxazolin-5-yl)methyl]-1H-1,2,3-triazol-4-yl)methyl]-2H-[1,4]-benzoxazin-3-one (**5j**): Colorless solid, yield (78%); m.p. 170–172 °C; IR (KBr, v_max_/cm^−1^): 1678 (C=O lactame str.). ^1^H NMR (500 MHz, DMSO-d_6_): δ 8.00 (s, 1H), 6.03–7.26 (m, 7H), 5.11 (s, 2H), 4.89–4.94 (m, 1H), 4.65 (s, 2H), 4.48 (dd, 2H, *J* = 6.5, 14.5 Hz), 3.69 (s, 3H), 3.46, 3.14 (dd, 2H, *J* = 6.5, 17.5 Hz). ^13^C NMR (125 MHz, DMSO-d_6_): δ 164.86, 151.18, 145.39, 142.89, 128.97, 122.03, 128.71, 124.90, 124.20, 123.15, 117.04, 116.28, 115.11, 108.42, 77.03, 56.16, 67.59, 52.06, 38.35, 36.59. Elemental analysis calculated (%) for C_20_H_20_N_6_O_3_·1/10H_2_O: C 60.94, H 5.16, N 21.32, O 12.58; found C 61.21, H 5.14, N 21.42, O 12.23 (See Figures S35–S38 (Supplementary Materials: spectrum ^1^H, ^13^C NMR, DEPT-135, and IR for **5j**)).

4-[(1-[(3-(4-Bromophenyl)-4,5-dihydroisoxazolin-5-yl)methyl]-1H-1,2,3-triazol-4-yl)methyl]-2H-[1,4]-benzoxazin-3-one (**5k**): White powder, yield (81%); m.p. 183–185 °C; ^1^H NMR (500 MHz, DMSO-d_6_): δ 8.01 (s, 1H), 6.93–7.51 (m, 8H), 5.10 (s, 2H), 4.98–5.03 (m, 1H), 4.65 (s, 2H), 4.51 (dd, 2H, *J* = 6.5, 14 Hz), 3.49, 3.15 (dd, 2H, *J* = 6.5, 17 Hz). ^13^C NMR (125 MHz, DMSO-d_6_): δ 164.62, 156.60, 145.29, 142.80, 132.28, 129.10, 128.66, 132.33, 129.04, 125.06, 124.19, 123.15, 117.04, 116.26, 79.47, 67.60, 52.78, 37.81, 36.69. Elemental analysis calculated (%) for C_21_H_18_BrN_5_O_3_·1/20H_2_O·1/20C_6_H_14_: C 54.03, H 4.00, N 14.79, O 10.31; found C 53.86, H 3.87, N 14.95, O 10.25 (See Figures S39–S41 (Supplementary Materials: spectrum ^1^H, ^13^C NMR, and DEPT-135 for **5k**)).

4-[(1-[(3-(4-Dimethylaminophenyl)-4,5-dihydroisoxazolin-5-yl)methyl]-1H-1,2,3-triazol-4-yl)methyl]-2H-[1,4]-benzoxazin-3-one (**5l**): Brown oil, yield (80%); IR (KBr, v_max_/cm^−1^): 1677 (C=O lactame str.). ^1^H NMR (500 MHz, DMSO-d_6_): δ 8.01 (s, 1H), 6.66–7.38 (m, 8H), 5.09–5.13 (m, 1H), 4.96 (s, 2H), 4.65 (s, 2H), 4.49 (dd, 2H, *J* = 6.5, 14.5 Hz), 3.44, 3.12 (dd, 2H, *J* = 6.5, 17.5 Hz), 2.91 (s, 6H). ^13^C NMR (125 MHz, DMSO-d_6_): δ 164.66, 156.93, 151.87, 145.40, 142.79, 128.92, 116.30, 128.30, 124.98, 124.20, 123.18, 117.04, 116.46, 112.14, 78.34, 67.55, 53.14, 38.37, 36.73, 40.25. Elemental analysis calculated (%) for C_23_H_24_N_6_O_3_·1/20H_2_O·1/20C_6_H_14_·1/25C_4_H_8_O_2_: C 63.86, H 5.74, N 19.05, O 11.35; found C 63.88, H 5.59, N 19.43, O 11.10 (See Figures S42–S45 (Supplementary Materials: spectrum ^1^H, ^13^C NMR, DEPT-135, and IR for **5l**)).

4-[(1-[(3-(4-Methoxyphenyl)-4,5-dihydroisoxazolin-5-yl)methyl]-1H-1,2,3-triazol-4-yl)methyl]-2H-[1,4]-benzoxazin-3-one (**5m**): White powder, yield (82%); m.p. 153–155 °C; IR (KBr, v_max_/cm^−1^): 1679 (C=O lactame str.). ^1^H NMR (500 MHz, DMSO-d_6_): δ 8.01 (s, 1H), 6.93–7.50 (m, 8H), 5.11 (s, 2H), 5.01–5.05 (m, 1H), 4.65 (s, 2H), 4.51 (dd, 2H, *J* = 6.5, 14.5 Hz), 3.76 (s, 3H), 3.49, 3.15 (dd, 2H, *J* = 6.5, 17 Hz). ^13^C NMR (125 MHz, DMSO-d_6_): δ 164.95, 156.63, 161.25, 145.34, 142.85, 128.75, 121.86, 128.94, 125.02, 124.19, 123.16, 117.03, 116.30, 114.74, 78.87, 55.95, 67.61, 52.77, 38.25, 36.64. Elemental analysis calculated (%) for C_22_H_21_N_5_O_4_·1/10H_2_O: C 62.73, H 5.07, N 16.63, O 15.57; found C 63.00, H 5.05, N 16.70, O 15.26 (See Figures S46–S49 (Supplementary Materials: spectrum ^1^H, ^13^C NMR, DEPT-135, and IR for **5m**)).

4-[(1-[(3-(3-Methoxyphenyl)-4,5-dihydroisoxazolin-5-yl)methyl]-1H-1,2,3-triazol-4-yl)methyl]-2H-[1,4]-benzoxazin-3-one (**5n**): Colorless solid, yield (83%); m.p. 182–184 °C; IR (KBr, v_max_/cm^−1^): 1674 (C=O lactame str.). ^1^H NMR (500 MHz, DMSO-d_6_): δ 8.03 (s, 1H), 6.96–7.41 (m, 8H), 5.11 (s, 2H), 5.04–5.07 (m, 1H), 4.65 (s, 2H), 4.55 (dd, 2H, *J* = 6.5, 14 Hz), 3.74 (s, 3H), 3.53, 3.18 (dd, 2H, *J* = 6.5, 17.5 Hz). ^13^C NMR (125 MHz, DMSO-d_6_): δ 164.68, 157.22, 159.86, 145.41, 142.92, 130.73, 128.97, 130.50, 125.06, 124.18, 123.14, 119.67, 117.05, 116.61, 116.27, 112.12, 79.22, 55.74, 67.67, 52.78, 38.03, 36.73. Elemental analysis calculated (%) for C_22_H_21_N_5_O_4_·1/15H_2_O·1/20C_6_H_14_: C 63.03, H 5.18, N 16.48, O 15.31; found C 63.00, H 5.05, N 16.70, O 15.26 (See Figures S50–S53 (Supplementary Materials: spectrum ^1^H, ^13^C NMR, DEPT-135, and IR for **5n**)).

4-[(1-[(3-(4-Fluorophenyl)-4,5-dihydroisoxazolin-5-yl)methyl]-1H-1,2,3-triazol-4-yl)methyl]-2H-[1,4]-benzoxazin-3-one (**5o**): Colorless solid, yield (81%); m.p. 132–134 °C; ^1^H NMR (500 MHz, DMSO-d_6_): δ 8.01 (s, 1H), 6.93–7.90 (m, 8H), 5.10 (s, 2H), 5.05–5.09 (m, 1H), 4.64 (s, 2H), 4.54 (dd, 2H, *J* = 6.5, 14.5 Hz), 3.52, 3.20 (dd, 2H, *J* = 6.5, 17.5 Hz). ^13^C NMR (125 MHz, DMSO-d_6_): δ 167.33, 156.43, 164.60, 145.37, 142.86, 128.94, 125.04, 130.69, 129.71, 124.18, 123.15, 117.03, 116.47, 115.54, 79.27, 67.61, 52.78, 38.05, 36.73. Elemental analysis calculated (%) for C_21_H_18_FN_5_O_3_·1/12H_2_O: C 61.68, H 4.48, N 17.13, O 12.06; found C 61.91, H 4.45, N 17.19, O 11.78 (See Figures S54–S56 (Supplementary Materials: spectrum ^1^H, ^13^C NMR, and DEPT-135 for **5o**)).

### 3.4. Molecular Docking Analysis

The molecular docking analysis was conducted following the guidelines outlined in the reference [66,67,68]. The crystalline structures of α-amylase (PDB ID: 1SMD) and α-glycosidase (PDB ID: 5NN5) were obtained from the RCSB protein database (http://www.rcsb.org/pdb) (accessed on 3 March 2024), established at the Brookhaven National Laboratory in 1971. The removal of water molecules was achieved using AutoDock Tools v1.5.7, while also incorporating polar hydrogens and Kollman charges; co-crystallized ligands were excluded; and the protein was saved in the “pdbqt” format. The two-dimensional configuration of each ligand was converted to the three-dimensional configuration using Avogadro version 1.2.0 software, as depicted in [69,70]. Using AutoDock Tools (version 1.5.6), the final pdbqt file of the ligand was obtained. The grid box representing the docking search space was enlarged to better fit the active binding site. The coordinates of the grid box for the two enzymes, α-amylase and α-glycosidase, were defined as follows: for α-amylase, the centers (x, y, and z) were set at 8.349, 58.705, and 19.096, while for α-glucosidase, the centers (x, y, and z) were fixed at 1.591, −26.522, and 87.364, with a uniform grid box size maintained at 40. The results for the docked ligand complexes were expressed as ΔG binding energy values in kcal/mol. Acarbose, an agent with a history of 30 years in treating type 2 diabetes, is utilized to prevent postprandial hyperglycemia by blocking carbohydrate digestion in the small intestine. In this computational section of the investigation, acarbose was employed as the native ligand. The process of generating 2D molecular interaction diagrams and examining protein-ligand binding interactions was carried out using Discovery Studio 4.1 (Dassault Systems Biovia, San Diego, CA, USA).

### 3.5. ADME Studies

Understanding pharmacokinetic properties, including absorption, distribution, metabolism, and excretion (ADME), is essential for comprehending how a substance acts in the body [71]. These stages describe the journey of a substance from absorption to elimination. Computational tools have become indispensable for predicting the ADME characteristics of molecules, assessing their ability to cross cellular barriers and interact with essential transporters and enzymes for absorption and excretion, and determining their metabolic stability [59]. In our approach to molecule evaluation, we have chosen to use the SwissADME platform (available online: www.swissadme.ch, accessed on 10 April 2024) [61]. This platform allows us to thoroughly examine the physicochemical attributes of synthetic molecules, their potential as therapeutic agents, and their pharmacokinetic properties, thus providing a comprehensive understanding of their ADME profile [72].

## 4. Conclusions

A series of novel polyheterocyclic molecules, incorporating all three heterocycles [1,4]-benzoxazin-3-one, 1,2,3-triazole, and isoxazoline, were synthesized with high yields. This synthesis involved a double 1,3-dipolar cycloaddition reaction. Initially, a 1,3-dipolar cycloaddition reaction of the “click chemistry” type was conducted in a one-pot process at room temperature using (CuSO_4_$\cdot 5H_{2}O$ and sodium ascorbate) as catalysts. Subsequently, the second cycloaddition reaction was carried out between the allylic part of compound **3** and various oximes at a temperature ranging from −5 to 0 °C. These molecules exhibit potential biological activities, as demonstrated by their testing on α-amylase and α-glucosidase. The study reveals that the majority of the synthesized compounds (**5a**–**5o**) exhibit favorable binding affinities compared with the native ligand acarbose, suggesting potent inhibitory potential against both enzymes. Particularly, compounds **5a** and **5o** demonstrate notable interactions with amino acid residues surrounding the active site of α-amylase, while compounds **5n** and **5e** exhibit strong interactions with the active site of α-glucosidase. Additionally, ADME analyses suggest that most of the synthetic compounds have promising pharmacokinetic profiles for potential drug development. These findings support the potential therapeutic efficacy of the synthesized molecules in combating hyperglycemia by targeting key enzymes involved in carbohydrate metabolism. Further studies could involve in vitro and in vivo experiments to validate the antidiabetic potential of the synthesized compounds. Additionally, structural modifications could be explored to enhance the potency and specificity of these compounds for potential drug development purposes.

## Figures and Tables

**Table 1:** ^1^H NMR and ^13^C NMR spectra exhibit some characteristic signals of the synthesized compounds **5a**–**5o**.

	^1^H NMR (ppm)		^13^C NMR (ppm)	
	CH Triazol	CH_2_ Isoxazoline	C=O	C=N
**5a**	8.02	3.20, 3.50	164.66	157.23
**5b**	8.04	3.16, 3.52	164.64	155.27
**5c**	8.03	3.29, 3.63	164.61	156.20
**5d**	8.02	3.29, 3.57	164.64	156.41
**5e**	8.01	3.20, 3.52	164.66	156.50
**5f**	7.94	2.95, 3.35	164.68	158.64
**5g**	8.00	3.13, 3.45	164.65	149.17
**5h**	8.01	3.05, 3.34	164.68	158.38
**5i**	8.01	3.20, 3.54	164.65	149.17
**5j**	8.00	3.14, 3.46	164.86	151.18
**5k**	8.01	3.15, 3.49	164.62	156.60
**5l**	8.01	3.12, 3.44	164.66	156.93
**5m**	8.01	3.15, 3.49	164.95	156.63
**5n**	8.03	3.18, 3.53	164.68	157.22
**5o**	8.01	3.20, 3.52	167.33	156.43

**Table 2:** H-bonds, binding energy, and interacting amino acids of phytocompounds found in the synthetic molecules **5a**–**5o** target protein.

Compounds	α-Amylase Protein (PDB ID: 1SMD)	
	Affinity (kcal/mol)	H-Bonding
Acarbose 1	−7.8	Tyr A:2, Gln A:7, Ser A:289, Ser A:289, Asp A:402, Gly A:334
**5a**	−9.2 *	-
**5b**	−8.7 *	-
**5c**	−8.2 *	Gln A:63, Arg A:195, His A:299
**5d**	−9.0 *	His A:201
**5e**	−9 *	-
**5f**	−8.4 *	-
**5g**	−8.3 *	His A:201
**5h**	−8.2 *	-
**5i**	−8.6 *	-
**5j**	−7.9 *	-
**5k**	−8.2 *	-
**5l**	−8.8 *	-
**5m**	−8.8 *	Lys A:200
**5n**	−7.7	Asp A:63
**5o**	−9.1 *	His A:202

1 Acarbose, a native ligand of α-amylase; * The potent ligands in comparison with the native ligand.

**Table 3:** H-bonds, binding energy, and interacting amino acids of phytocompounds found in the synthetic molecules **5a**–**5o** target protein.

Compounds	α-Glucosidase Protein (PDB ID: 5NN5)	
	Affinity (kcal/mol)	H-Bonding
Acarbose 1	−7.2	Asp A:356, Met A:363, Glu A: 866, Arg A:608
**5a**	−9.5 *	Tyr A:360, Met A:363, Arg A:608, Glu A:866
**5b**	−9.5 *	Tyr A:360, Met A:363, Arg A:608, Glu A:866
**5c**	−9.5 *	Tyr A:360, Met A:363, Arg A:608, Glu A:866
**5d**	−8.9 *	Tyr A:360, Glu A:866
**5e**	−9.9 *	Tyr A:360, Met A:363, His A: 584, Arg A:608, Glu A:866
**5f**	−9 *	Arg A:608, Glu A:866
**5g**	−9.1 *	Tyr A:360, Met A:363, Arg A:608, Glu A:866
**5h**	−9.1 *	Tyr A:360, Met A:363, Arg A:608, Glu A:866
**5i**	−9.5 *	Tyr A:360, Met A:363, Arg A:608, Glu A:866
**5j**	−8.8 *	Tyr A: 360, Glu A:866
**5k**	−9.5 *	-
**5l**	−8.7 *	Tyr A:360, Arg A: 866
**5m**	−9.1 *	Tyr A:360, Arg A: 608
**5n**	−9.6 *	Tyr A:360, Met A:363, Arg A:608, Glu A:866
**5o**	−9.4 *	Tyr A:360, Arg A:608, Glu A:866

1 Acarbose, a native ligand of α-amylase; * The potent ligands in comparison with the native ligand.

**Table 4:** Evaluation of the pharmacokinetic properties (ADME) of the Synthetic Compounds.

	Physicochemical Properties					Lipophilicity		Druglikeness		Pharmacokinetics		
Compounds	MW g/mol	HBA	HBD	TPSA Å^2^	Rotatable Bonds	M logP	W logP	Lipinski’s	Verber’s	GI Absorption	BBB Permeation	CYP1A2 Inhibitor
**5a**	403.4	6	0	81.8	5	1.9	1.7	0	0	High	No	Yes
**5b**	434.4	8	0	131.2	6	1.8	1.2	0	0	High	No	No
**5c**	434.4	8	0	131.2	6	1.8	1.2	0	0	High	No	No
**5d**	434.4	8	0	131.2	6	1.8	1.2	0	0	High	No	No
**5e**	423.8	6	0	81.8	5	2.2	2.1	0	0	High	No	Yes
**5f**	403.4	6	0	81.8	6	1.9	1.6	0	0	High	No	No
**5g**	379.3	7	0	94.9	5	0.5	1.0	0	0	High	No	Yes
**5h**	415.4	6	0	81.8	6	2.1	2.0	0	0	High	No	No
**5i**	390.4	7	0	94.7	5	0.7	0.8	0	0	High	No	Yes
**5j**	392.4	6	0	86.7	5	0.8	0.8	0	0	High	No	No
**5k**	468.3	6	0	81.8	5	2.3	2.2	0	0	High	No	Yes
**5l**	432.4	6	0	85.0	6	1.6	1.5	0	0	High	No	No
**5m**	419.4	7	0	97.0	6	1.4	1.4	0	0	High	No	Yes
**5n**	419.4	7	0	91.07	6	1.47	1.49	0	0	High	No	No
**5o**	407.4	7	0	81.8	5	2.4	2.0	0	0	High	No	Yes

**MW:** molecular weight; **HBD**: Hydrogen-Bond Donors; **HBA**: Hydrogen-Bond Acceptors; **WLogP**: Lipophilicity; **MLogP**: Octanol/water partition coefficient; **TPSA**: Topological polar surface area; **BBB**: Blood-Brain Barrier; **GI**: Gastrointestinal.

## Data Availability

The article contains the original contributions that were made in the study. For additional queries, please contact the corresponding author.

## Supplementary Materials

The following supporting information can be downloaded at: https://www.mdpi.com/article/10.3390/molecules29133086/s1, Figures S1–S56: Spectra of compounds **3**, **5a**, **5b**, **5c**, **5d**, **5e**, **5f**, **5g**, **5h**, **5i**, **5j**, **5k**, **5l**, **5m**, **5n**, **5o**.
