# Supplementary material for: Synthesis and In Silico Analysis of New Polyheterocyclic Molecules Derived from [1,4]-Benzoxazin-3-one and Their Inhibitory Effect against Pancreatic α-Amylase and Intestinal α-Glucosidase
Source: Molecules. 2024 Jun 28;29(13):3086. doi: 10.3390/molecules29133086 (PMC11243342; doi:10.3390/molecules29133086)
Supplement: Supplementary file 1 [file molecules-29-03086-s001.zip › molecules-3054530-supplementary.pdf]

Synthesis and In Silico Analysis of New Polyheterocyclic Molecules Derived from [1,4]-Benzoxazin-3-one and Their Inhibitory Effect against Pancreatic α-Amylase and Intestinal α-Glucosidase

Mohamed Ellouz [1,*], Aziz Ihammi [1,*], Abdellah Baraich [2], Ayoub Farihi [3,4], Darifa Addichi [1], Saliha Loughmari [1], Nada Kheira Sebbar [5], Mohamed Bouhrim [6,7], Ramzi A. Mothana [8], Omar M. Noman [8], Bruno Eto [7], Fatiha Chigr [6] and Mohammed Chigr [1]

[1] Laboratory of Molecular Chemistry, Materials and Catalysis (LCMMC), Faculty of Sciences and Technology, Sultan Moulay Slimane University, P.O. Box 523, Beni-Mellal 23000, Morocco; darifaadd@gmail.com (D.A.); salihaloughmari@gmail.com (S.L.); chigrm@gmail.com (M.C.)
[2] Laboratory of Bioresources, Biotechnology, Ethnopharmacology and Health, Faculty of Sciences, Mohammed First University, Boulevard Mohamed VI, P.O Box 717, Oujda 60000, Morocco; abdellah.baraich@ump.ac.ma
[3] Laboratory of Biology and Health, Faculty of Sciences, Ibn Tofail University, Kenitra 14000, Morocco; ayoub.farihi@uit.ac.ma
[4] Oriental Center for Water and Environmental Sciences and Technologies (COSTE), Mohammed Premier University, Oujda 60000, Morocco
[5] Laboratory of Organic and Physical Chemistry, Applied Bioorganic Chemistry Team, Faculty of Sciences, Ibnou Zohr University, Agadir 80000, Morocco; n.sebbar@uiz.ac.ma
[6] Biological Engineering Laboratory, Faculty of Sciences and Techniques, Sultan Moulay Slimane University, Beni Mellal 23000, Morocco; mohamed.bouhrim@gmail.com (M.B.); f.chigr@usms.ma (F.C.)
[7] Laboratoires TBC, Laboratory of Pharmacology, Pharmacokinetics, and Clinical Pharmacy, Faculty of Pharmaceutical and Biological Sciences, P.O Box 83, F-59000 Lille, France; bruno.eto@univ-lille.fr
[8] Department of Pharmacognosy, College of Pharmacy, King Saud University, P.O. Box 2457, Riyadh 11451, Saudi Arabia; rmothana@ksu.edu.sa (R.A.M.); onoman@ksu.edu.sa (O.M.N.)
* Correspondence: ellouz.chimie@gmail.com (M.E.); azizihammi@gmail.com (A.I.)

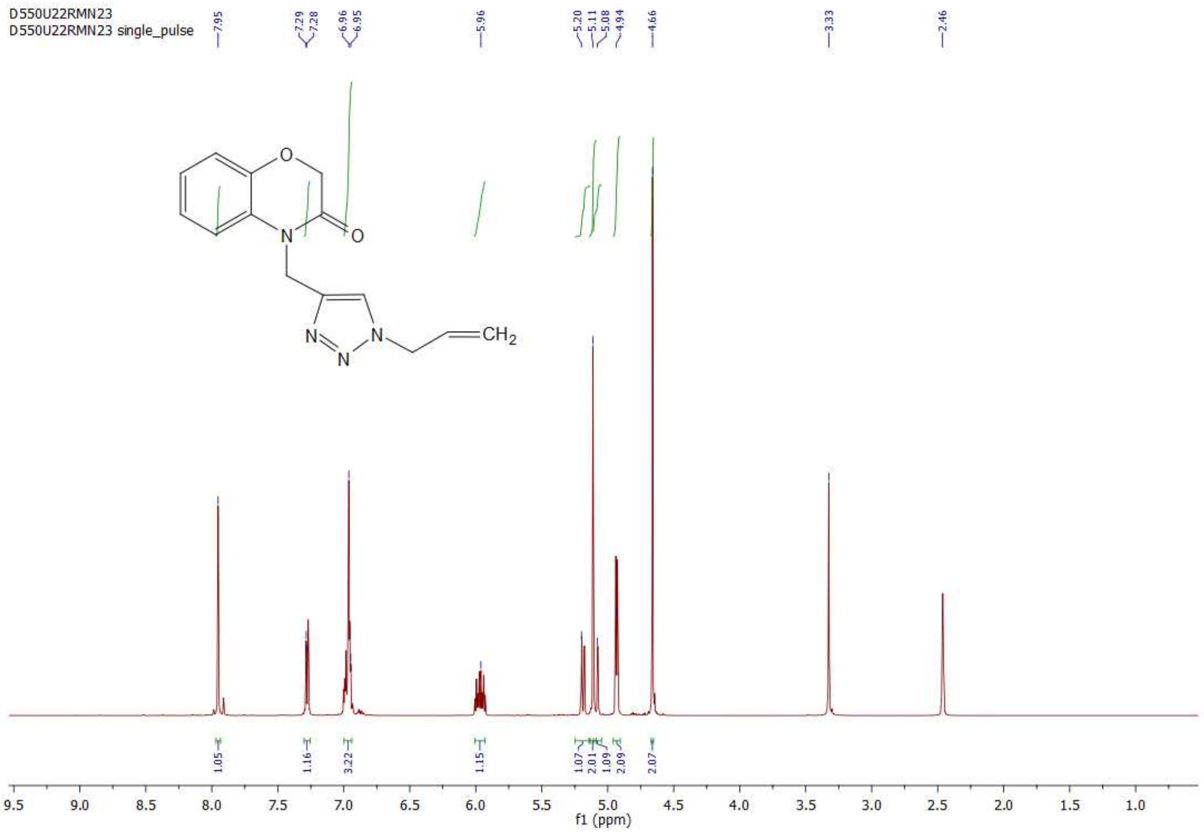

Figure S1**: ^{1}H NMR spectrum of the compound **3

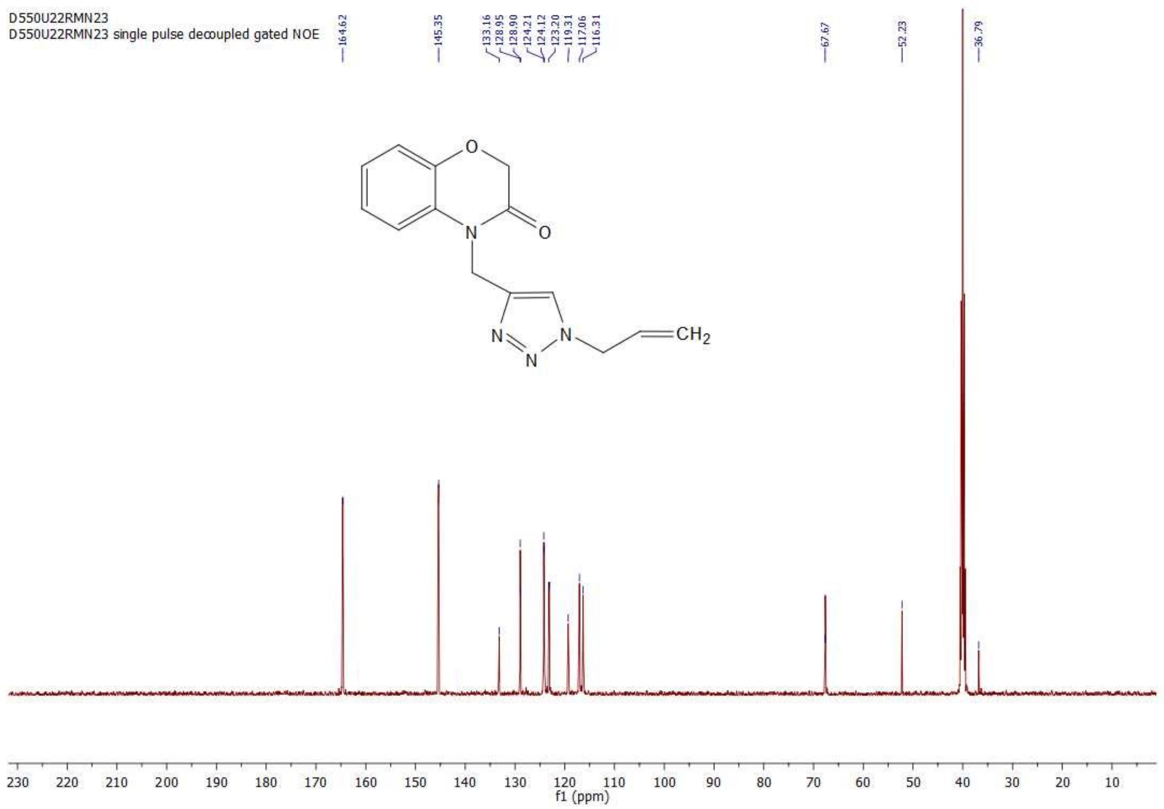

Figure S2: *^{13}C NMR spectrum of the compound **3***

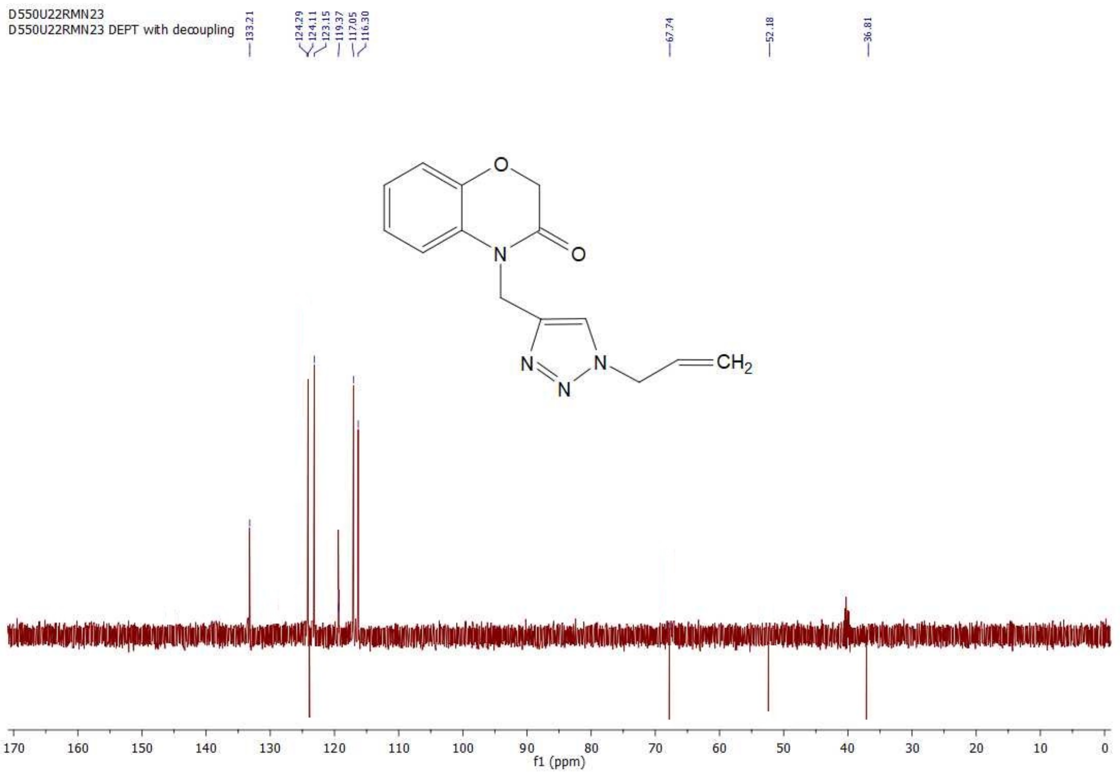

Figure S3: *DEPT-135 spectrum of the compound **3***

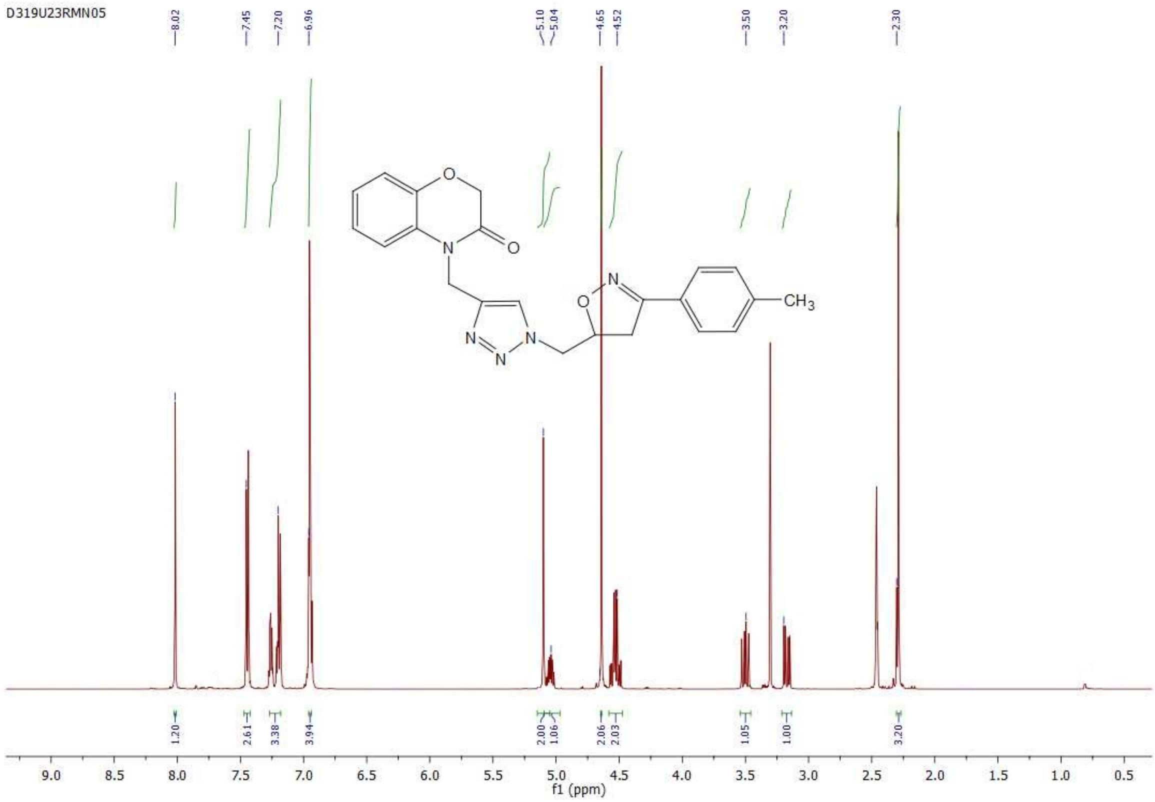

Figure S4: *^{1}H NMR spectrum of the compound* ***5a***

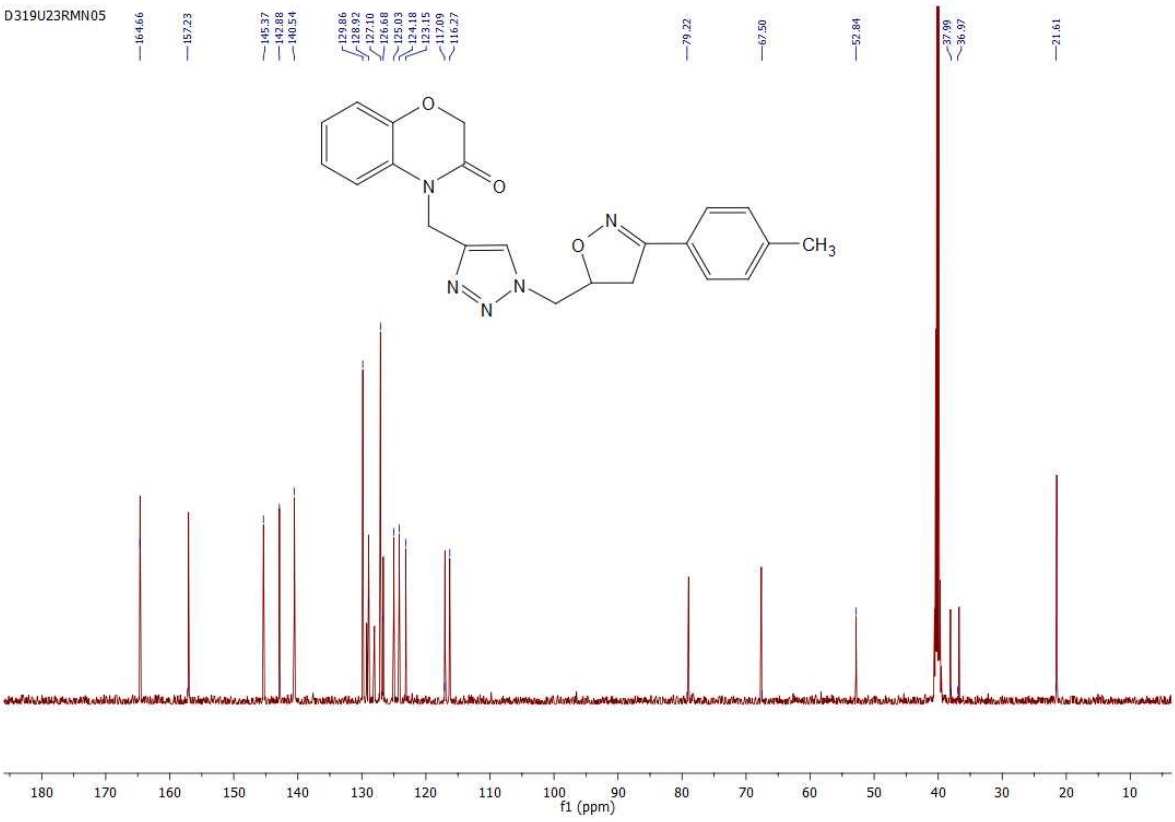

Figure S5: *^{13}C NMR spectrum of the compound* ***5a***

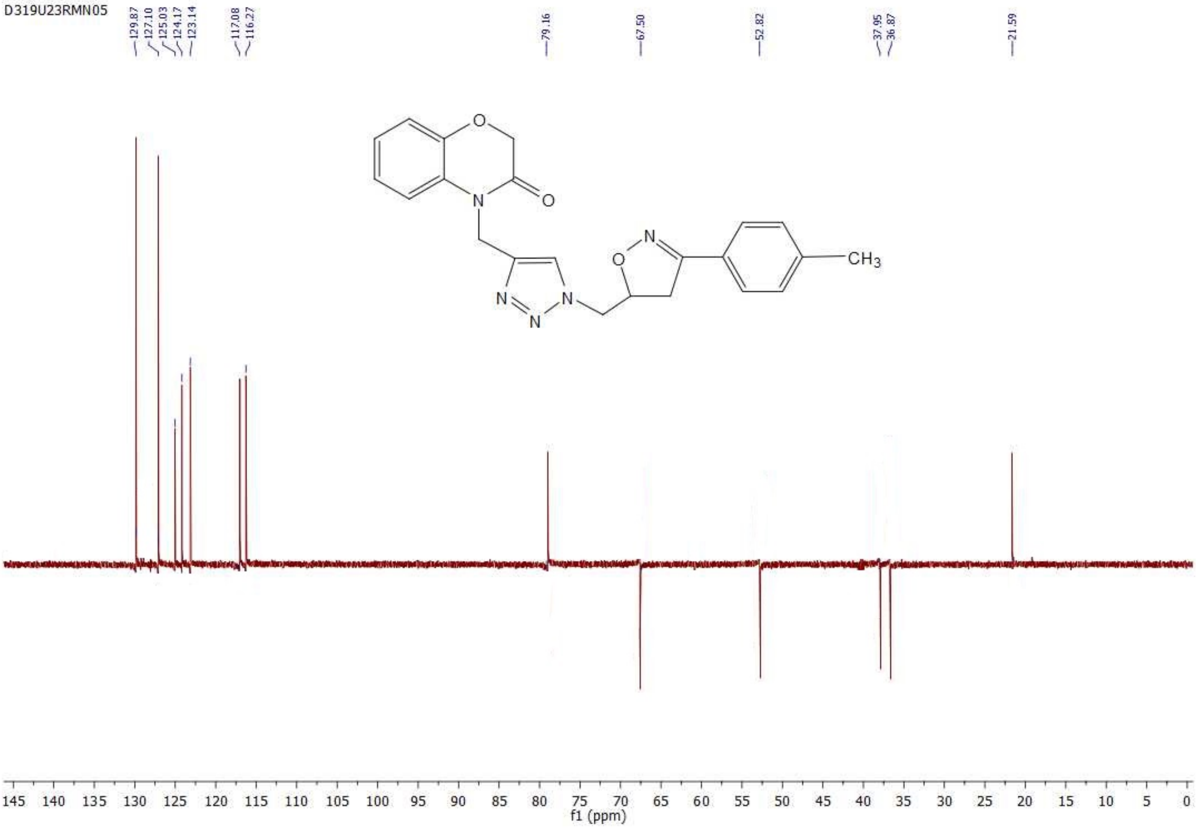

Figure S6: *DEPT-135 spectrum of the compound* ***5a***

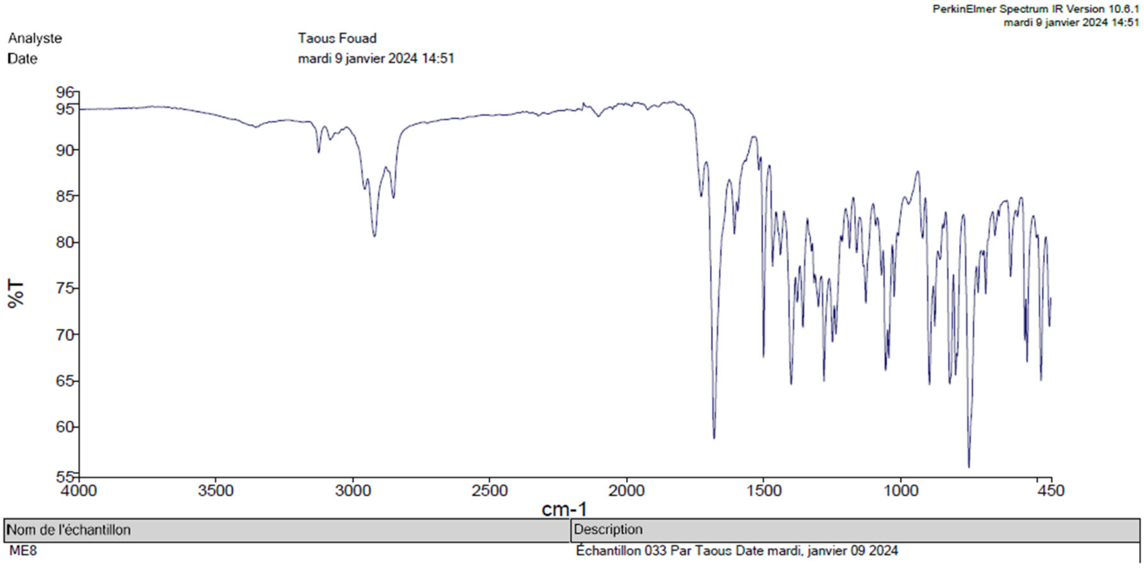

Figure S7: *IR spectrum of the compound* ***5a***

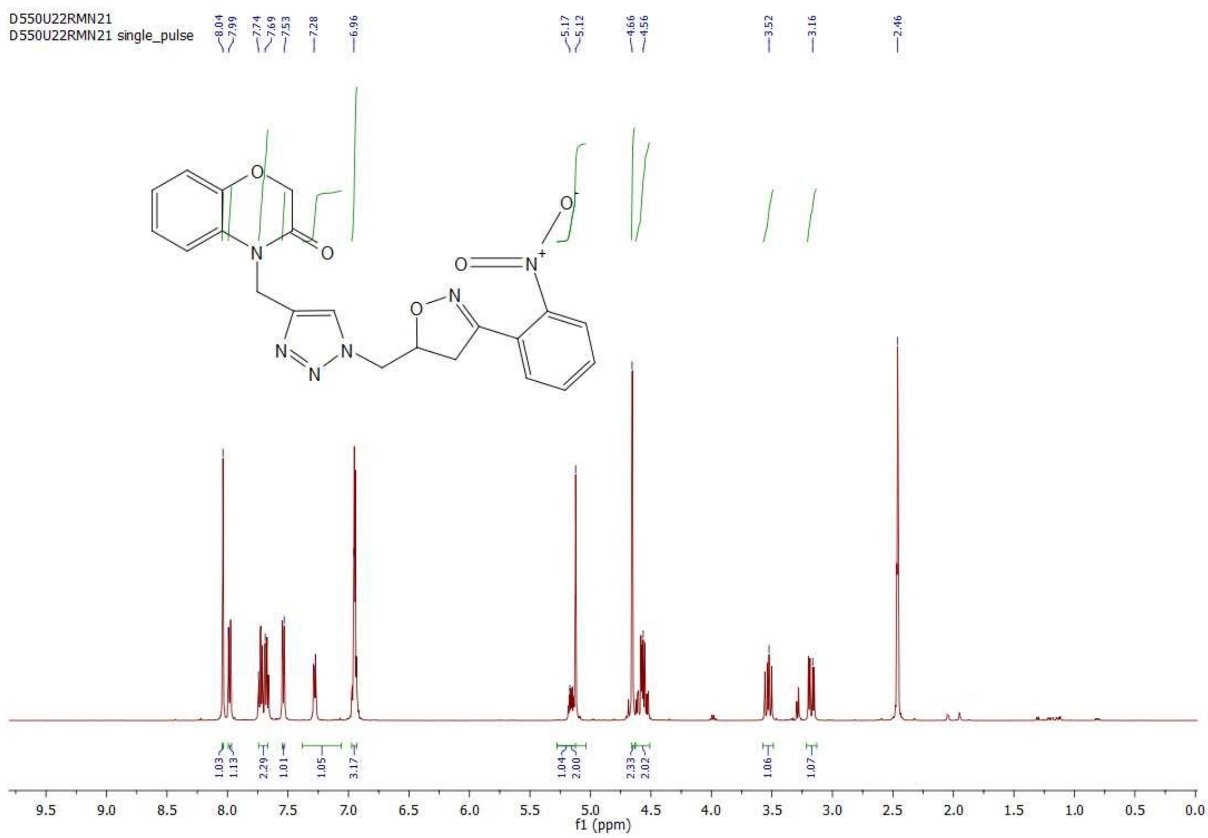

Figure S8: *^{1}H NMR spectrum of the compound* ***5b***

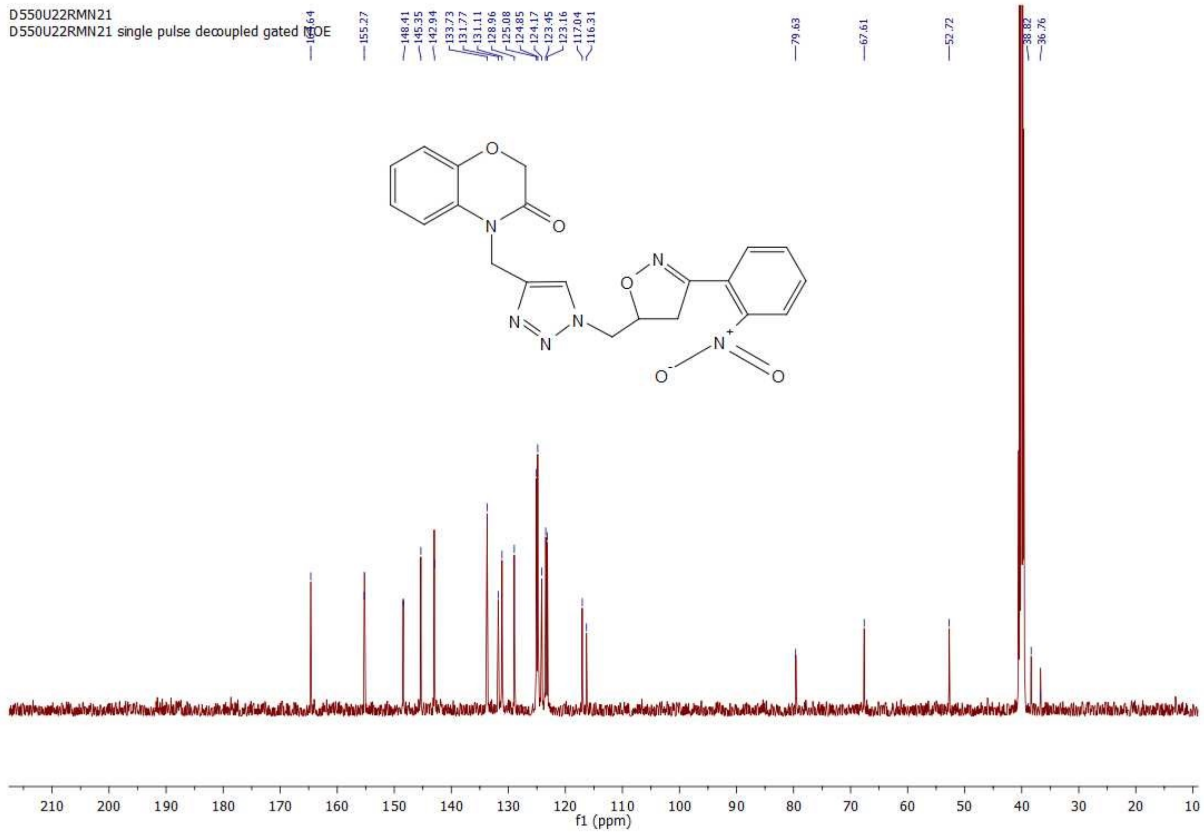

Figure S9: *^{13}C NMR spectrum of the compound* ***5b***

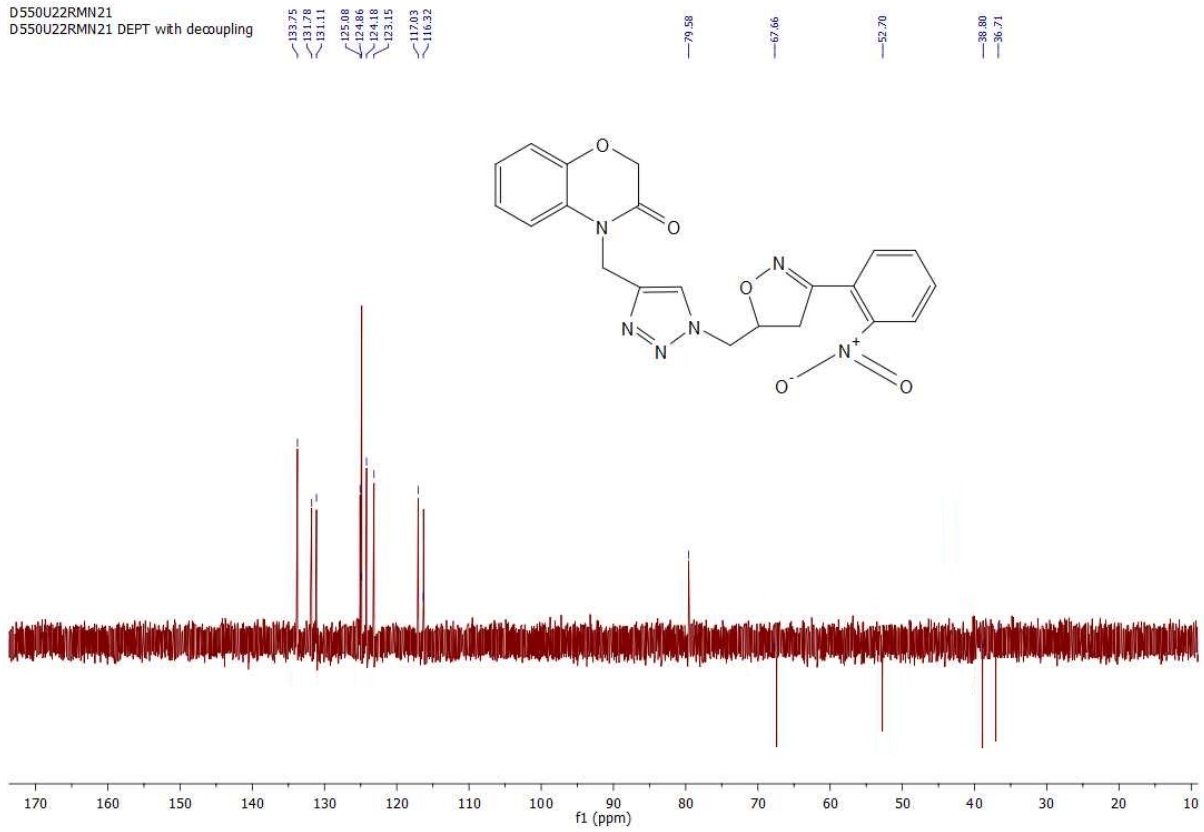

Figure S10: *DEPT-135 spectrum of the compound* ***5b***

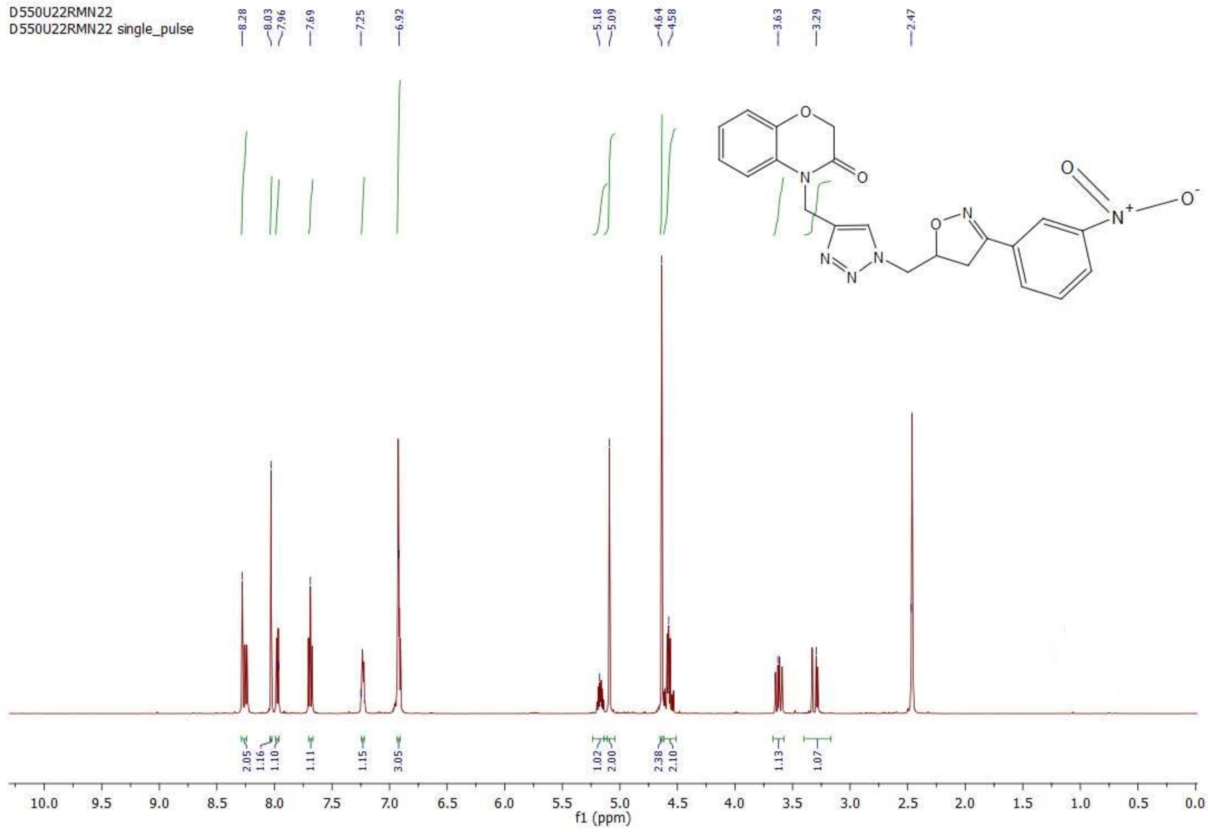

Figure S11: *^{1}H NMR spectrum of the compound* ***5c***

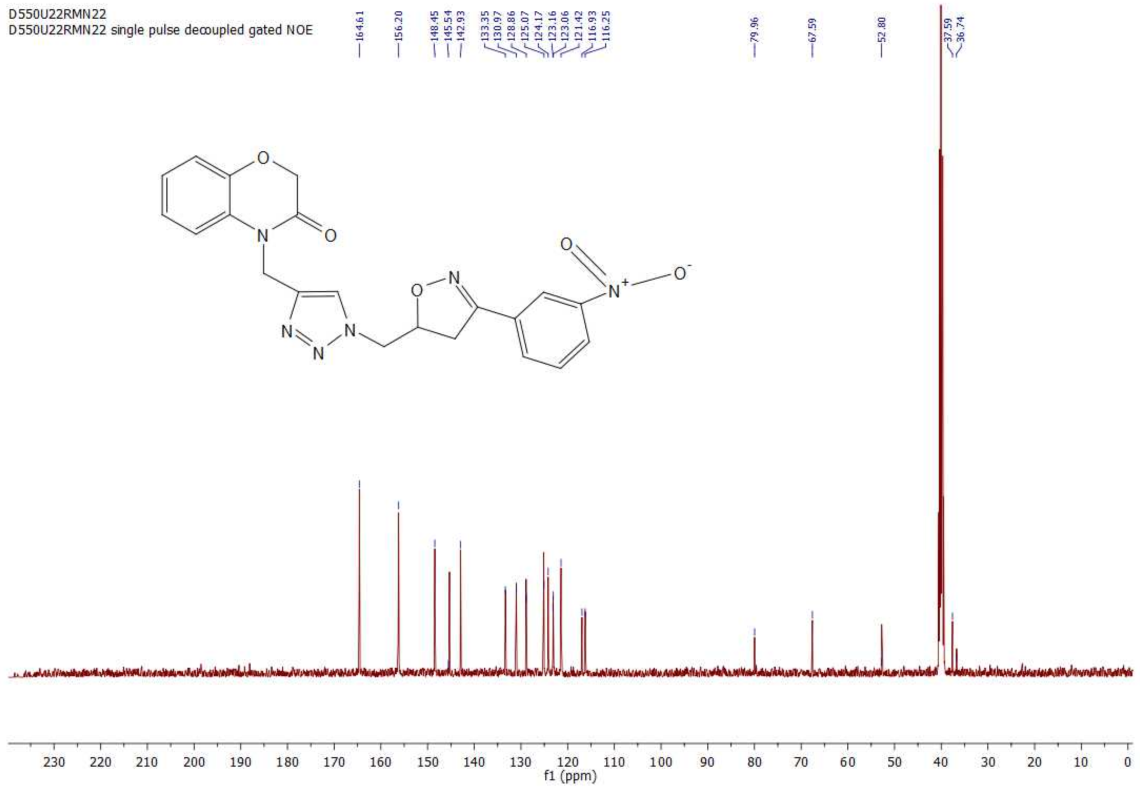

Figure S12: *^{13}C NMR spectrum of the compound* ***5c***

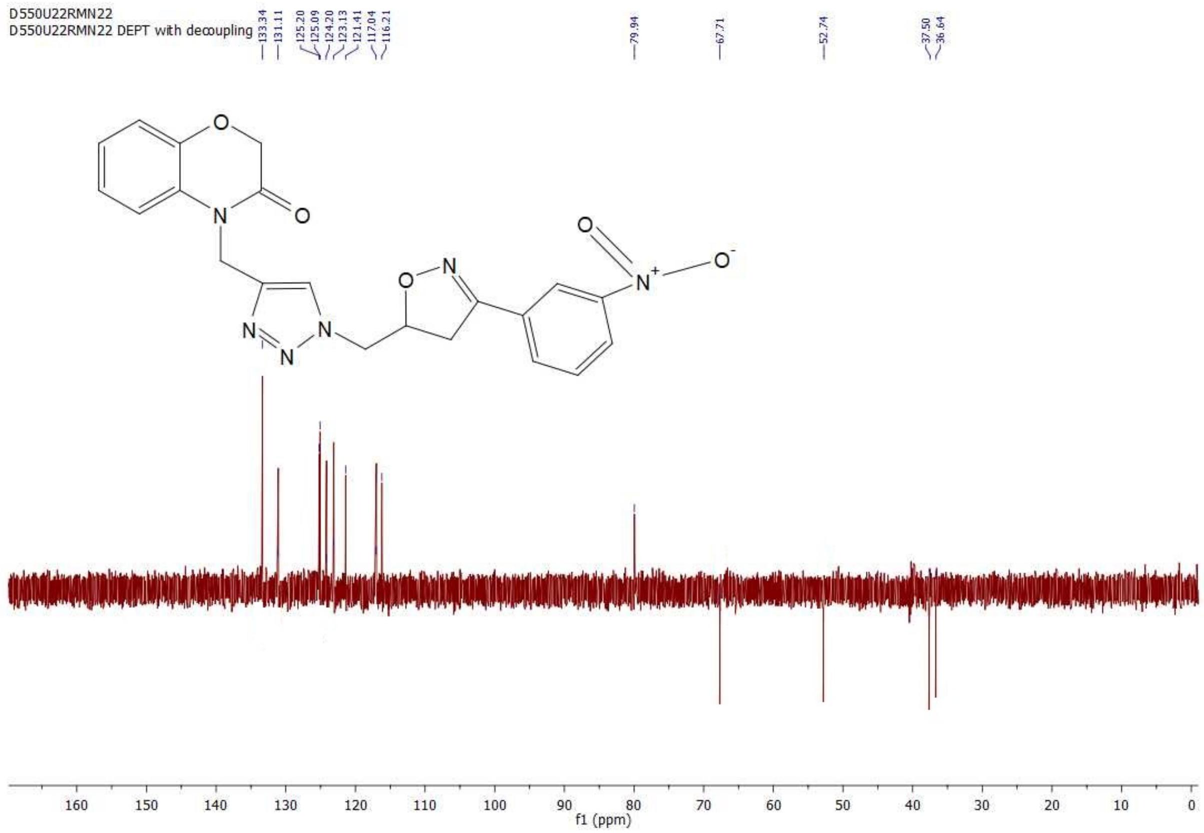

Figure S13*: DEPT-135 spectrum of the compound* ***5c***

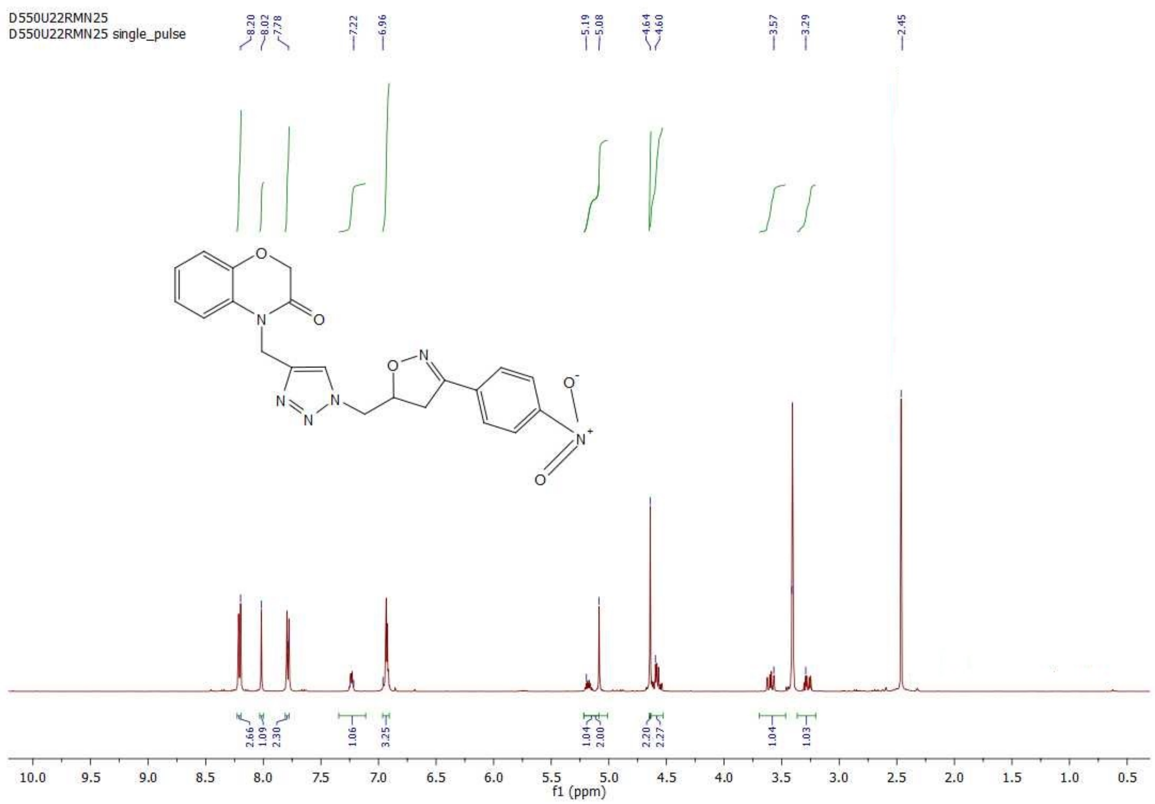

Figure S14: *^{1}H NMR spectrum of the compound* ***5d***

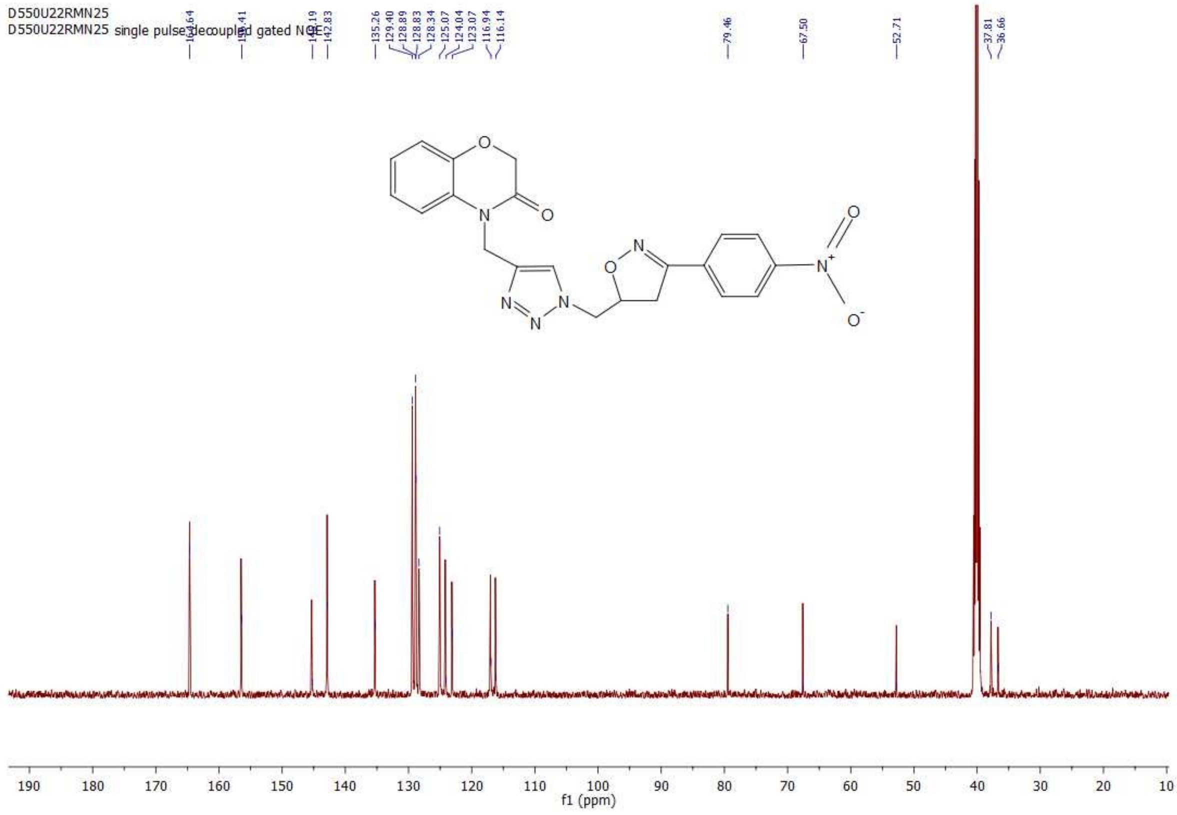

Figure S15: *^{13}C NMR spectrum of the compound* ***5d***

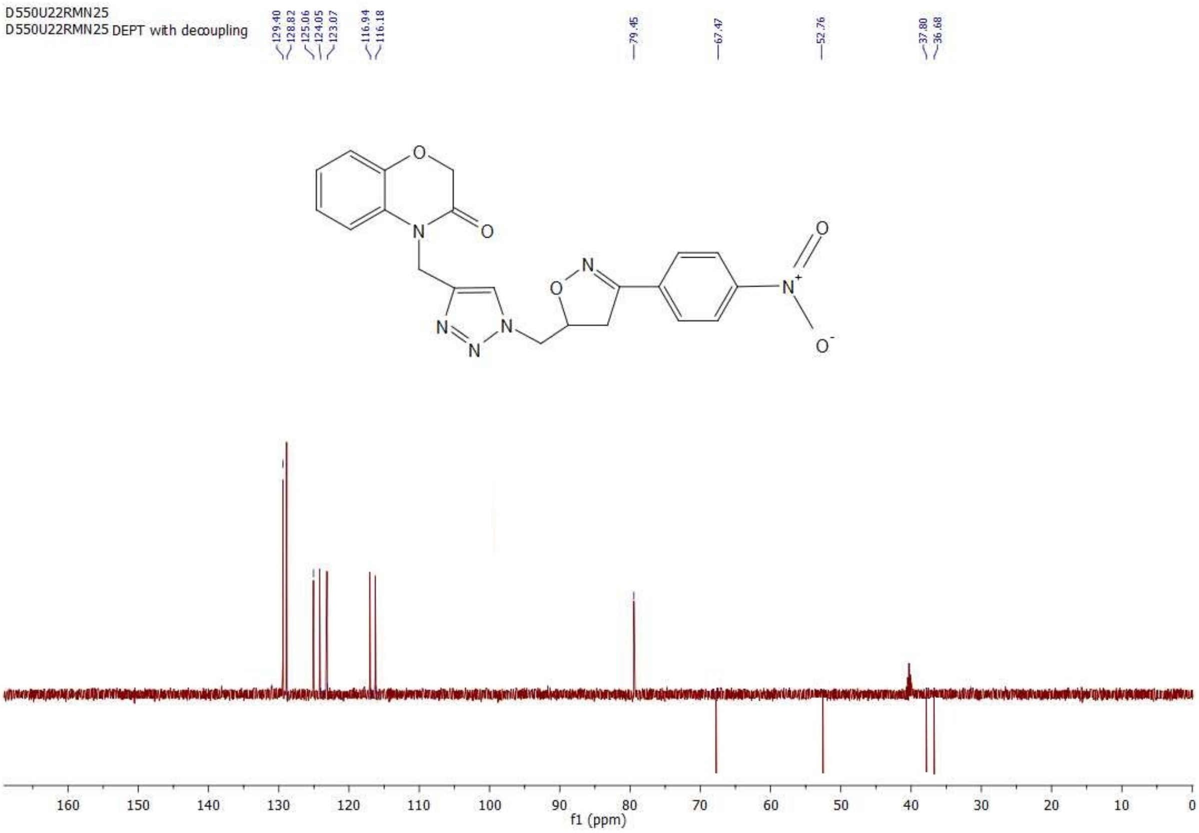

Figure S16*: DEPT-135 spectrum of the compound* ***5d***

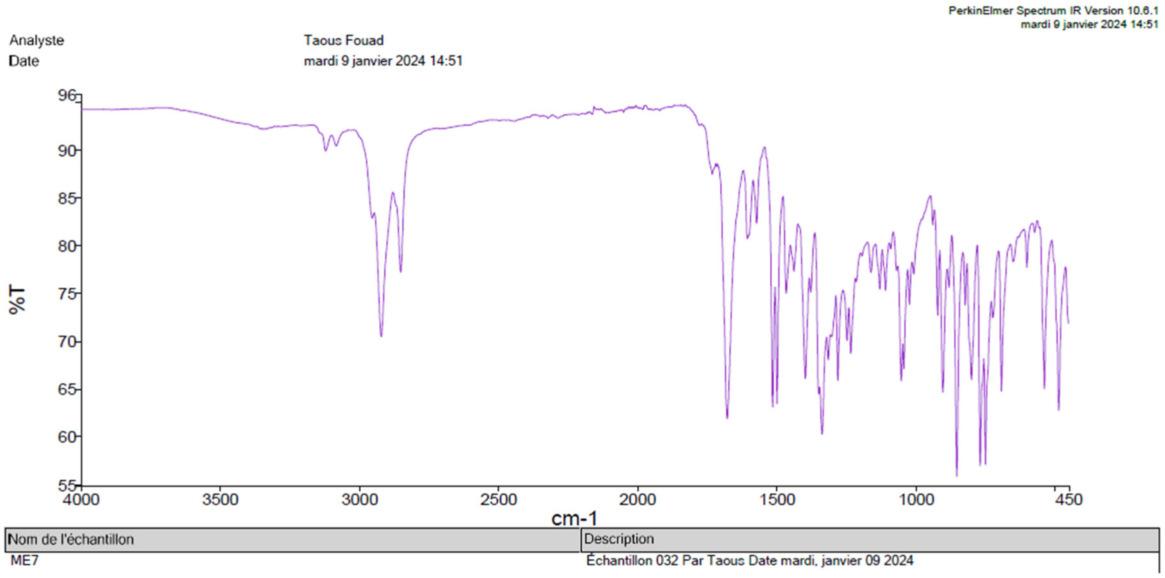

Figure S17*: IR spectrum of the compound* ***5d***

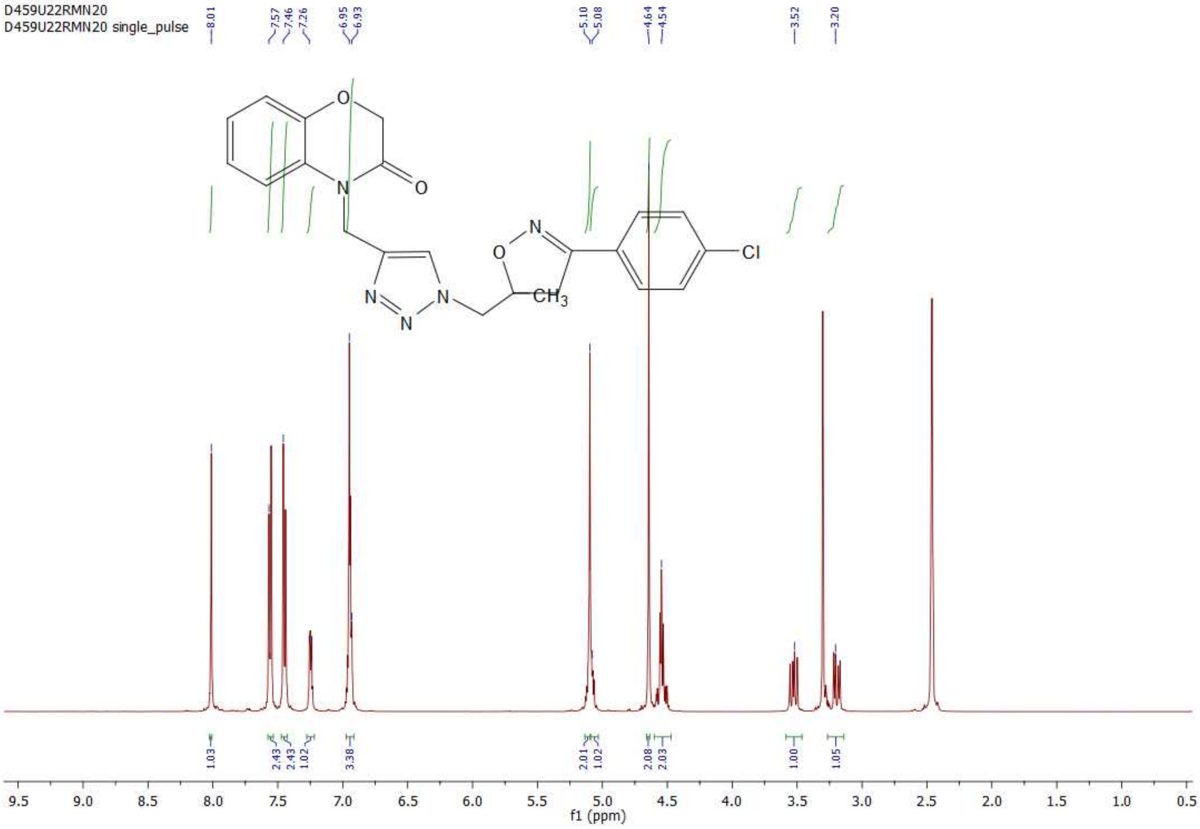

Figure S18: *^{1}H NMR spectrum of the compound* ***5e***

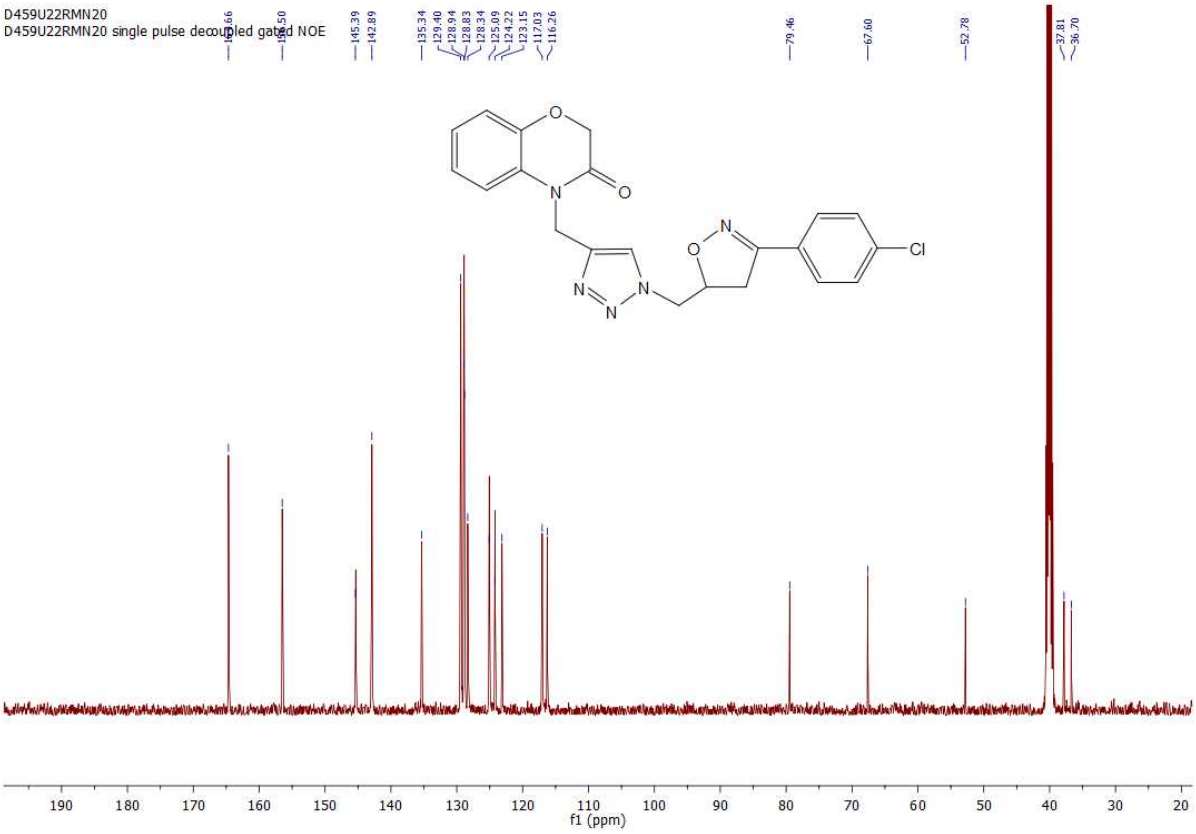

Figure S19: *^{13}C NMR spectrum of the compound* ***5e***

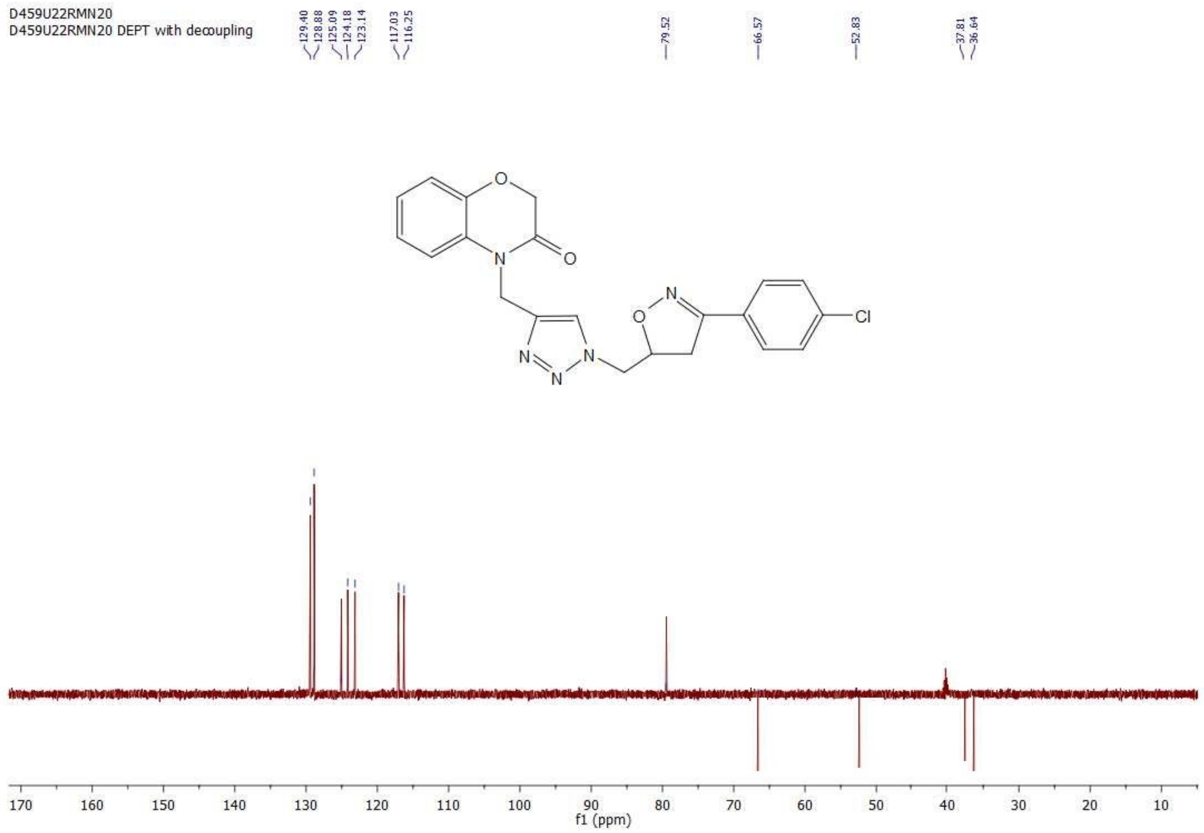

Figure S20*: DEPT-135 spectrum of the compound* ***5e***

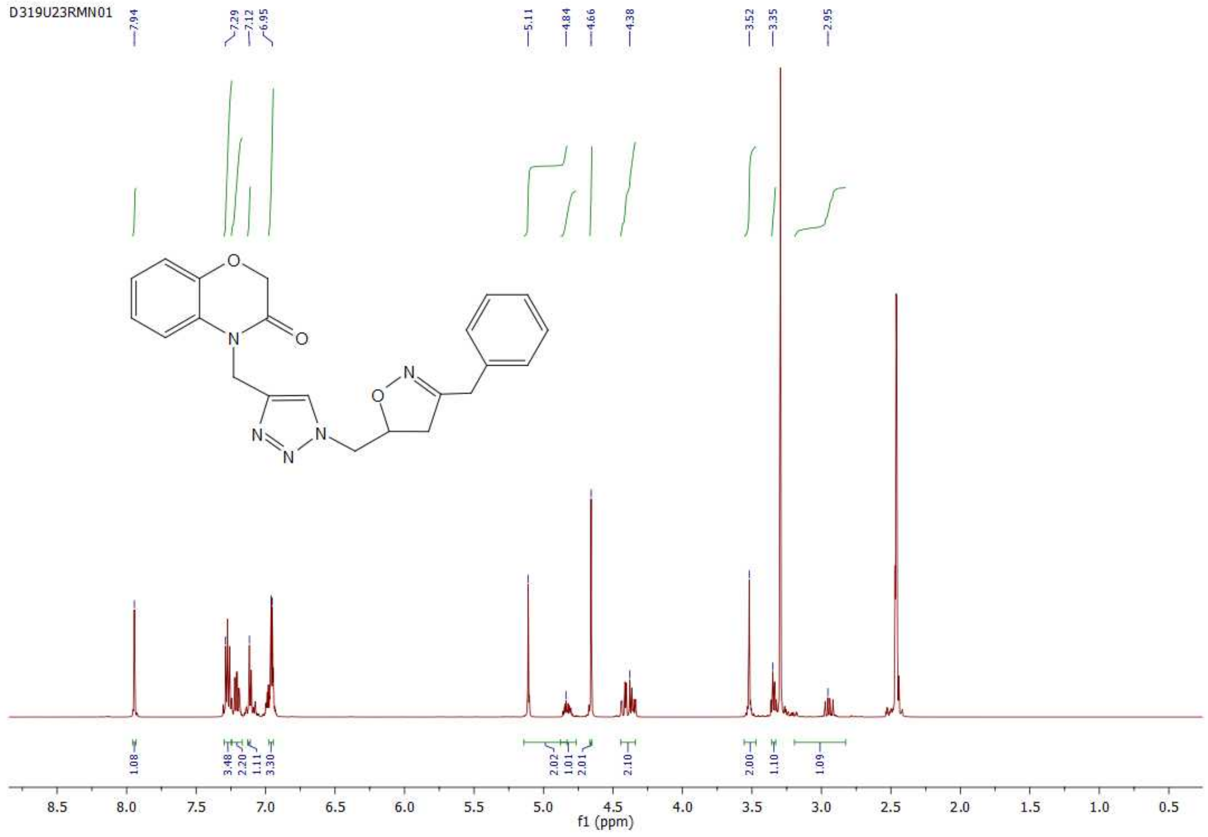

Figure S21*: ^{1}H NMR spectrum of the compound* ***5f***

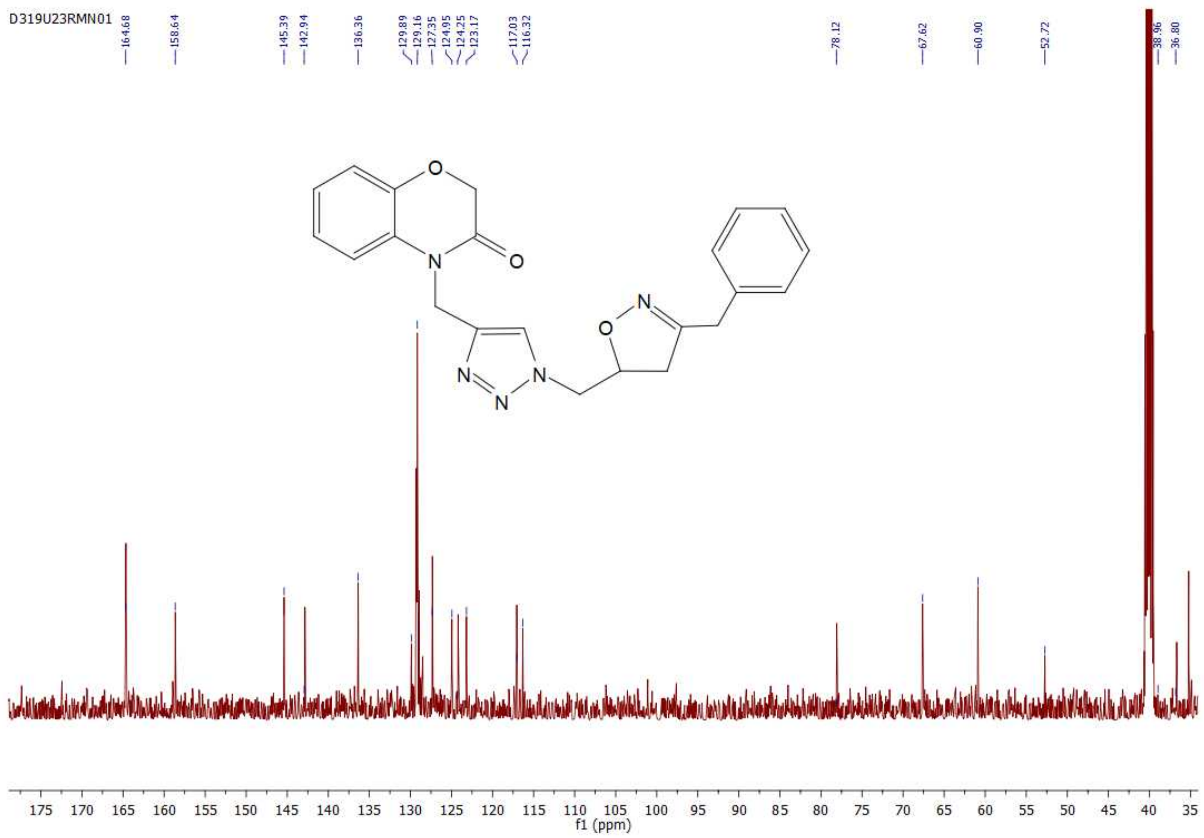

Figure S22: *^{13}C NMR spectrum of the compound* ***5f***

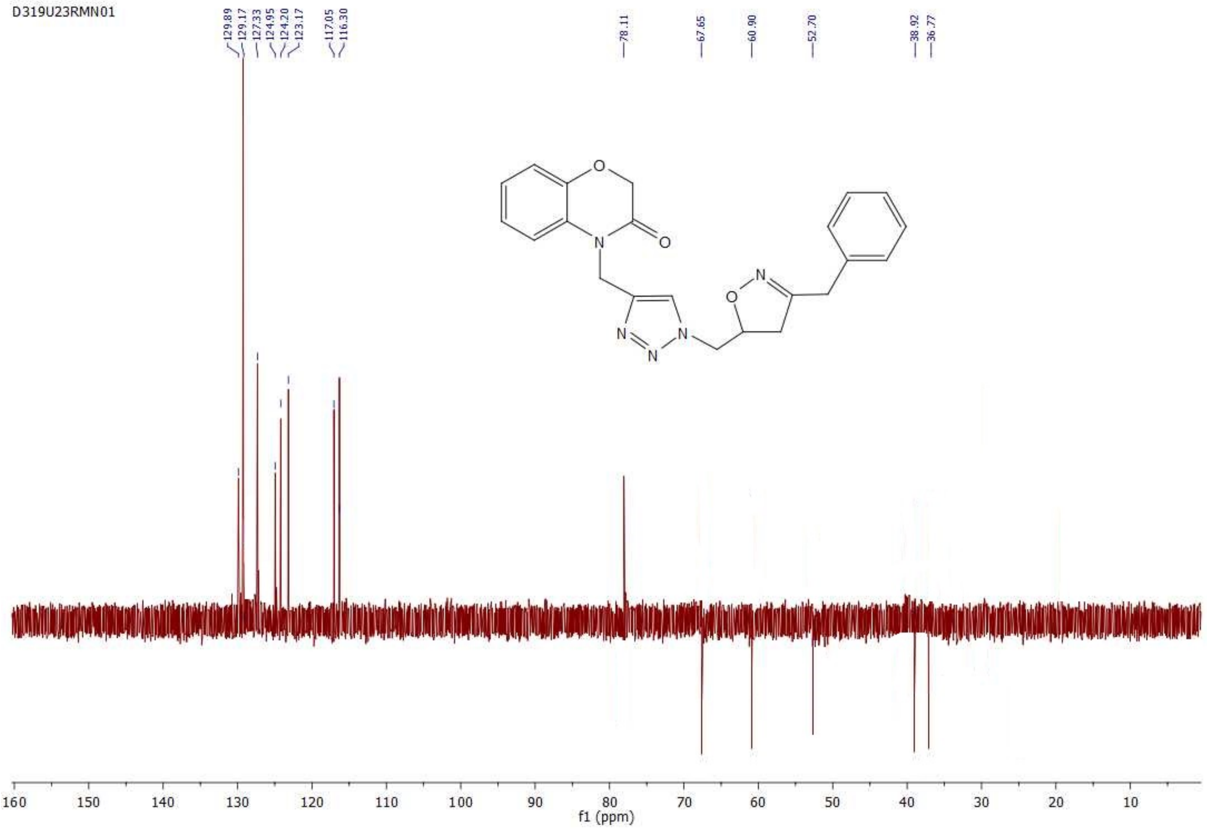

Figure S23: *DEPT-135 spectrum of the compound* ***5f***

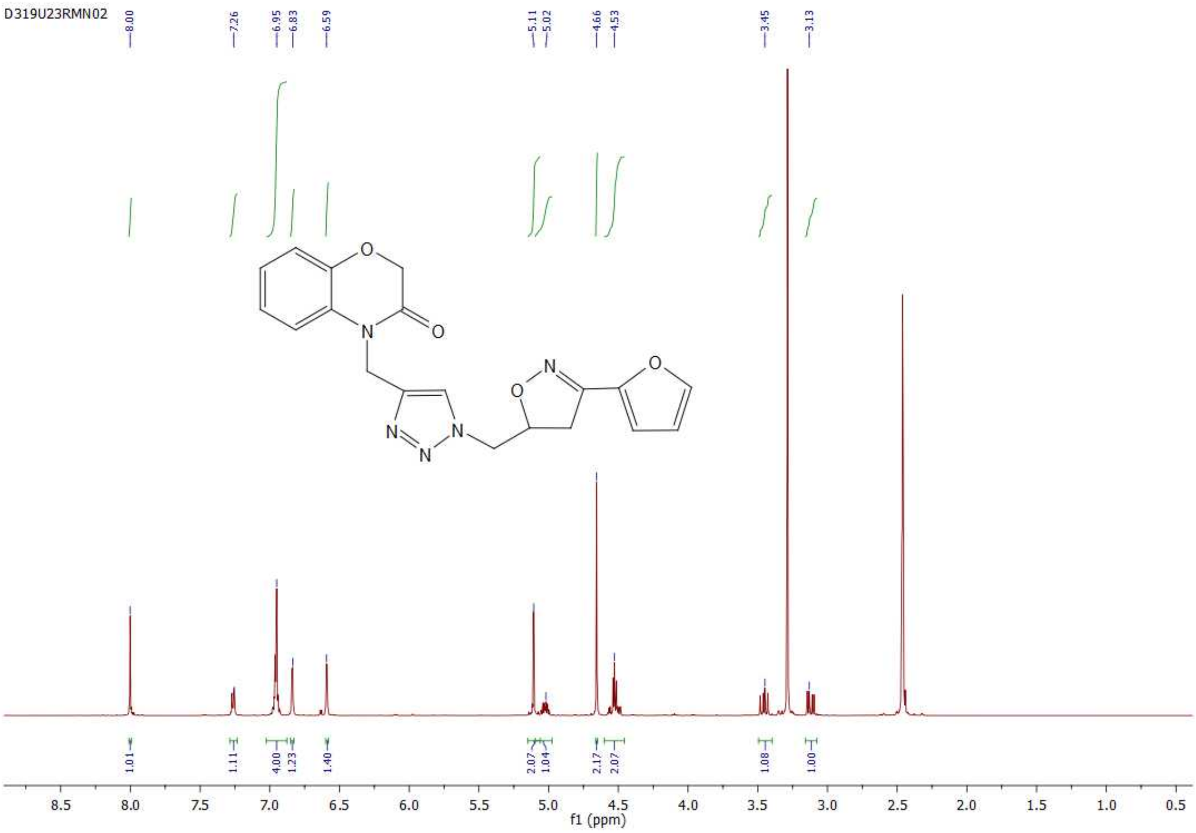

Figure S24: *^{1}H NMR spectrum of the compound* ***5g***

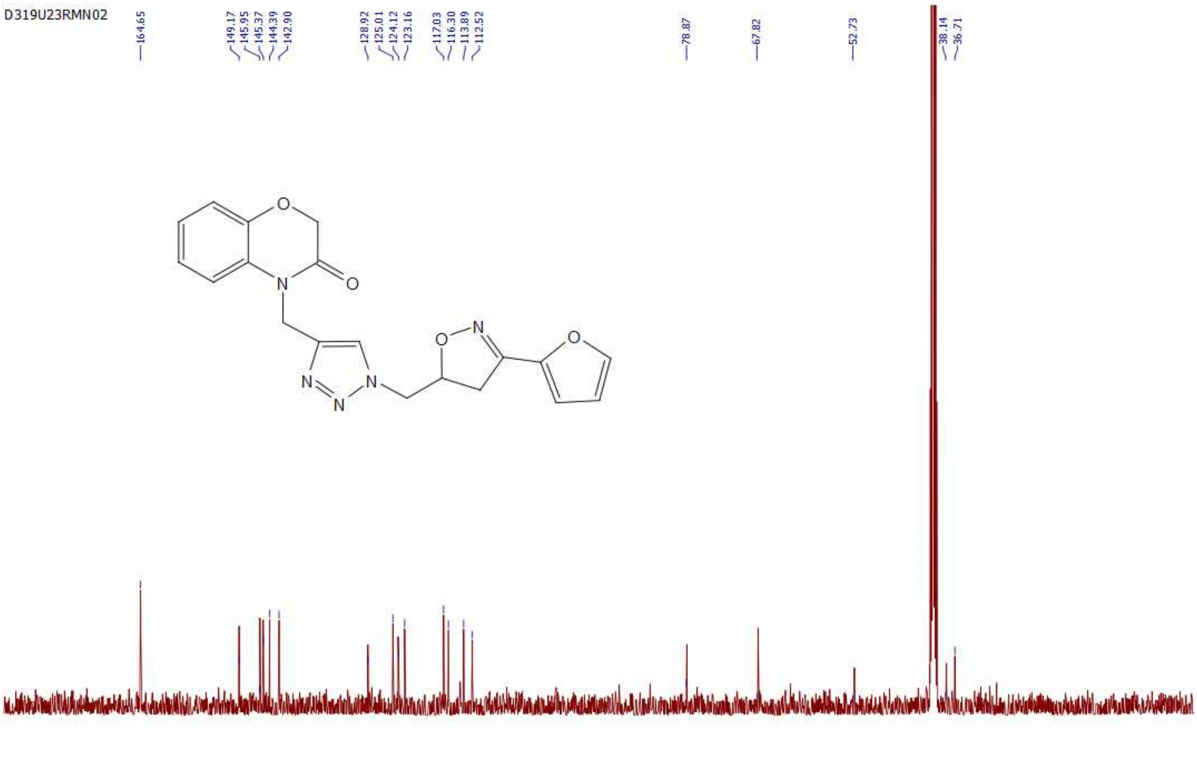

Figure S25: *^{13}C NMR spectrum of the compound* ***5g***

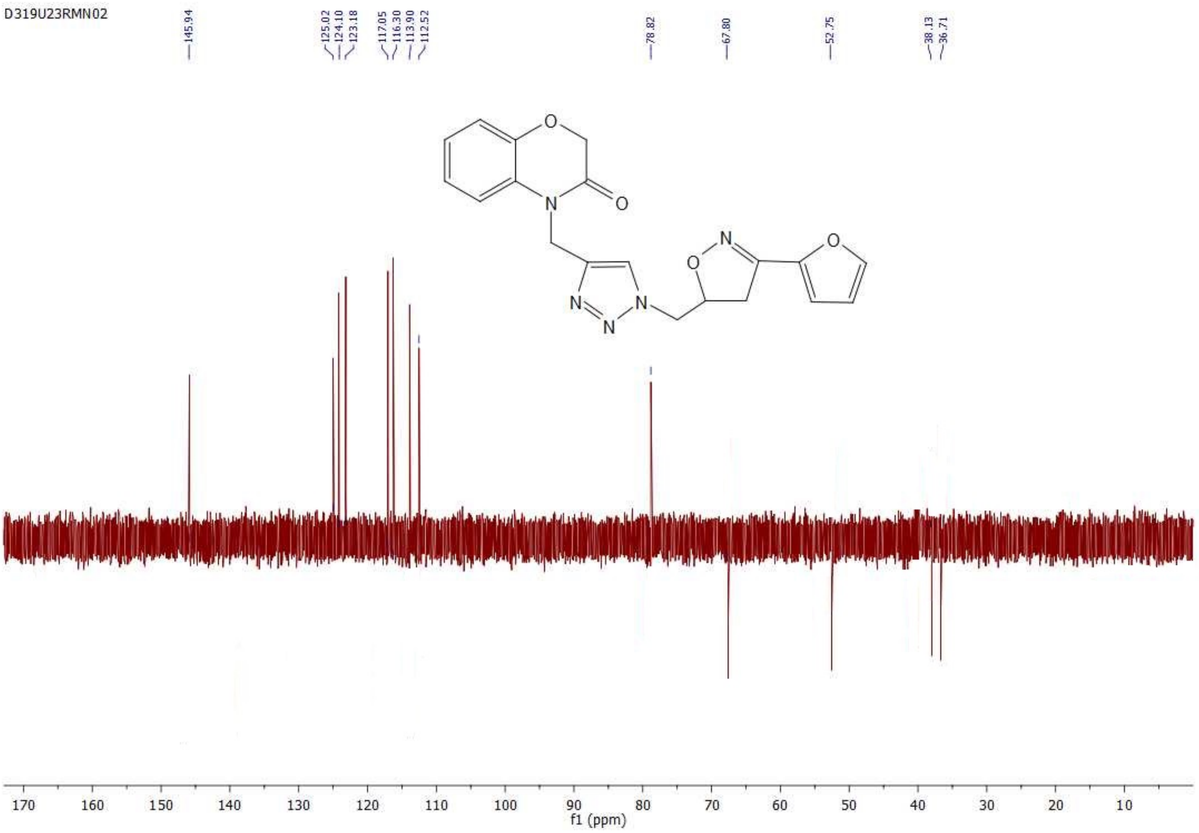

Figure S26: *DEPT-135 spectrum of the compound* ***5g***

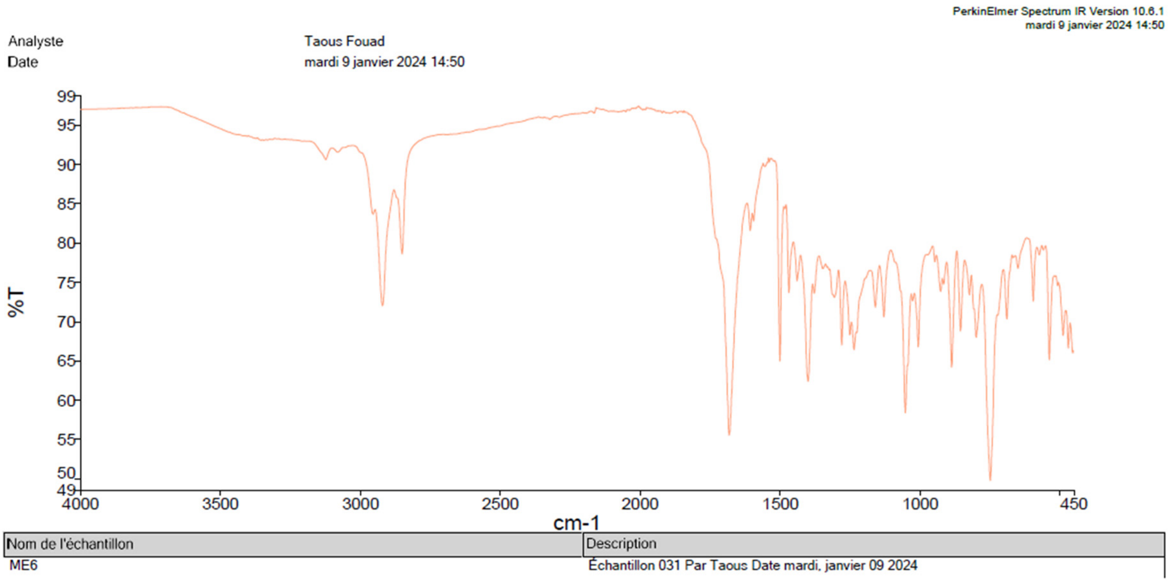

Figure S27: *IR spectrum of the compound* ***5g***

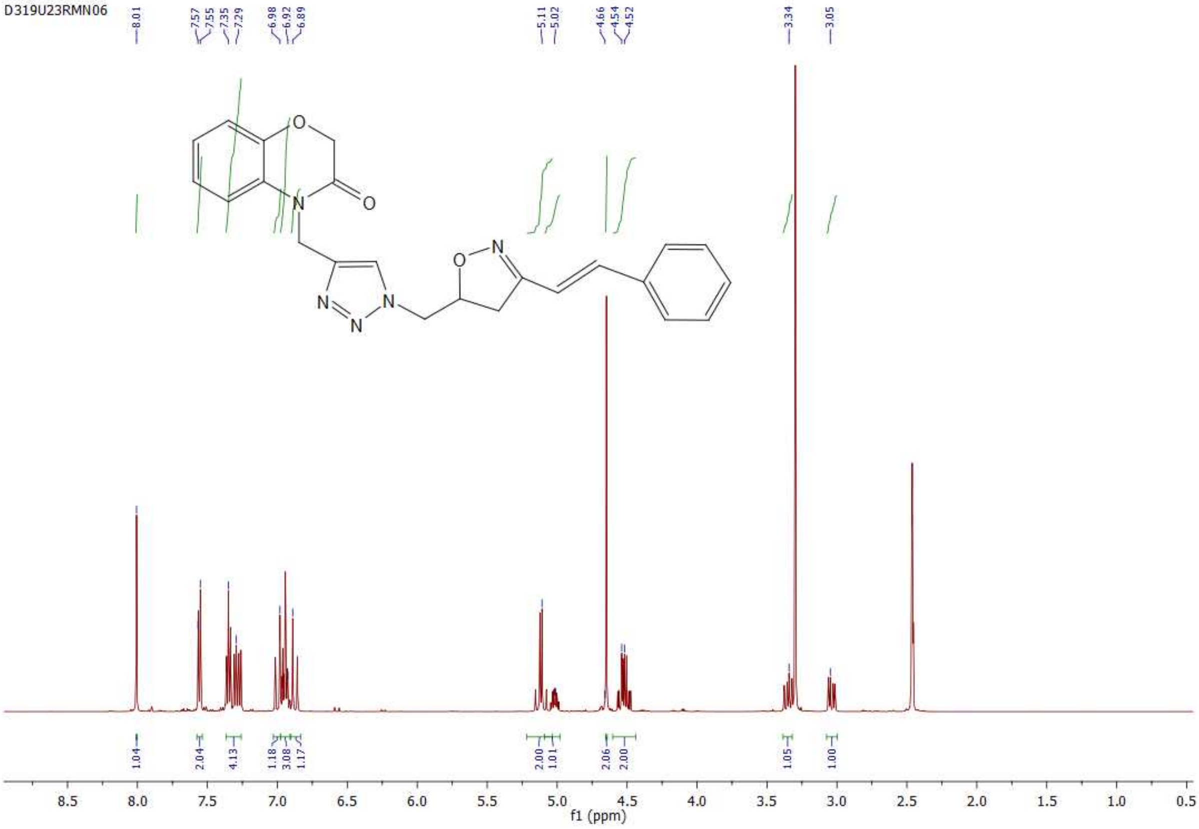

Figure S28: *^{1}H NMR spectrum of the compound* ***5h***

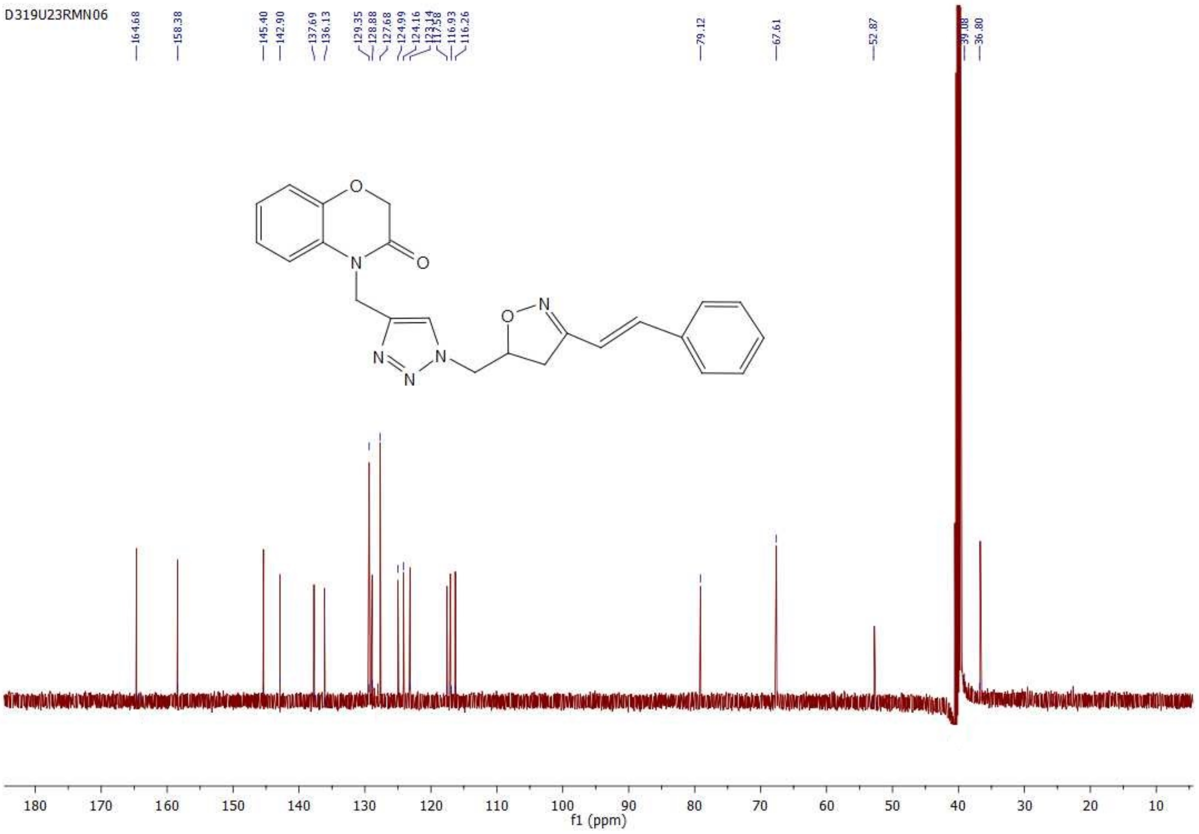

Figure S29: *^{13}C NMR spectrum of the compound* ***5h***

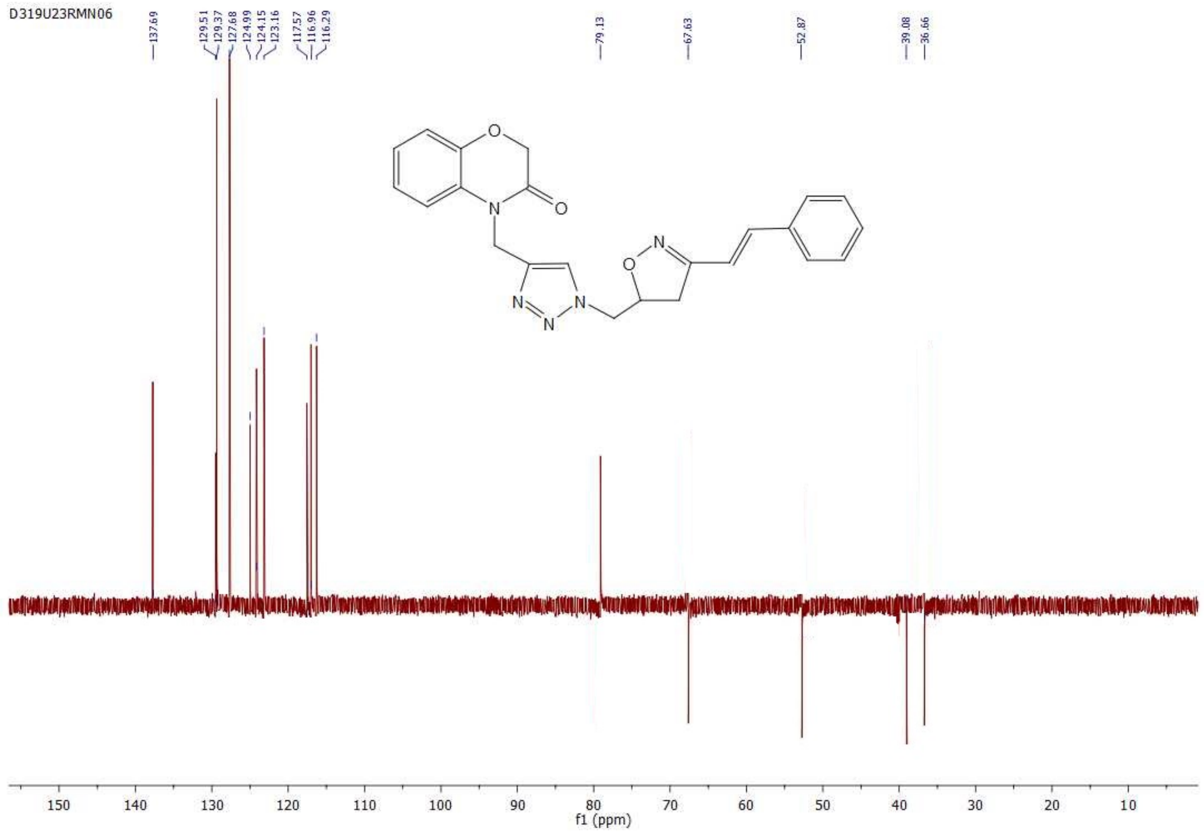

Figure S30: *DEPT-135 spectrum of the compound* ***5h***

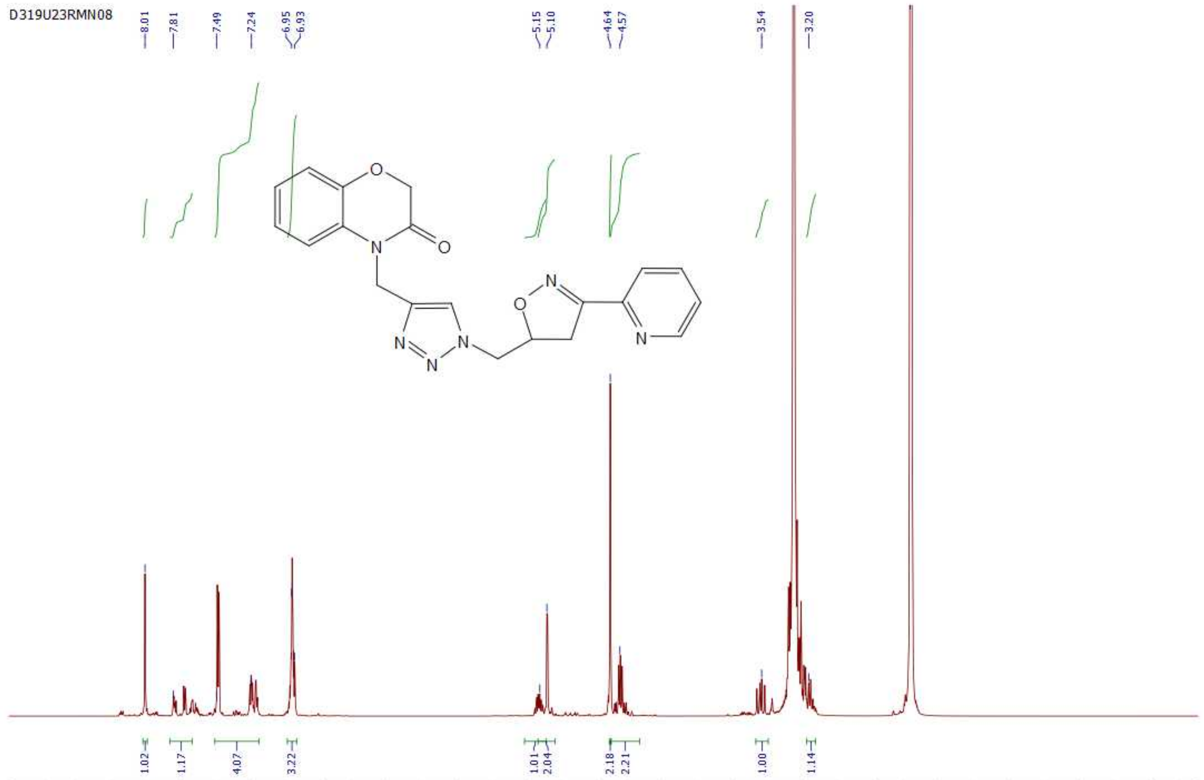

Figure S31: *^{1}H NMR spectrum of the compound* ***5i***

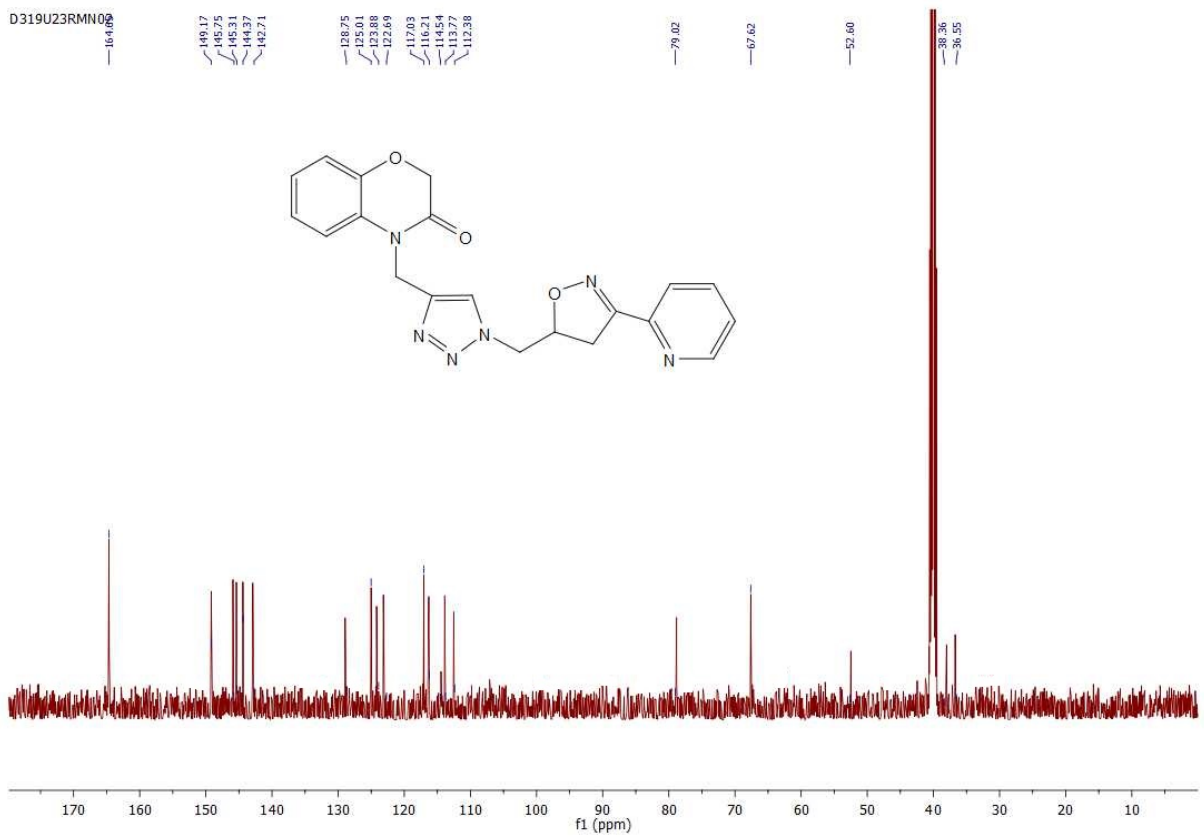

Figure S32: *^{13}C NMR spectrum of the compound* ***5i***

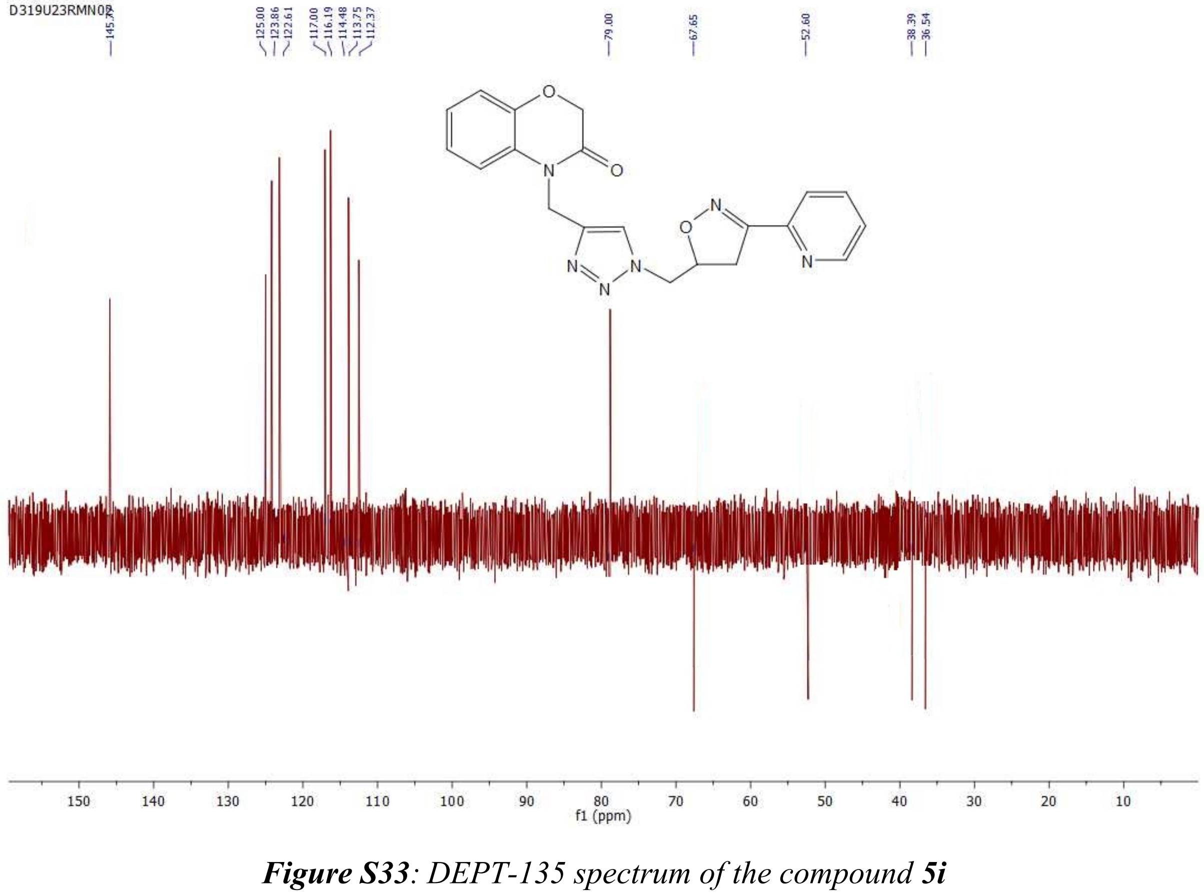

Figure S33: *DEPT-135 spectrum of the compound* ***5i***

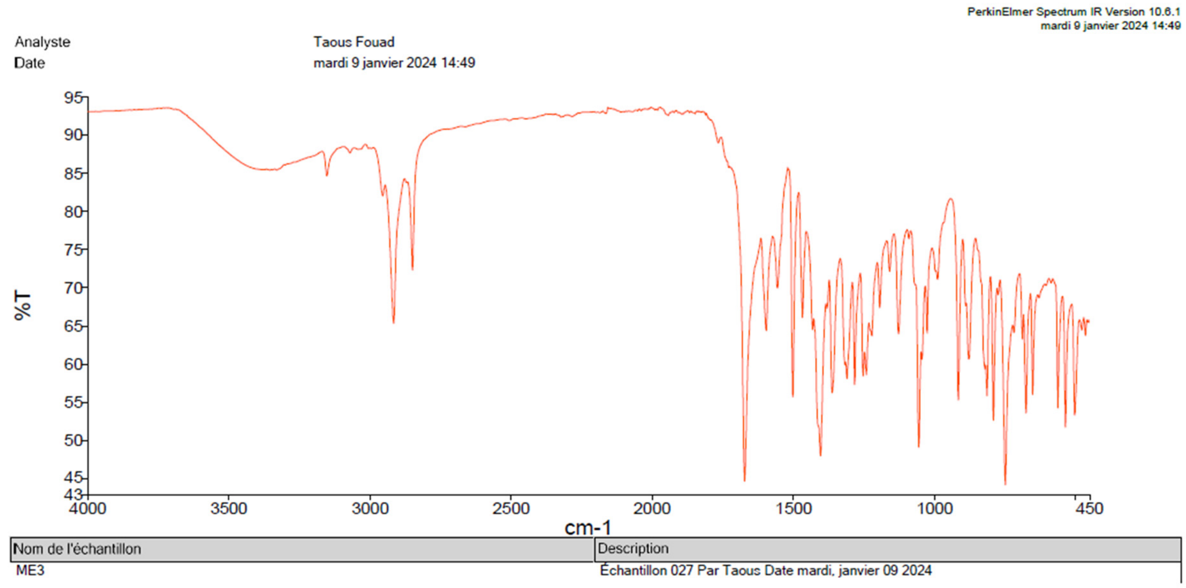

Figure S34: *IR spectrum of the compound* ***5i***

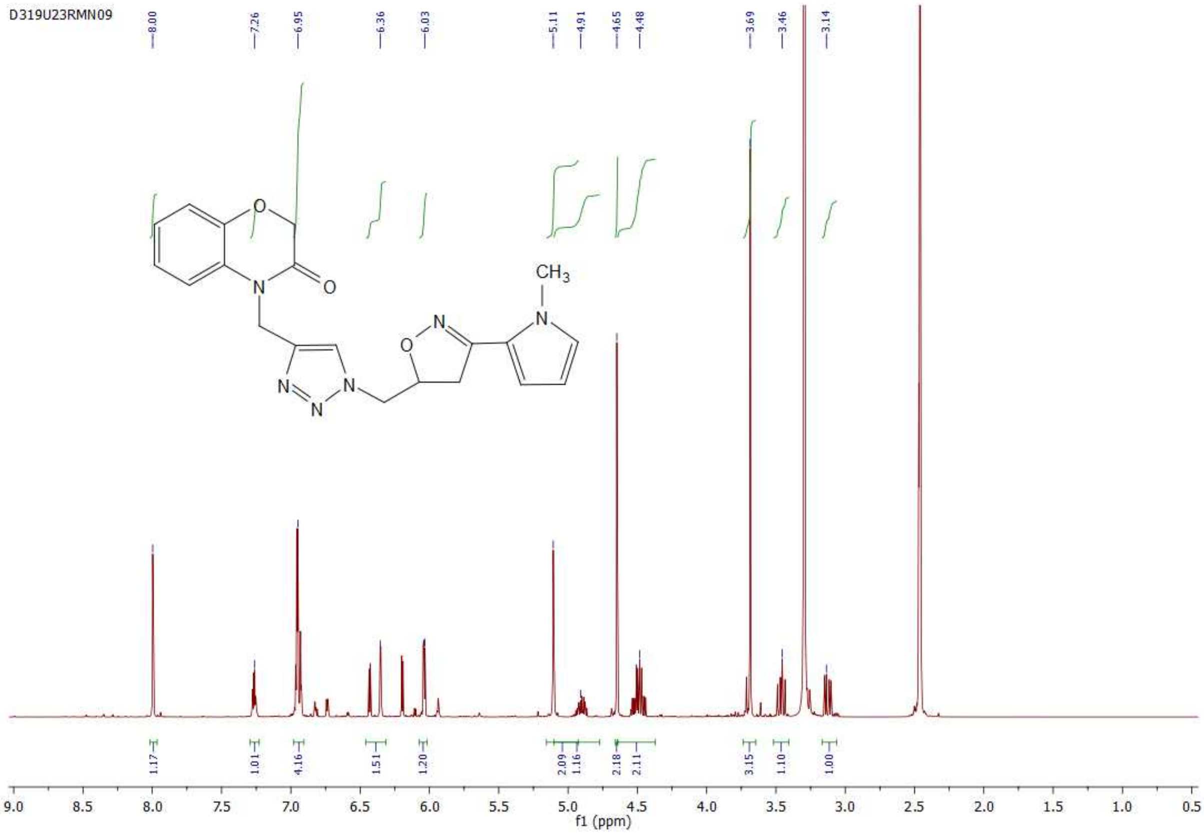

Figure S35: *^{1}H NMR spectrum of the compound* ***5j***

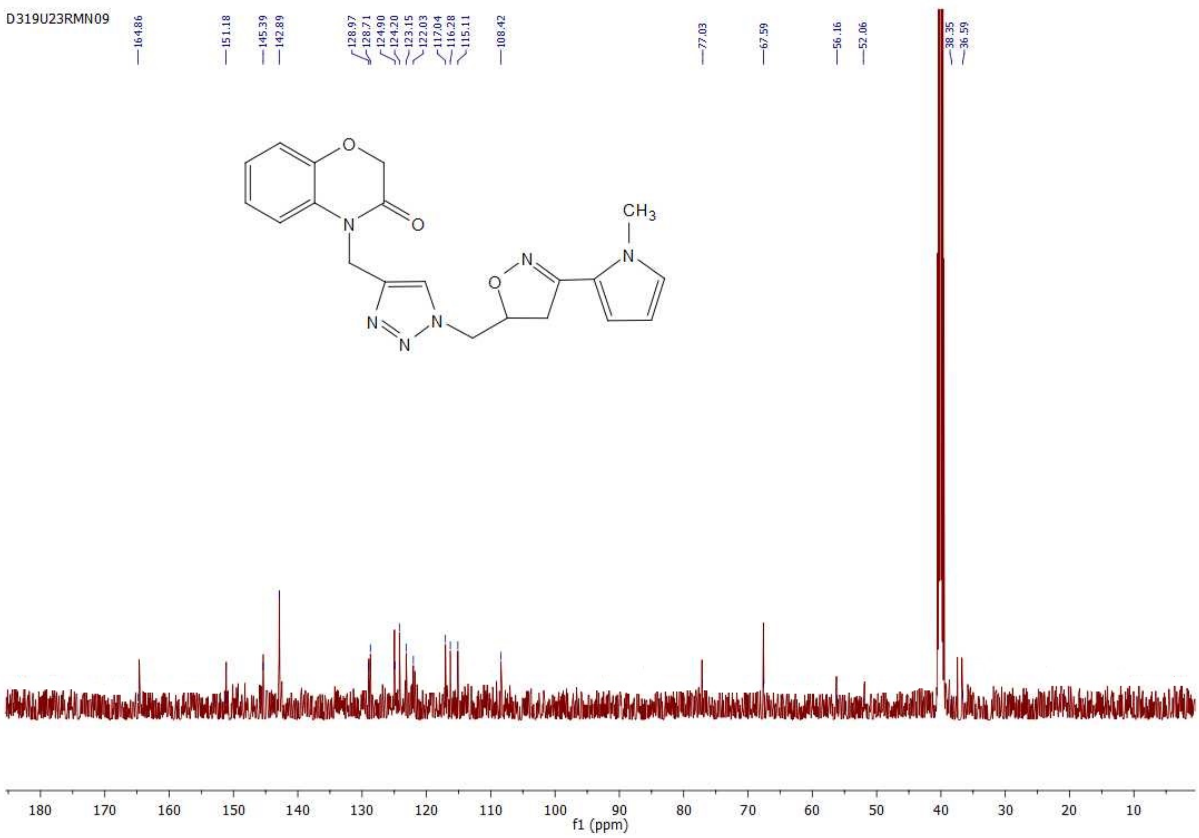

Figure S36: *^{13}C NMR spectrum of the compound* ***5j***

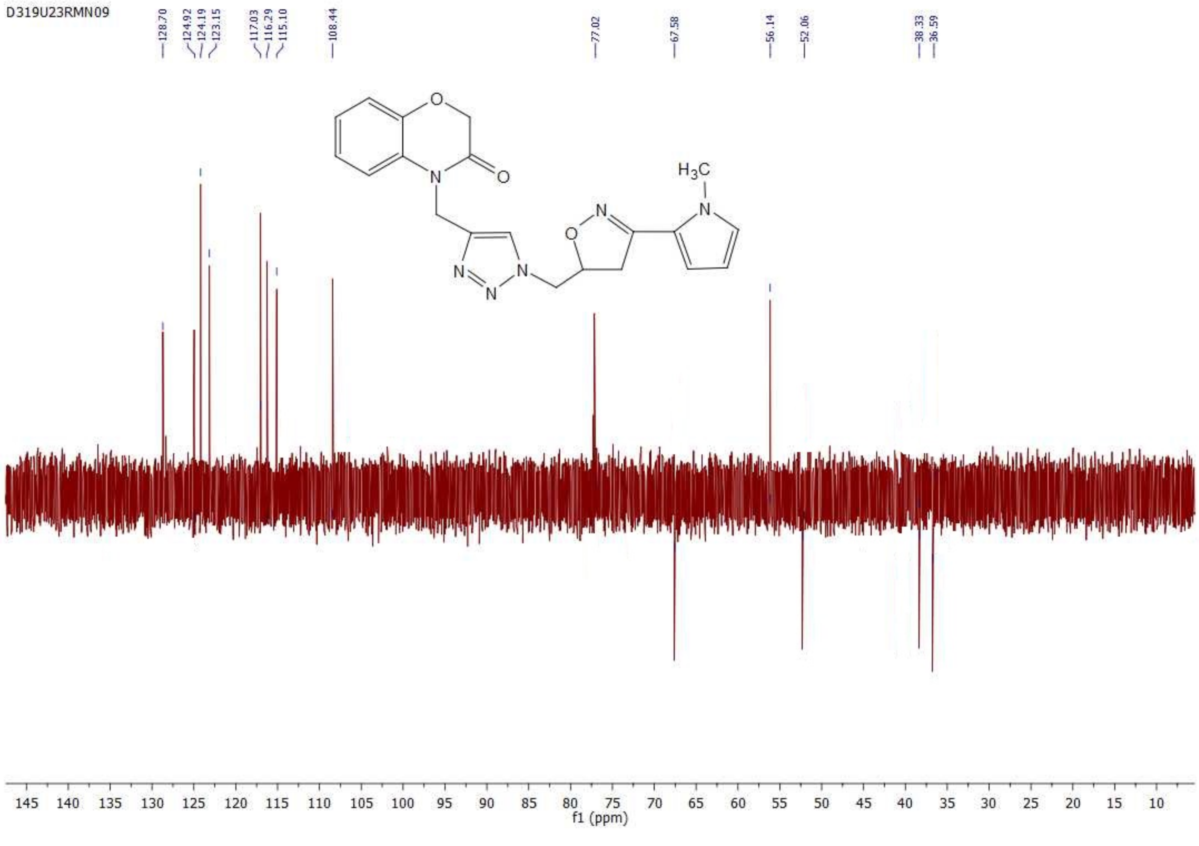

Figure S37: *DEPT-135 spectrum of the compound* ***5j***

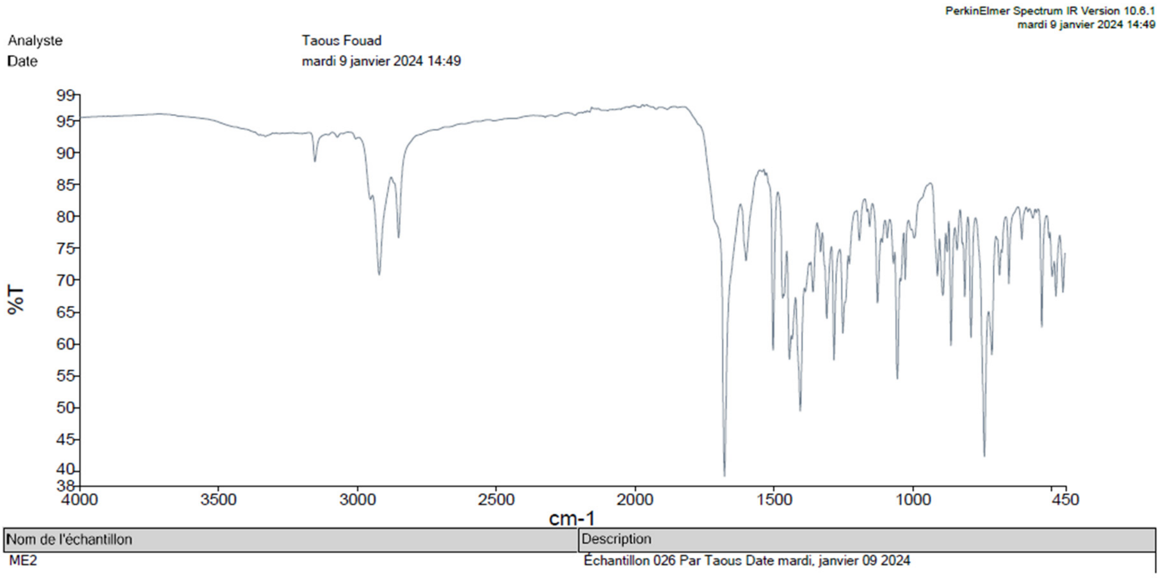

Figure S38*: IR spectrum of the compound* ***5j***

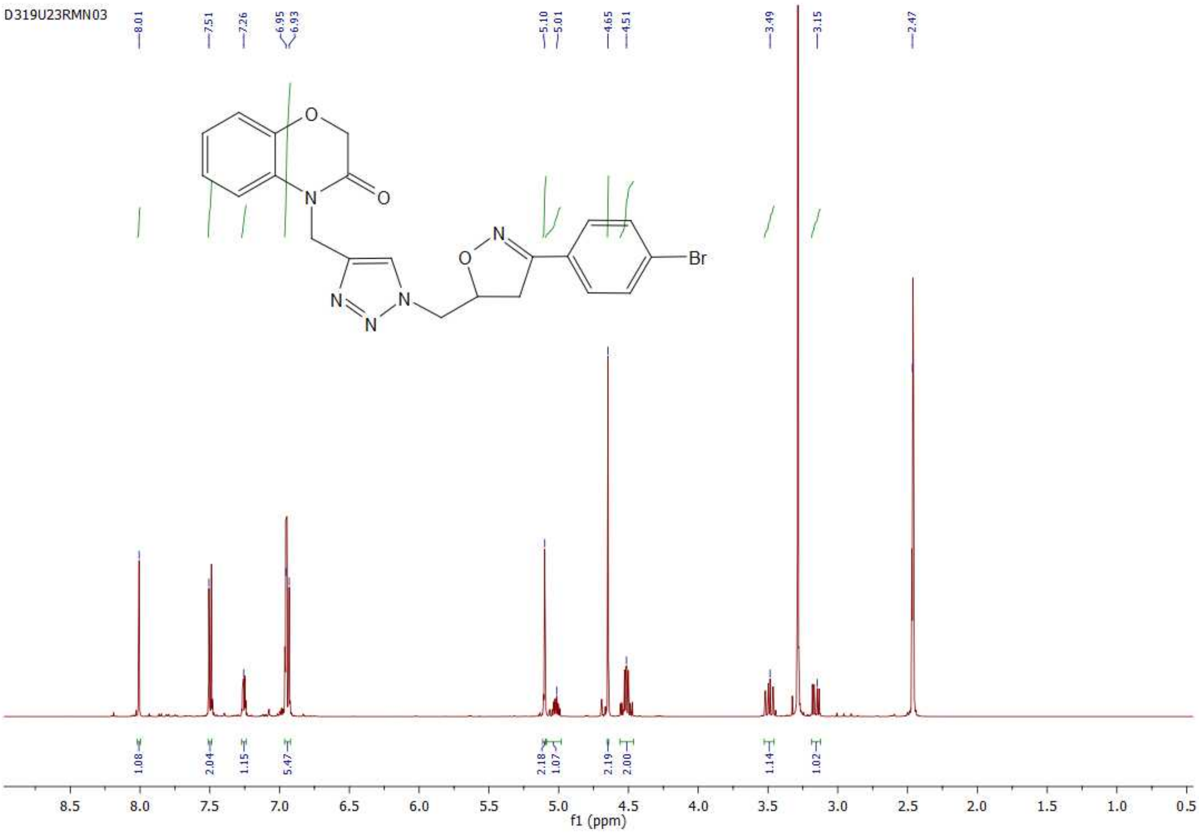

Figure S39*: ^{1}H NMR spectrum of the compound* ***5k***

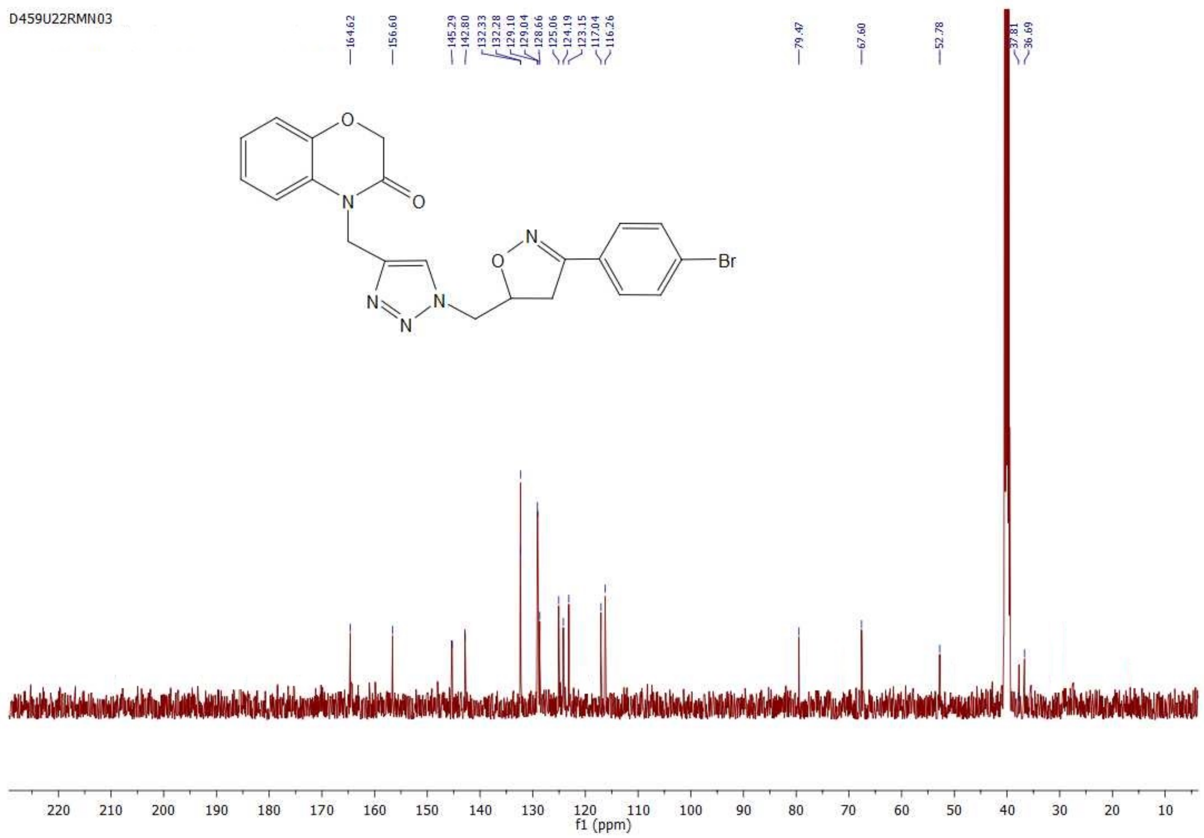

Figure S40: *^{13}C NMR spectrum of the compound* ***5k***

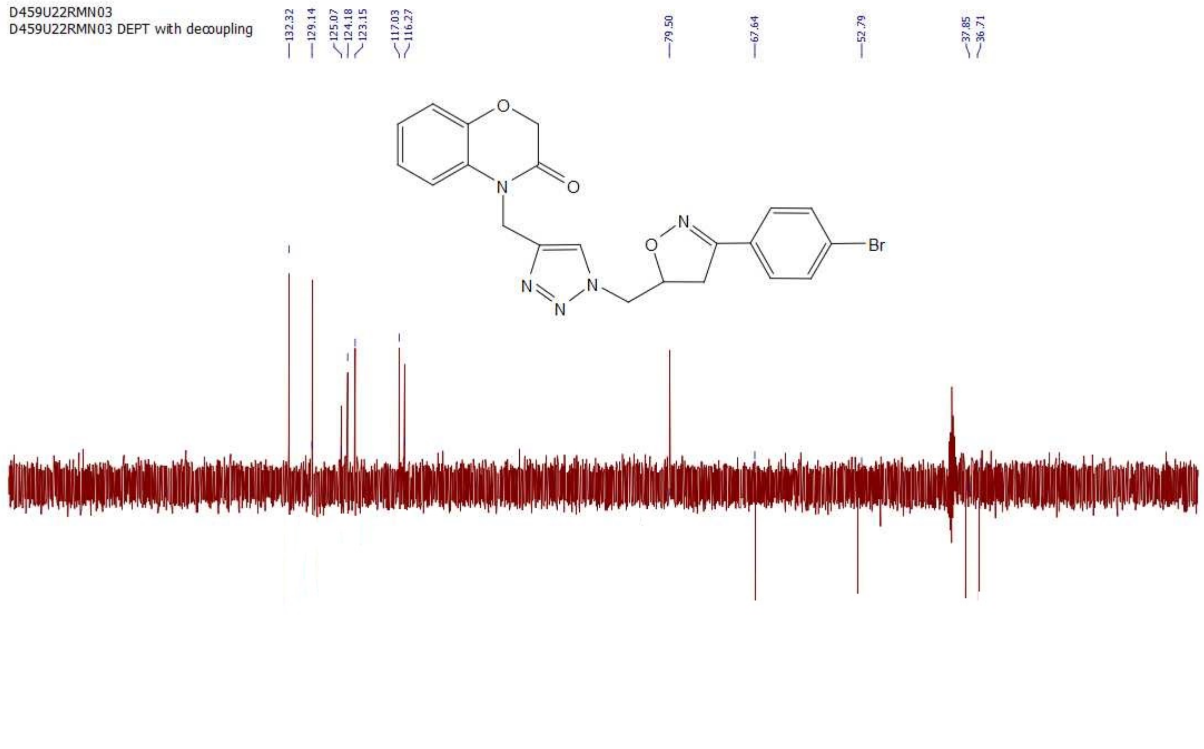

Figure S41: *DEPT-135 spectrum of the compound* ***5k***

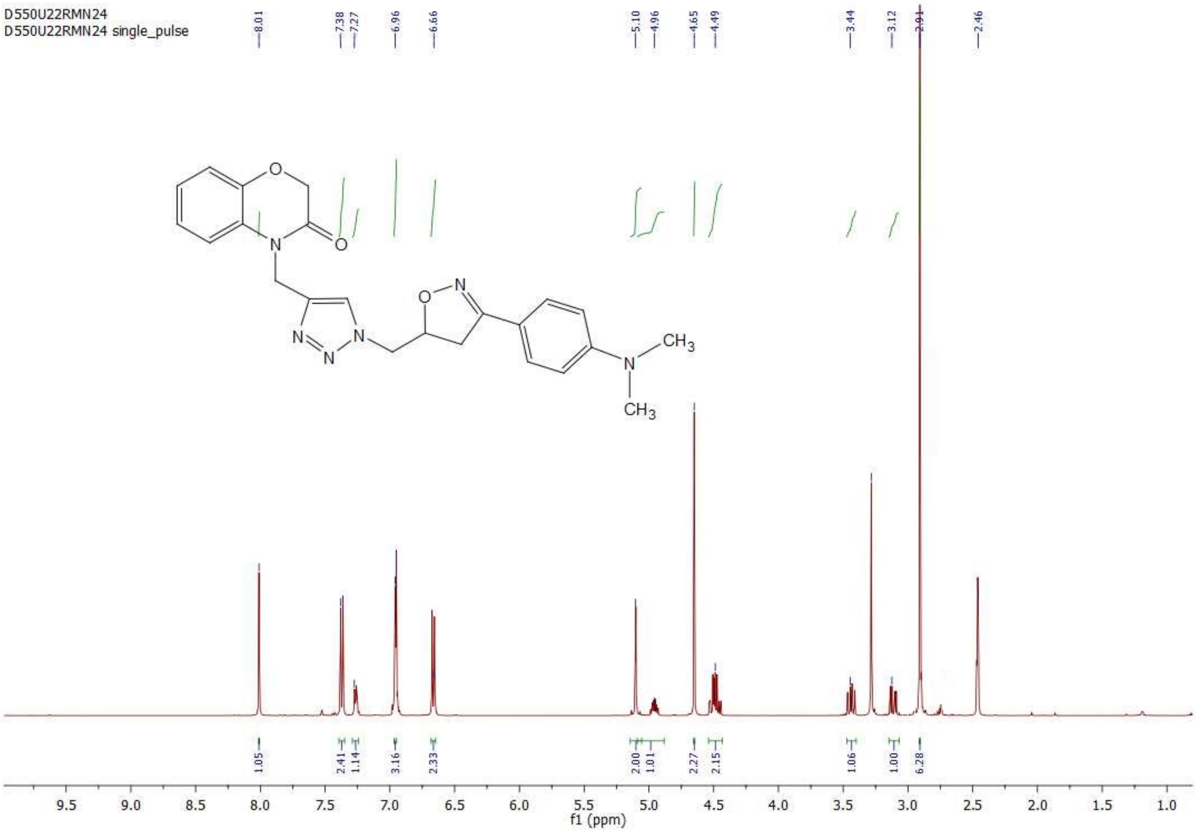

Figure S42: *^{1}H NMR spectrum of the compound* ***5l***

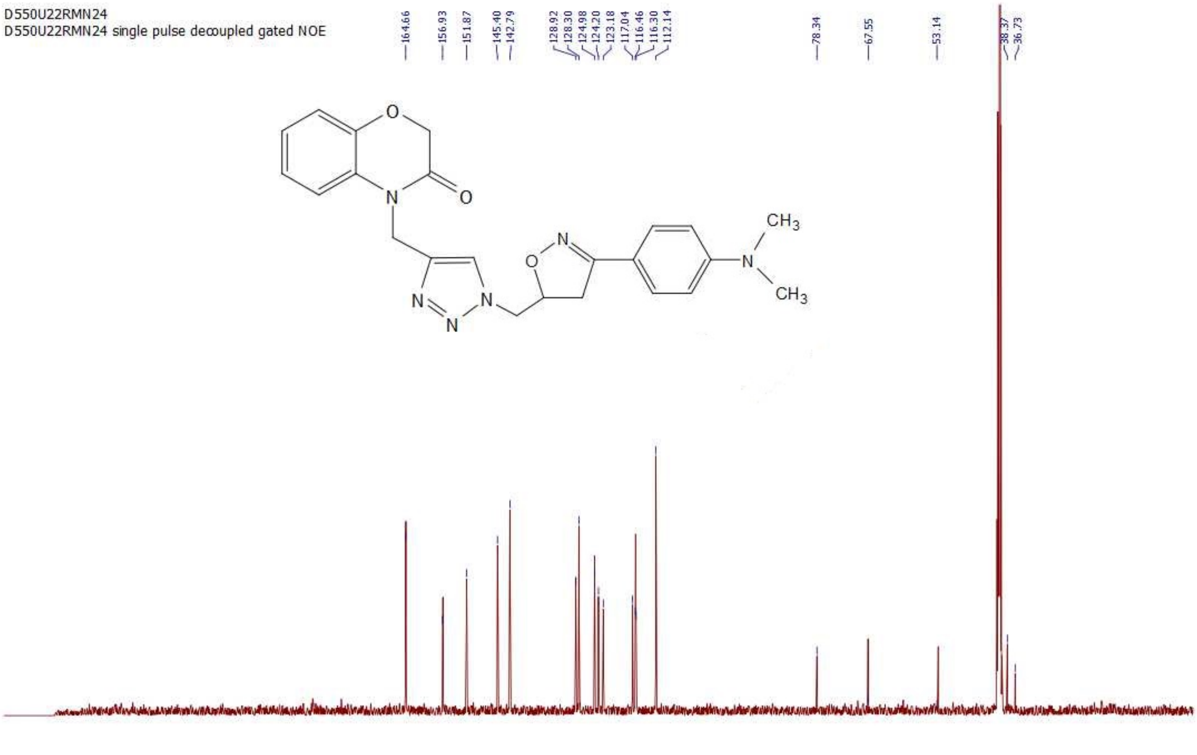

Figure S43: *^{13}C NMR spectrum of the compound* ***5l***

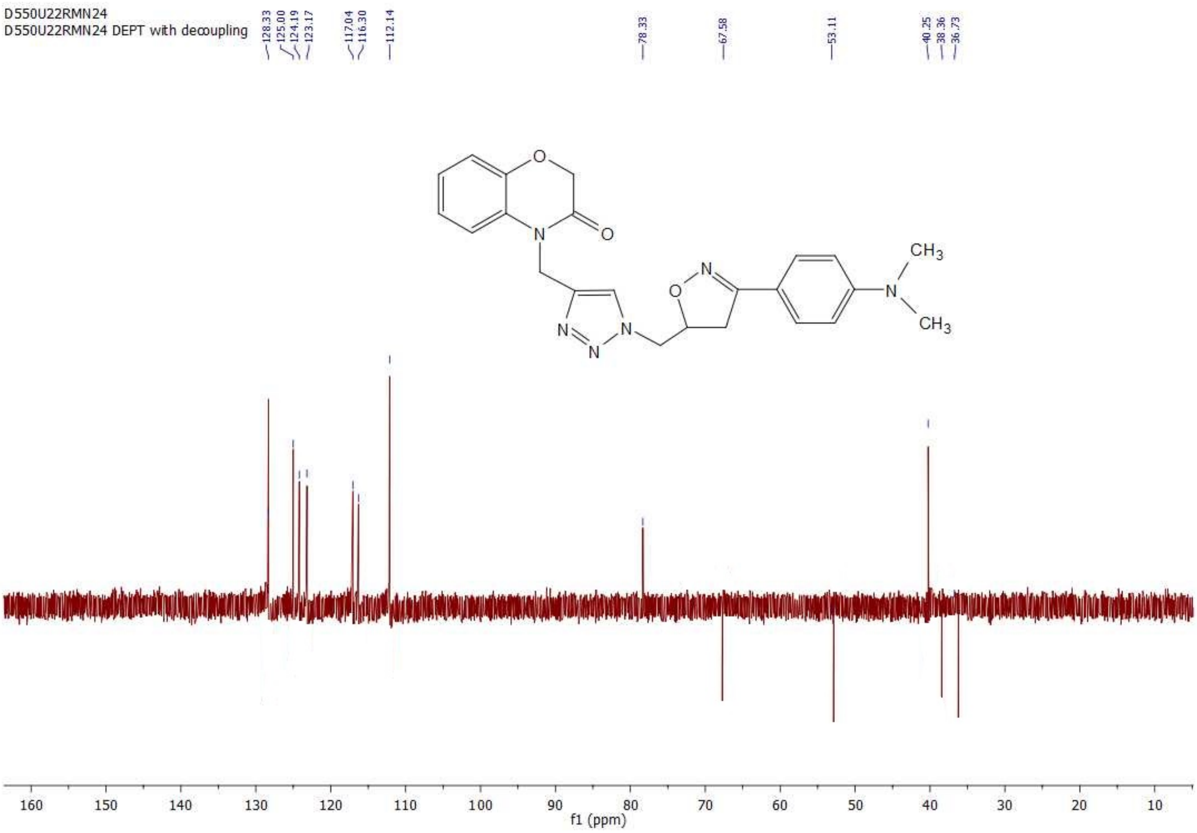

Figure S44: *DEPT-135 spectrum of the compound* ***5l***

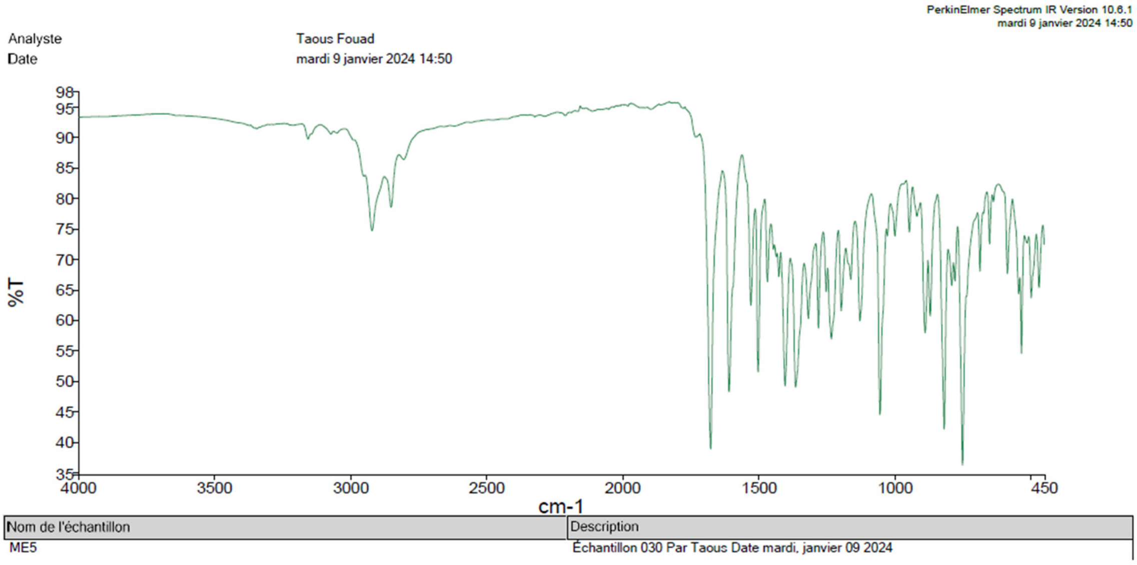

Figure S45: *IR spectrum of the compound* ***5l***

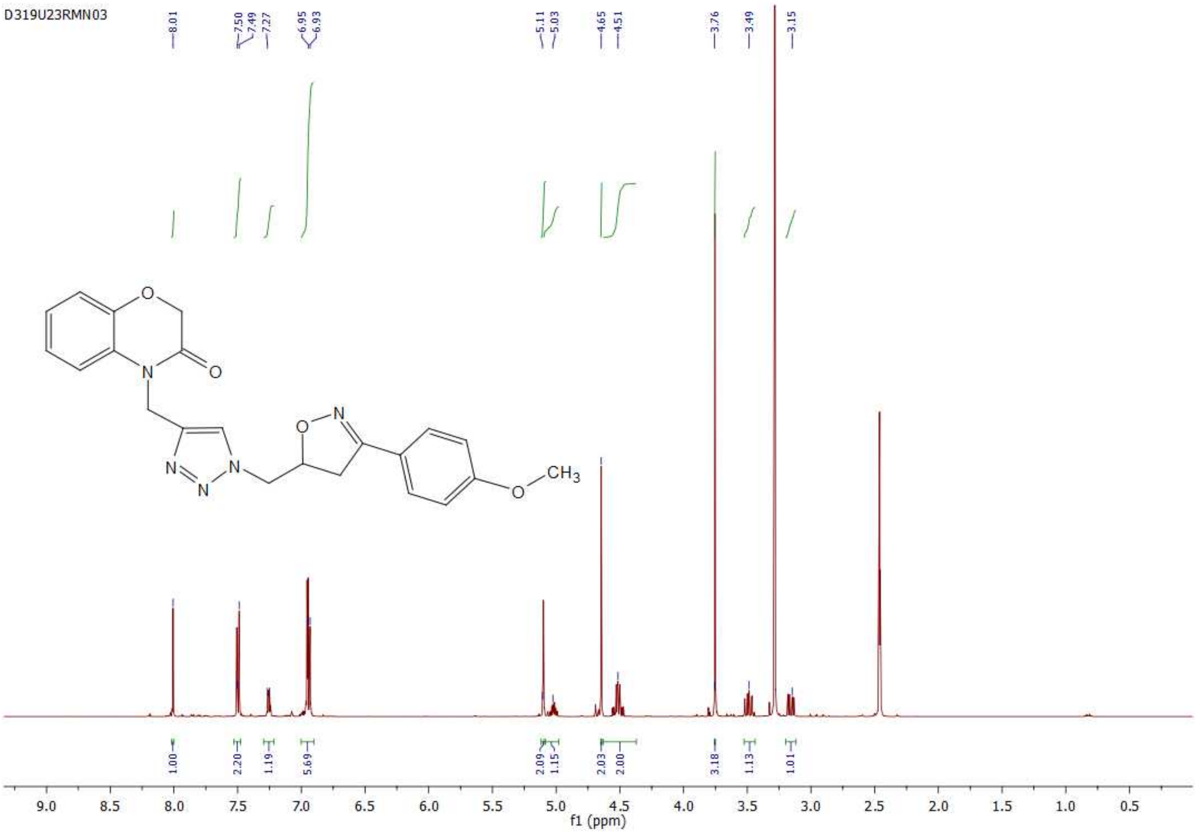

Figure S46: *^{1}H NMR spectrum of the compound* ***5m***

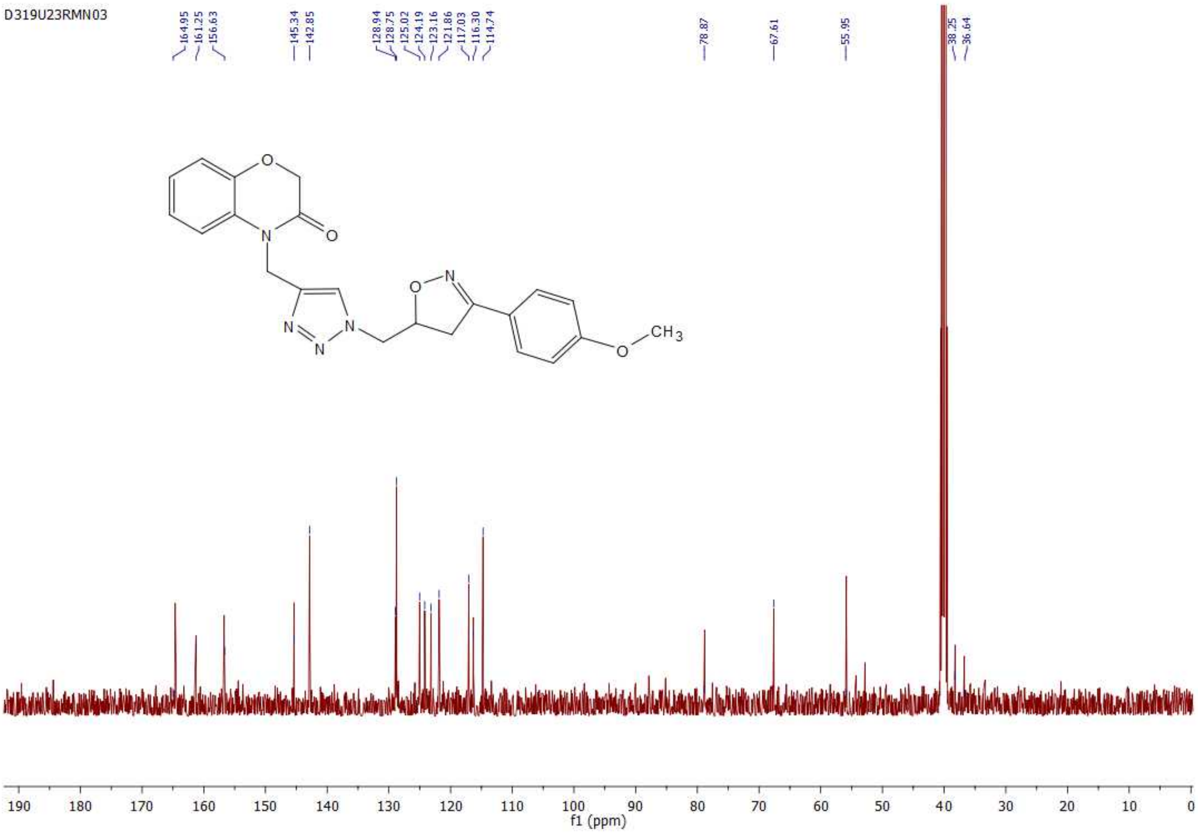

Figure S47: *^{13}C NMR spectrum of the compound* ***5m***

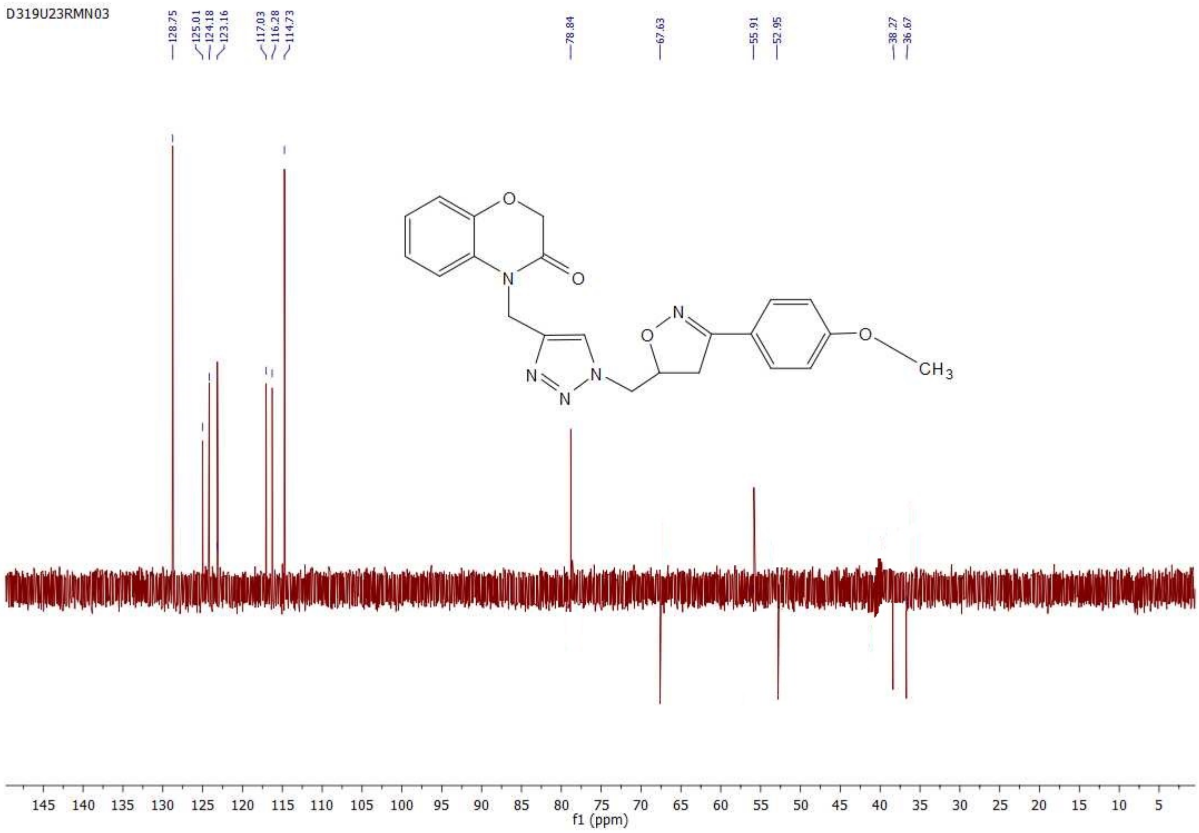

Figure S48**: DEPT-135 spectrum of the compound **5m

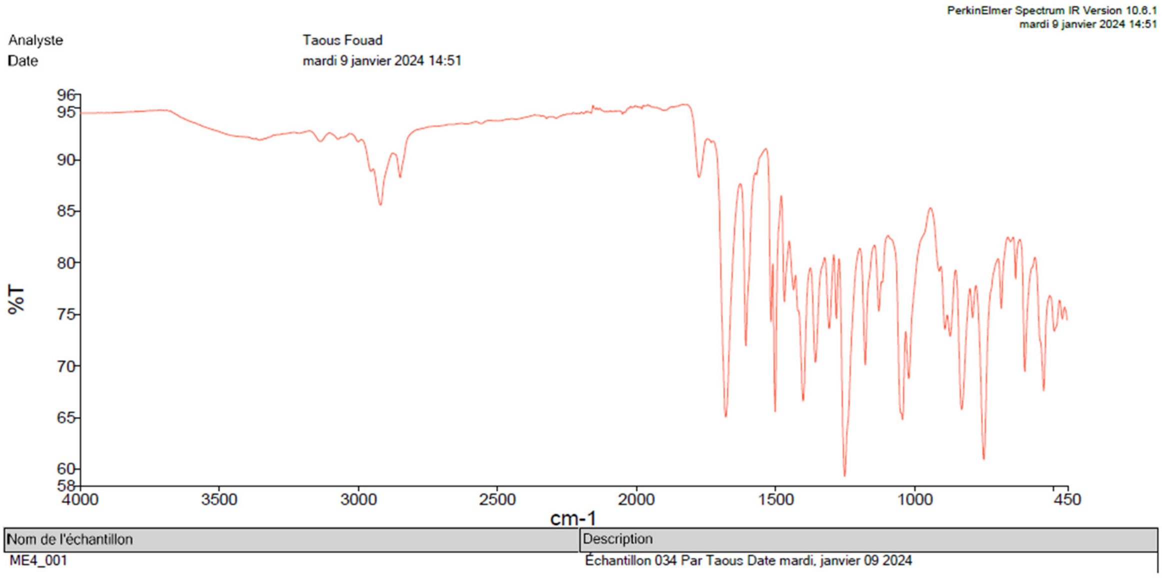

Figure S49**: IR spectrum of the compound **5m

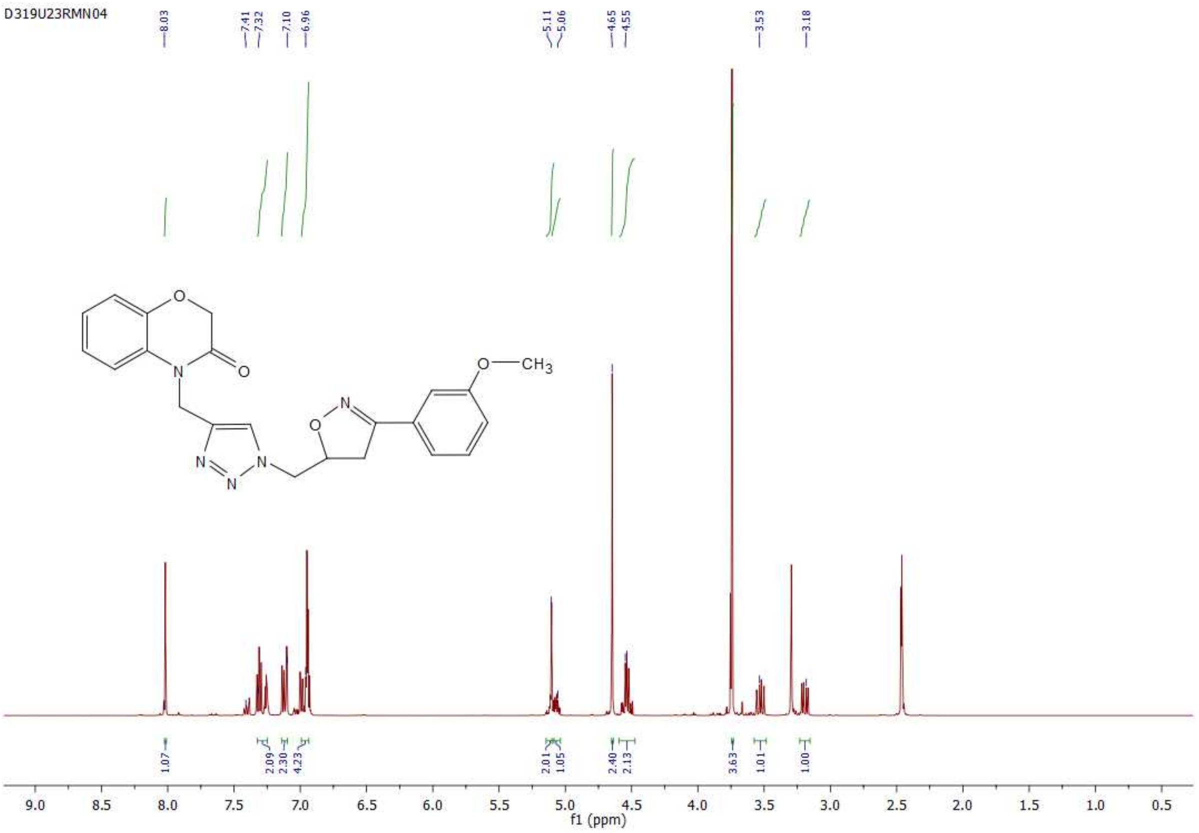

Figure S50: *^{1}H NMR spectrum of the compound* ***5n***

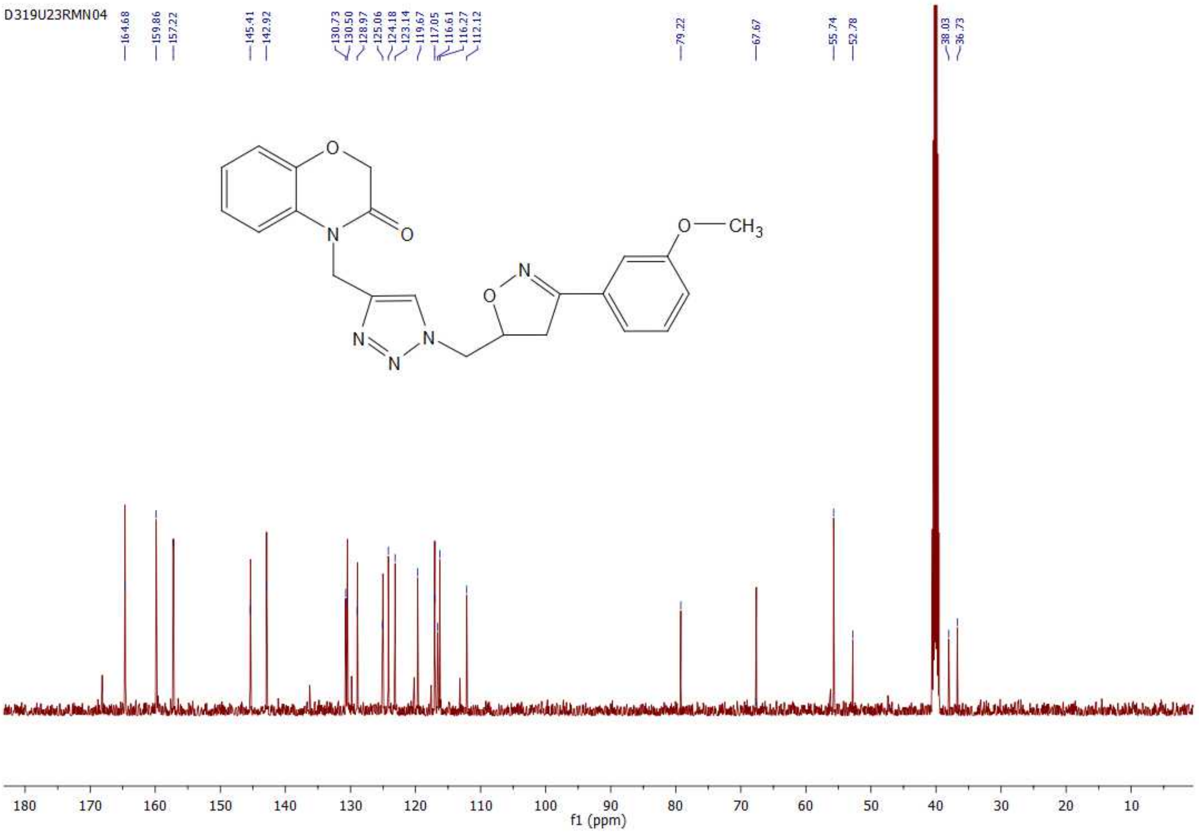

Figure S51: *^{13}C NMR spectrum of the compound* ***5n***

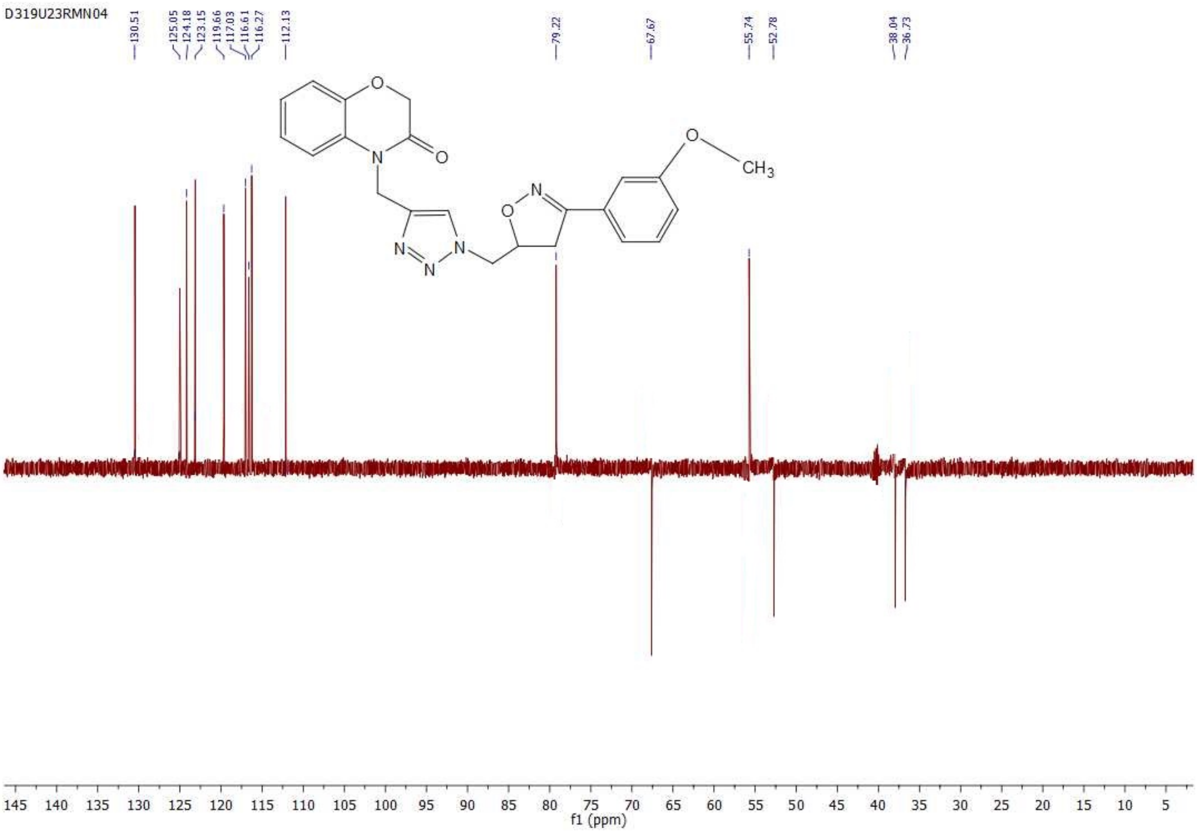

Figure S52**: DEPT-135 spectrum of the compound **5n

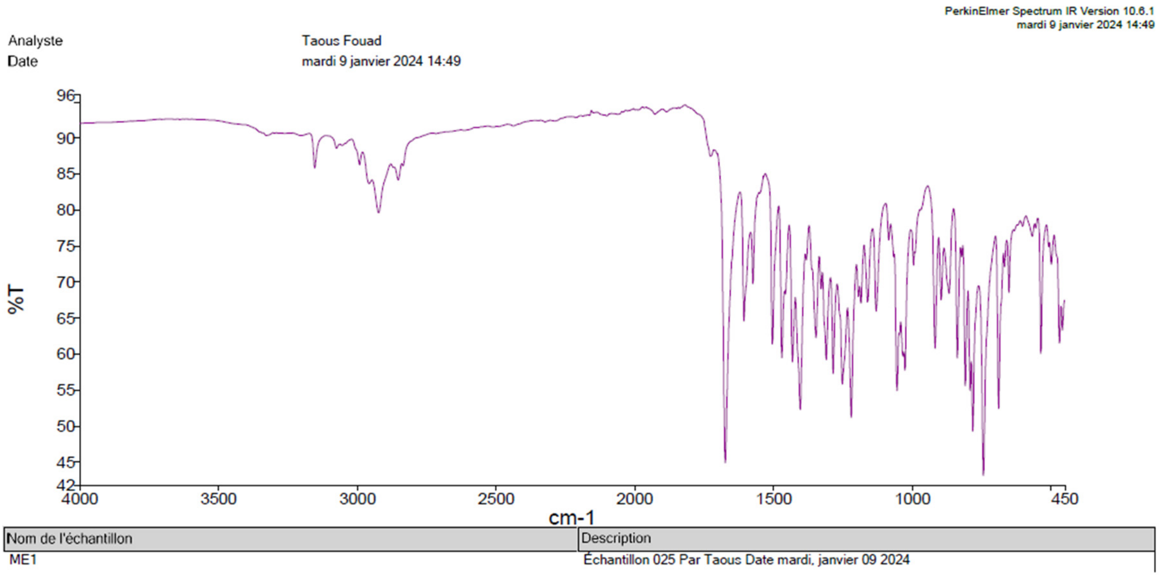

Figure S53**: IR spectrum of the compound **5n

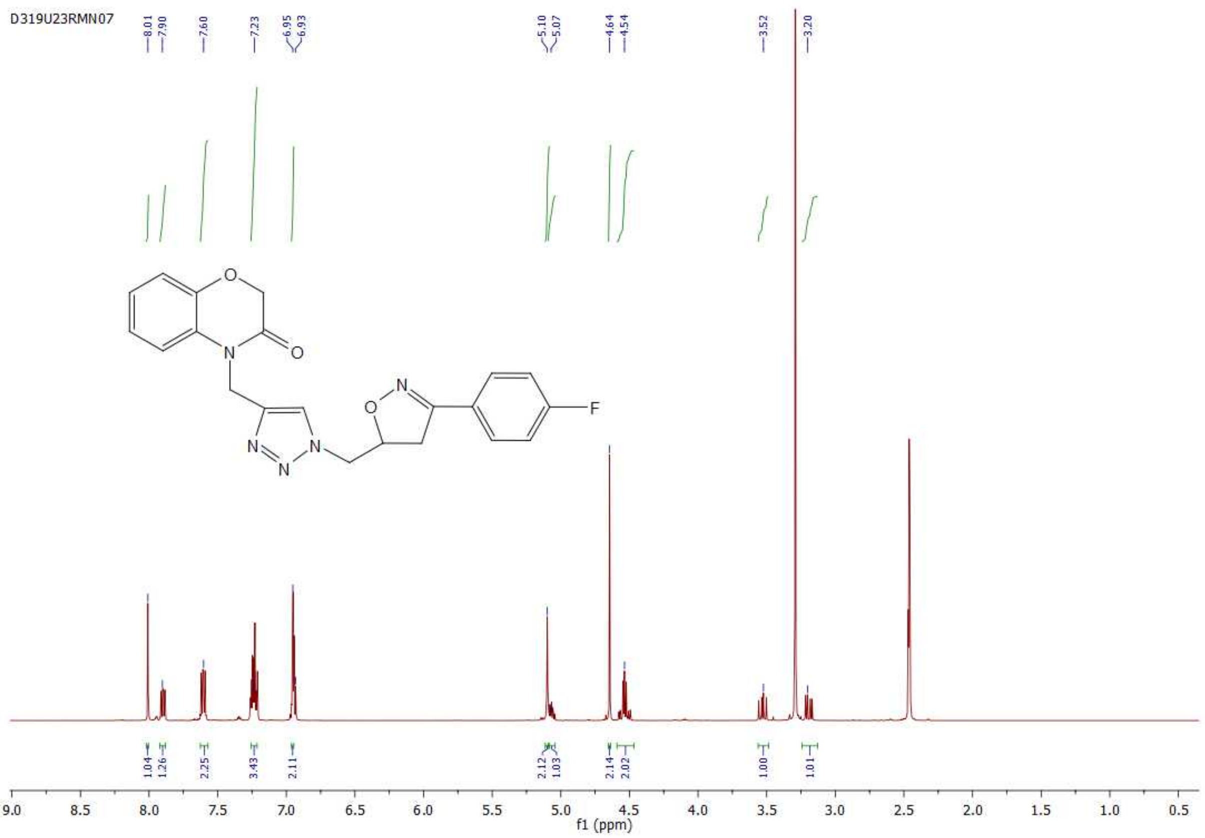

Figure S54**: ^{1}H NMR spectrum of the compound **5o

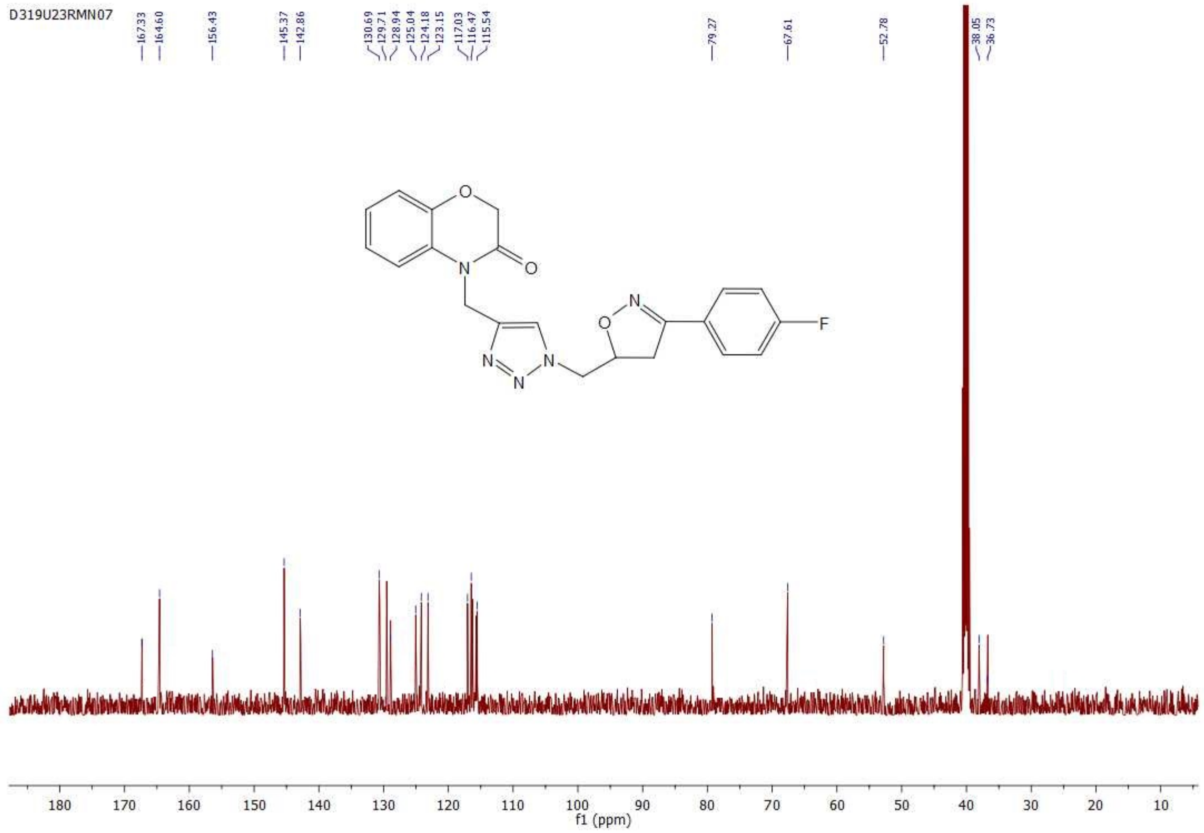

Figure S55**: ^{13}C NMR spectrum of the compound **5o

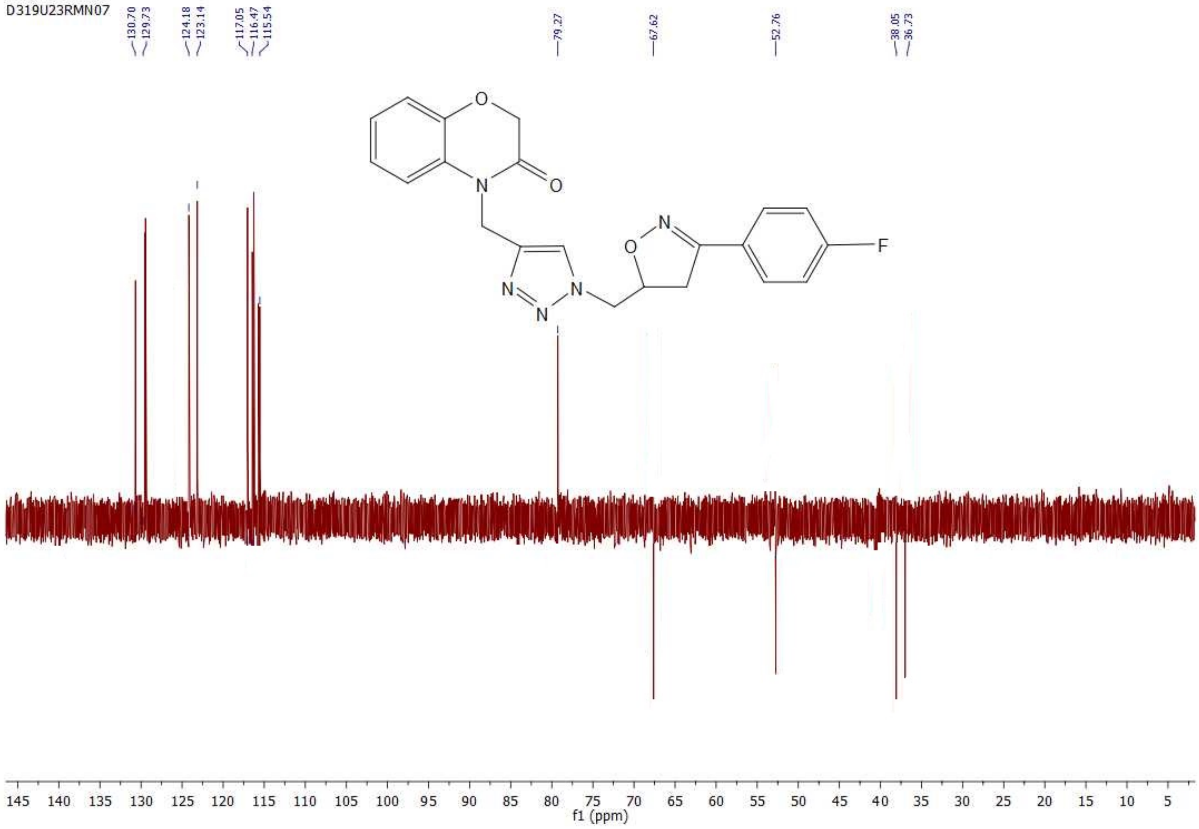

Figure S56**: DEPT-135 spectrum of the compound* ***5o
